# Supplementary material for: Influence of GST- and P450-based metabolic resistance to pyrethroids on blood feeding in the major African malaria vector Anopheles funestus
Source: PLoS One. 2020 Sep 18;15(9):e0230984. doi: 10.1371/journal.pone.0230984 (PMC7500606; doi:10.1371/journal.pone.0230984)
Supplement: S1 Table — (DOCX) [file pone.0230984.s001.docx]

**Table S1: List of primers used for qPCR analyses**

| Gene ID | Gene name | Forward sequence | Reverse sequence | Amplicon size (bp) |
| --- | --- | --- | --- | --- |
| AFUN006819 | **Act: Actin 5C** | **5’TTAAACCCAAAAGCCAATCG 3’** | **5’TCCGAGTTCATTTCCAGCTC 3’** | **111** |
| AFUN007153 | ***RSP7*: 40S Ribosomal protein S7** | **5’ GTGTTCGGTTCCAAGGTGAT 3’** | **5’ TCCGAGTTCATTTCCAGCTC 3’** | **98** |
| AFUN010733 | **AAPP: Anopheline antiplatelet protein** | **5’AAGTCCTTCGTGTTGGCTCG 3’** | **5’ ACATCCTTTGCCAGAGAGCT 3’** | **119** |
| AFUN016457 | ***D7r1*: D7 salivary related protein 1** | **5’ CCAGTAGGTAAACGAGCGAATG 3’** | **5’CAGCTTCTCTACTGCACCACT 3’** | **150** |
| AFUN016455 | ***D7r2*: D7 salivary related protein 2** | **5’GCGAACACCTTCTATACGTGC 3’** | **5’ACGGATCACTCAGTCGCATC 3’** | **112** |
| AFUN016454 | ***D7r3*: D7 salivary related protein 3** | **5’ACCTTCTATACGTGCTTCTTGGG 3’** | **5’ACGGATCACTCAGTCGCATC 3’** | **106** |
| AFUN016456 | ***D7r4*: D7 salivary related protein 4** | **5’AGGAATTGGTGAAGGCTGGA 3’** | **5’CTTTCTCACTTTCCGACGGC 3’** | **150** |
