# Supplementary material for: Influence of GST- and P450-based metabolic resistance to pyrethroids on blood feeding in the major African malaria vector Anopheles funestus
Source: PLoS One. 2020 Sep 18;15(9):e0230984. doi: 10.1371/journal.pone.0230984 (PMC7500606; doi:10.1371/journal.pone.0230984)
Supplement: Attachment — Submitted filename: PONE-D-20-07208_reviewer.pdf [file pone.0230984.s002.pdf]

PLOS ONE

Influence of GST- and P450-based metabolic resistance to pyrethroids on blood feeding in the major African malaria vector Anopheles funestus

--Manuscript Draft--

**Manuscript Number:**	PONE-D-20-07208
**Article Type:**	Research Article
**Full Title:**	Influence of GST- and P450-based metabolic resistance to pyrethroids on blood feeding in the major African malaria vector Anopheles funestus
**Short Title:**	Bloodfeeding process and insecticide resistance in Anopheles funestus mosquito
**Corresponding Author:**	Lynda DJOUNKWA NOUAGE, Msc<br>Centre for research in Infectious diseases (CRID)<br>Yaounde, CAMEROON
**Keywords:**	Malaria, Mosquito, Anopheles funestus, Insecticide resistance, Metabolic resistance, GST, P450, Blood meal intake
**Abstract:**	Insecticide resistance genes are often associated with pleiotropic effects on various mosquito life-history traits. However, very little information is available on the impact of insecticide resistance on blood feeding process in mosquitoes. Here, using two recently detected DNA-based metabolic markers in the major malaria vector, An. funestus, we investigated how metabolic resistance genes could affect blood meal intake.<br>After allowing both the field F1 and lab F8 Anopheles funestus strains to feed on the human arm for 30 minutes, we assessed the association between key parameters of blood meal process including, probing time, feeding duration, blood feeding success, blood meal size, and markers of glutathione S-transferase (L119F-GSTe2) and cytochrome P450 (CYP6P9a_R) - mediated metabolic resistance. None of the parameters of blood meal process was associated with L119F-GSTe2 genotypes. In contrast, for CYP6P9a_R, homozygote resistant mosquitoes were significantly more able to blood-feed than homozygote susceptible (OR = 3.3; CI 95%: 1.4-7.7; P =0.01) mosquitoes. Moreover, the volume of blood meal ingested by CYP6P9a-SS mosquitoes was lower than that of CYP6P9a-RS (P<0.004) and of CYP6P9a-RR (P<0.006). This suggests that CYP6P9a gene affects the feeding success and blood meal size of An. funestus. However, no correlation was found in the expression of CYP6P9a and that of genes encoding for salivary proteins involved in blood meal process.<br>This study suggests that P450-based metabolic resistance may increase the blood feeding ability of malaria vectors and potentially impacting their vectorial capacity.
**Order of Authors:**	Lynda NOUAGE, Msc<br>Emmanuel ELANGA-NDILLE<br>Achille Binyang<br>Magellan Tchouakui<br>Tatiane ATSATSE<br>Cyrille Ndo<br>Sévilor KEKEUNOU<br>Charles S. Wondji
**Additional Information:**	
**Question**	**Response**
**Financial Disclosure**<br><br>Enter a financial disclosure statement that describes the sources of funding for the	The funders had no role in study design, data collection and analysis, decision to publish, or preparation of the manuscript

Powered by Editorial Manager® and ProduXion Manager® from Aries Systems Corporation

work included in this submission. Review the submission guidelines for detailed requirements. View published research articles from *PLOS ONE* for specific examples.<br><br>This statement is required for submission and **will appear in the published article** if the submission is accepted. Please make sure it is accurate.<br><br>**Unfunded studies**<br>Enter: *The author(s) received no specific funding for this work.*<br><br>**Funded studies**<br>Enter a statement with the following details:<br>• Initials of the authors who received each award<br>• Grant numbers awarded to each author<br>• The full name of each funder<br>• URL of each funder website<br>• Did the sponsors or funders play any role in the study design, data collection and analysis, decision to publish, or preparation of the manuscript?<br>• **NO** - Include this sentence at the end of your statement: *The funders had no role in study design, data collection and analysis, decision to publish, or preparation of the manuscript.*<br>• **YES** - Specify the role(s) played.<br><br>* typeset	
**Competing Interests**<br><br>Use the instructions below to enter a competing interest statement for this submission. On behalf of all authors, disclose any competing interests that could be perceived to bias this work—acknowledging all financial support and any other relevant financial or non-financial competing interests.<br><br>This statement **will appear in the published article** if the submission is accepted. Please make sure it is accurate. View published research articles from *PLOS ONE* for specific examples.	The authors have declared that no competing interests exist.

**NO authors have competing interests**<br><br>Enter: *The authors have declared that no competing interests exist.*<br><br>**Authors with competing interests**<br><br>Enter competing interest details beginning with this statement:<br><br>*I have read the journal's policy and the authors of this manuscript have the following competing interests: [insert competing interests here]*<br><br>* typeset	
**Ethics Statement**<br><br>Enter an ethics statement for this submission. This statement is required if the study involved:<br><br>• Human participants<br>• Human specimens or tissue<br>• Vertebrate animals or cephalopods<br>• Vertebrate embryos or tissues<br>• Field research<br><br>Write "N/A" if the submission does not require an ethics statement.<br><br>General guidance is provided below. Consult the submission guidelines for detailed instructions. **Make sure that all information entered here is included in the Methods section of the manuscript.**	NA

Powered by Editorial Manager® and ProduXion Manager® from Aries Systems Corporation

**Format for specific study types**<br><br>**Human Subject Research (involving human participants and/or tissue)**<br>• Give the name of the institutional review board or ethics committee that approved the study<br>• Include the approval number and/or a statement indicating approval of this research<br>• Indicate the form of consent obtained (written/oral) or the reason that consent was not obtained (e.g. the data were analyzed anonymously)<br><br>**Animal Research (involving vertebrate animals, embryos or tissues)**<br>• Provide the name of the Institutional Animal Care and Use Committee (IACUC) or other relevant ethics board that reviewed the study protocol, and indicate whether they approved this research or granted a formal waiver of ethical approval<br>• Include an approval number if one was obtained<br>• If the study involved *non-human primates*, add *additional details* about animal welfare and steps taken to ameliorate suffering<br>• If anesthesia, euthanasia, or any kind of animal sacrifice is part of the study, include briefly which substances and/or methods were applied<br><br>**Field Research**<br><br>Include the following details if this study involves the collection of plant, animal, or other materials from a natural setting:<br>• Field permit number<br>• Name of the institution or relevant body that granted permission	
**Data Availability**<br><br>Authors are required to make all data underlying the findings described fully available, without restriction, and from the time of publication. PLOS allows rare exceptions to address legal and ethical concerns. See the PLOS Data Policy and FAQ for detailed information.	Yes - all data are fully available without restriction

Powered by Editorial Manager® and ProduXion Manager® from Aries Systems Corporation

A Data Availability Statement describing where the data can be found is required at submission. Your answers to this question constitute the Data Availability Statement and **will be published in the article**, if accepted.<br><br>**Important:** Stating 'data available on request from the author' is not sufficient. If your data are only available upon request, select 'No' for the first question and explain your exceptional situation in the text box.<br><br>Do the authors confirm that all data underlying the findings described in their manuscript are fully available without restriction?	
**Describe where the data may be found in full sentences. If you are copying our sample text, replace any instances of XXX with the appropriate details.**<br><br>• If the data are **held or will be held in a public repository**, include URLs, accession numbers or DOIs. If this information will only be available after acceptance, indicate this by ticking the box below. For example: *All XXX files are available from the XXX database (accession number(s) XXX, XXX.).*<br>• If the data are all contained **within the manuscript and/or Supporting Information files**, enter the following: *All relevant data are within the manuscript and its Supporting Information files.*<br>• If neither of these applies but you are able to provide **details of access elsewhere**, with or without limitations, please do so. For example:<br><br>*Data cannot be shared publicly because of [XXX]. Data are available from the XXX Institutional Data Access / Ethics Committee (contact via XXX) for researchers who meet the criteria for access to confidential data.*<br><br>*The data underlying the results presented in the study are available from (include the name of the third party*	All relevant data are within the manuscript and its Supporting Information files

and contact information or URL).<br>• This text is appropriate if the data are owned by a third party and authors do not have permission to share the data.<br><br>* typeset	
Additional data availability information:	

Powered by Editorial Manager® and ProduXion Manager® from Aries Systems Corporation

Manuscript

Click here to access/download;Manuscript;Nouage et al_Manuscript .docx

Influence of GST- and P450-based metabolic resistance to pyrethroids on blood feeding in the major African malaria vector *Anopheles funestus*

Lynda NOUAGE[1,2¥*], Emmanuel ELANGA-NDILLE[1¥*], Achille BINYANG[1,2], Magellan TCHOUAKUI[1,2], Tatiane ATSATSE[1,2], Cyrille NDO[3, 4,] , Sévilor KEKEUNOU[2], Charles S. WONDJI[1,5]

[1]Departement of Medical Entomology, Centre for Research in Infectious Diseases (CRID) P.O. BOX 13591, Yaoundé, Cameroon

[2]Department of Animal Biology and Physiology, Faculty of Science, University of Yaoundé 1, P.O. Box 812, Yaoundé, Cameroon

[3]Departement of Parasitology and Microbiology, Centre for Research in Infectious Diseases (CRID) P.O. BOX 13591, Yaoundé, Cameroon

[4]Department of Biological Sciences, Faculty of Medicine and Pharmaceutical Sciences, University of Douala, P.O. Box 24157, Douala, Cameroon.

[5]Department of Vector Biology, Liverpool School of Tropical Medicine, Pembroke Place, Liverpool L3 5QA, UK

¥: These authors contributed equally to this work

*: Corresponding authors: Emmanuel ELANGA-NDILLE (emmsdille@yahoo.fr; emmanuel.elanga@crid-cam.net) and Lynda NOUAGE (lnouage@gmail.com)

Abstract

Insecticide resistance genes are often associated with pleiotropic effects on various mosquito life-history traits. However, very little information is available on the impact of insecticide resistance on blood feeding process in mosquitoes. Here, using two recently detected DNA-based metabolic markers in the major malaria vector, *An. funestus,* we investigated how metabolic resistance genes could affect blood meal intake.

After allowing both the field F1 and lab F8 *Anopheles funestus* strains to feed on the human arm for 30 minutes, we assessed the association between key parameters of blood meal process including, probing time, feeding duration, blood feeding success, blood meal size, and markers of glutathione S-transferase (*L119F-GSTe2*) and cytochrome P450 (*CYP6P9a_R*) - mediated metabolic resistance. None of the parameters of blood meal process was associated with *L119F-GSTe2* genotypes. In contrast, for *CYP6P9a*_R, homozygote resistant mosquitoes were significantly more able to blood-feed than homozygote susceptible (OR = 3.3; CI 95%: 1.4-7.7; P =0.01) mosquitoes. Moreover, the volume of blood meal ingested by CYP6P9a-SS mosquitoes was lower than that of CYP6P9a-RS (P<0.004) and of CYP6P9a-RR (P<0.006). This suggests that *CYP6P9a* gene affects the feeding success and blood meal size of *An. funestus*. However, no correlation was found in the expression of *CYP6P9a* and that of genes encoding for salivary proteins involved in blood meal process.

This study suggests that P450-based metabolic resistance may increase the blood feeding ability of malaria vectors and potentially impacting their vectorial capacity.

Keywords: Malaria, Mosquito, *Anopheles funestus*, Insecticide resistance, Metabolic resistance, GST, P450, Blood meal intake

Introduction

Malaria remains a major public health scourge in sub-Sahara Africa despite significant progress made since the 2000s in reducing its burden [1]. This disease is caused by a Plasmodium parasite transmitted by *Anopheles* mosquito species while taking a blood meal on humans. Blood feeding, is essential for female mosquito's fecundity [2], as *Anopheles* species like all anautogenous female mosquitoes, require a blood meal to obtain amino acids needed to synthesize yolk proteins for eggs maturation [3,4].

Mosquito's blood feeding success is facilitated by the pharmacological proprieties of salivary gland proteins [5]. Indeed, some salivary proteins such as anopheline antiplatelet protein (AAPP), apyrase, gSG6 and members of D7 family have been identified as vasodilators, anti-coagulants and inhibitors of platelet aggregation allowing mosquitoes to overcome host haemostatic mechanisms and to have a successful blood meal [5-8].

Mosquito's fecundity was shown to vary by source and size of the blood meal with a difference of these two parameters resulting in significant variations of the number of eggs laid by each female mosquito [4,8]. It has been shown that the number of eggs laid per female is positively associated to the amount of blood ingested as larger blood meals resulted in an increase of the number of females that developed eggs and the number of eggs per female [9,10]. The volume of blood taken by a mosquito could be affected by a range of intrinsic (host immunity) and extrinsic factors including ambient temperatures, mosquito age, parity status, gonotrophic cycle, blood feeding history and infection status [10]. More recently, it was reported that exposure to pyrethroids could also significantly influence the blood meal process and the blood meal volume ingested by *Anopheles gambiae* mosquito [11].

Pyrethroids (PY) are the insecticide class mostly used in the last two decades through ITNs and IRS strategies to control malaria transmission [12]. Unfortunately, the widespread use of these insecticides has favoured the development of resistance in malaria vector species [13,14].

Resistance to pyrethroids involves two main mechanisms: (i) metabolic resistance, due to the increase expression level of detoxifying enzymes, belonging to three families: the cytochrome P450 monooxygenases, the glutathione S-transferases and the carboxylesterases; and (ii) target-site resistance due to mutations in the voltage sodium channels known as knock-down (kdr) mutations [15,16]. Although resistance mechanisms help mosquitoes to survive under continuous insecticide pressure, these actions are costly and may negatively affect mosquito's fitness including body size, adult longevity, larval development time, fecundity, fertility, mating competitiveness and blood feeding capability [17-19]. For target-site resistance, a decreased longevity and an increased larval development time have been reported in *kdr*-pyrethroid-resistant mosquitoes [20,21]. Moreover, a recent study suggested that kdr-based resistance could impact blood feeding with heterozygote (*kdr*-RS) and susceptible (*kdr*-SS) mosquitoes taking higher blood volume than homozygote (*kdr*-RR) resistant individuals [11]. In contrast, little is known on the impact of metabolic resistance as DNA-based markers were not previously available for this mechanism; thereby limiting the ability to investigate its physiological impact on the blood feeding process in mosquitoes. However, taking advantage of the identification of the first DNA-based metabolic marker in *An. funestus* mosquito, one study reported that a GST-based metabolic resistance caused by a leucine to phenylalanine amino acid change at codon 119 in the glutathione S-transferase epsilon 2 *(L119F-GSTe2)* [22], has a detrimental impact on *An. funestus* fitness as field mosquitoes exhibited a reduced fecundity and slower larval development but an increased adult longevity [23]. On the other hand, a new DNA-based assay was recently designed for cytochrome P450-mediated resistance (the *CYP6P9a*-R) in *An. funestus.* This marker showed that mosquitoes carrying this P450-resistant allele survived and succeeded in blood feeding more often than did susceptible mosquitoes when exposed to insecticide-treated nets [24]. The design of assays for both GST- and P450-based resistance now offers a great opportunity to explore how the blood feeding

process is influenced by metabolic resistance mechanism in malaria vectors and further assess how resistance may impact the vectorial capacity of mosquitoes to transmit malaria in the natural environment.

Here, we investigated the effect of metabolic resistance to pyrethroids on the blood feeding process in *An. funestus*, using the two DNA-based metabolic resistance markers: *L119F-GSTe2* and *CYP6P9a*-R [22,24]. Specifically, we assessed the association between the genotypes of these metabolic resistance markers and key parameters of blood feeding including mosquito probing time, feeding duration and the blood meal size.

Material and Methods

Mosquito collection and rearing

Experiments were carried out using both field and lab strains of *An. funestus*. Field mosquitoes (F_1) were generated from indoor resting female (F0) collected in Mibellon (6°46’N, 11^0 70’E), a village located in a rural area of the savanna-forest region in Cameroon, Central Africa where the *L119F-GSTe2* has been reported [25]. Blood-fed field collected females were kept in paper cups and transported to the insectary of the Centre for Research in Infectious Diseases (CRID) in Yaoundé where they were kept for 4–5 days until they became fully gravid and were then induced to lay eggs using the forced eggs-laying method [26]. The eggs were placed in paper cups containing water to hatch, after which the larvae were transferred in to trays and reared to adults. To assess the effect of *CYP6P9a* marker, F_8 progenies were generated from reciprocal crosses established between the pyrethroid susceptible laboratory strain (FANG) and the resistant (FUMOZ-R) lab strain. These two *An. funestus* lab strains were colonized from mosquitoes collected in Southern Africa region. FUMOZ is a pyrethroid resistant strain

established in the insectary from wild-caught *An. funestus* mosquito species from southern Mozambique [27]. The p revious study reported that the over-expression of two duplicated P450 genes, *CYP6P9a* and *CYP6P9b*, constitute the main mechanism driving pyrethroid resistance in this strain [28,29] for which the *119F-GSTe2* allele is absent [22]. The FANG strain is completely susceptible to pyrethroids colonized from Calueque in southern Angola [27].

Blood feeding experiments and blood meal size quantification

Blood feeding process: Since blood meal volume has previously been reported to correlate with mosquito size [2], individuals used for blood feeding experiments were first grouped according to their size. Mosquito size was determined by weighing (using an analytical micro-scale, SARTORIUS, Goettingen, Germany) each individual (adult females aged 3-7 days) starved for 24h and immobilized by chilling them for 2 minutes at 5°C. Each mosquito was then placed in paper cups for about an hour before given a blood meal. Mosquitoes were allowed to bite for 30 min on the bare forearm of a single human volunteer after informed consent.

The duration of probing and blood feeding was assessed using a batch of 120 F_1 female field-collected mosquitoes. For this purpose, mosquitoes were individually transferred in polystyrene plastic cups covered with net. They were allowed to rest for 15 min before observations began. During the blood intake, each mosquito was filmed with a Digital HD Video Camera (Canon PC2154, Canon INC, Japan) placed beside the plastic cup. At the end of the time allowed for feeding, the film for each mosquito was analysed and the parameters such as probing time (defined as the time taken from initial insertion of the mouthparts in the skin until the initial engorgement of blood) [5] and total feeding duration, were recorded, using a digital timer. Due to the low density of female mosquitoes obtained at F_8 generation from reciprocal crosses of the lab strain mosquitoes, experiments to estimate the probing and the feeding duration of this strain were not investigated.

To determine the blood meal size for both strains, batches of 25 mosquitoes grouped according to their weight were allowed to bite on a human arm. In this case, neither the probing time nor the feeding duration was recorded. After the trial, the whole abdomen of successfully fed mosquitoes (evident by red-coloration engorgement of the abdomen) was extracted and stored in an individual 1.5 ml microtube at – 20°C to measure the blood meal size. The rest of the carcasses as well as unfed mosquitoes were kept individually in a microtube containing RNA-later and stored at -20°C.

Blood meal size quantification

The volume of blood ingested by each mosquito was determined by quantifying the haemoglobin amount, as previously described [30]. Briefly, abdomen of blood fed mosquitoes were homogenized in 0.5 ml of Drabkin's reagent which converts the haemoglobin into haemoglobin cyanide (HiCN). After 20 minutes at room temperature and the addition of 0.5 ml of chloroform solution, samples were centrifuged at 5600 rpm for 5 min. The aqueous supernatant containing HiCN was transferred in a new 1.5 ml microtube. An aliquot of 200µl from each sample was transferred to a microplate and the optical density (OD) read at a wavelength of 620nm in a spectrophotometer (EZ Read 400, biochrom, Cambridge, UK). OD for each sample were read in duplicate and the average value between the two replicates was considered as OD value of the sample. In parallel, OD read on various amounts of human volunteer blood added to Drabkin's reagent in individual microtubes were used as control to generate calibration curves and the regression line used to assess the relationship between OD and blood volume. For each sample, the blood meal size was estimated according to the weight by dividing the blood volume estimated using the regression line by the average weigh of each batch of mosquitoes constituted after the weighing. The blood meal size was then expressed in µL of blood per mg of weight.

Molecular species identification

Genomic DNA (gDNA) was extracted from both blood-fed and unfed mosquitoes using the Livak protocol [31]. Instead of using the whole body as done for unfed mosquitoes, DNA was extracted from the carcasses of fed mosquitoes after removing the abdomen for blood volume quantification. The concentration and purity of the extracted gDNA were subsequently determined using NanoDrop™ spectrophotometer (Thermo Scientific, Wilmington, USA) before storage at −20 °C. An aliquot of gDNA extracted from field-collected strain w used for molecular identification by a polymerase chain reaction [32] to determine species composition of *An. funestus* group among the samples.

Genotyping of *L119F-GSTe2* mutation in field-collected strain

The *L119F-GSTe2* mutation was genotyped using gDNA extracted from carcasses of field-collected strains following an allele specific PCR diagnostic assay previously described [23]. The primers sequences are given in table S1. PCR was performed in Gene Touch thermalcycler (Model TC-E-48DA, Hangzhou，310053，China), in a reaction volume of 15 µl using 10 µM of each primer, 10X Kapa Taq buffer A, 0.2 mM dNTPs, 1.5 mM MgCl2, 1U Kapa Taq (Kapa Biosystems, Wilmington, MA, USA) and 1µl of genomic DNA as template. The cycle parameters were: 1 cycle at 95 °C for 2 min; 30 cycles of 94 °C for 30 s, 58 °C for 30 s, 72 °C for 1 min and then a final extension at 72 °C for 10 min. PCR products were separated on 2% agarose gel by electrophoresis. The size of the diagnostic band was 523 bp for homozygous resistant (RR) and 312 bp for homozygous susceptible (SS), while heterozygous (RS) showed the two bands.

Genotyping of *CYP6P9a*-R allele in lab strain mosquitoes

The *CYP6P9a* resistance marker was genotyped using the protocol recently designed by [24]. A PCR-RFLP were carried out using gDNA extracted from the carcasses of F_8 generation individuals obtained from the reciprocal crosses between FANG and FUMOZ strains used for blood feeding. Briefly, a partial *CYP6P9a* upstream region was amplified in a final volume of 15µl PCR mixture containing 1.5µl of 10X Kapa Taq buffer A (Kapa Biosystems, Wilmington, MA, USA), 0.12µl of 5 U/µl KAPA taq, 0.12µl of 25µM dNTP, 0.75µl of 25µM MgCl2, 0.51µl of each primer, 10.49µl of dH2O and 1µl of genomic DNA. The PCR cycle parameters were as followed: the initial denaturation step at 95°C for 5 minutes followed by 35 cycles of 94°C for 30 seconds, 58°C for 30 seconds and 72°C for 45 seconds and a final extension step of 72°C for 10 minutes. The PCR products were size separated on a 1.5 % agarose gel stained with Midori Green Advance DNA Stain (Nippon genetics Europe GmbH) and visualised using a gel imaging system to confirm the product size (450bp). Then, the PCR product was incubated at 65°C for 2 hours. This was done in 0.2ml PCR strip tubes using 5µl of PCR product, 1µl of cutSmart buffer, 0.2µl of 2 units of TaqI enzyme (New England Biolabs) and 3.8µl of dH20. Size separation was done on a 2.0% agarose gel stained with Midori Green Advance DNA Stain at 100V for 30 minutes. The gel was visualised using the gel imaging system.

Gene expression profiling of major salivary genes encoding proteins involved in blood meal process.

The expression profiles of a set of salivary genes encoding for proteins involved in blood meal process was compared between CYP6P9a-RR, CYP6P9a-RS and CYP6P9a-SS *An. funestus* mosquitoes. For each gene, two pairs of exon-spanning primers was designed for each gene using Primer3 online software (v4.0.0; http://bioinfo.ut.ee/primer3/) and only primers with PCR

efficiency between 90 and 110% determined using a cDNA dilution series obtained from a single sample, were used for qPCR analysis. Taking into account this criteria of efficiency, only the AAPP and four members of D7 family genes (D7r1, D7r2, D7r3, and D7r4) were used for this analysis. Primers are listed in Table S1. Total RNA was extracted from three batches of 10 whole females of 3–5 days old from CYP6P9a-RR, CYP6P9a-RS and CYP6P9a-SS mosquitoes. RNA was isolated using the RNAeasy Mini kit (Qiagen) according to the manufacturer's instructions. The RNA quantity was assessed using a NanoDrop ND1000 spectrophotometer (Thermo Fisher) and 1µg from each of the three biological replicates for each batch of mosquitoes was used as a template for cDNA synthesis using the SuperScript III (Invitrogen, Waltham, Massachusetts, USA) with oligo-dT20 and RNase H, following the manufacturer's instructions. The qPCR assays were carried out in a MX 3005 real-time PCR system (Agilent, Santa Clara, CA 95051, United States) using Brilliant III Ultra-Fast SYBR Green qPCR Master Mix (Agilent). A total of 10 ng of cDNA from each sample was used as template in a three-step program involving a denaturation at 95 °C for 3 min followed by 40 cycles of 10 s at 95 °C and 10 s at 60 °C and a last step of 1 min at 95 °C, 30 s at 55 °C, and 30 s at 95 °C. The relative expression and fold-change of each target gene in CYP6P9a-RR and CYP6P9a-RS relative to CYP6P9a-SS was calculated according to the $2^{-\Delta\Delta CT}$ method incorporating PCR efficiency after normalization with the housekeeping RSP7 ribosomal protein S7 (VectorBase ID: AFUN007153;) and the actin 5C (vectorBase ID: AFUN006819) genes for *An. funestus*.

Statistical analysis

All analyses were conducted using GraphPad Prism version 7.00 software. We estimated a Fisher's exact probability test and the odds-ratio of *L119F-GSTe2* and *CYP6P9a* genotypes (homozygous resistant = RR, heterozygote resistant=RS and homozygous resistant =SS) and

both susceptible (S) and resistant (R) alleles. This allowed us to assess the association between: a) insecticide resistance and mosquito's weight by comparing the proportions of the genotypes of both genes in each group established after weighing; b) blood feeding success and insecticide resistance by comparing the proportion of each genotype in both fed and unfed mosquitoes. After arbitrary regrouping the time into four different intervals with same amplitude, the duration of probing and feeding was analysed with chi-square test by comparing the proportion of *L119F-GSTe2* and *CYP6P9a* genotypes in each defined time interval. After estimating the median of weighted blood meal for each genotype, Kruskal-Wallis and Mann-Whitney tests were used to compare the differences between more than two groups and between two groups, respectively.

Results

Metabolic resistance genes and *An. funestus* mosquito's weight

A total of 1,200 and 273 female mosquitoes were weighted, respectively for field strain (F_1 generation) and lab strain (F_8 generation). The mean weight of a mosquito was 0.9 ± 0.010 mg (minimum = 0.2 mg; maximum = 2.3mg) and 0.89 ± 0.016 mg (minimum = 0.2 mg; maximum = 1.7mg) for field and lab strain respectively. No significant difference was found between the mean weights of two strains. For all the analyses, we arbitrarily grouped mosquitoes according to their weight values, into two different classes as followed: [0 - 1.0] mg and [1.1 - 2.3] mg. Analysis of the distribution of *L119F-GSTe2* mutation genotypes in each class of field strain mosquitoes showed no association between the mosquito's weight and *L119F-GSTe2* genotypes (χ^2 = 0.15; p = 0.9; OR =1.2, 95%CI: 0.3742 - 4.176, for RR *vs* RS; OR=1.1, 95%CI: 0.3659 - 3.606 for RR *vs* SS; OR = 0.9, 95%CI: 0.4943 - 1.709 for RS *vs* SS) (Figure 1 and Table 1). This absence of correlation between the *L119F-GSTe2* genotypes and the weight of

mosquitoes was confirmed at the allele level (OR=1; 95%: CI: 0.5–2.0; *p* =0.5) showing that the L119F mutation may not impact the weight of this *An. funestus* field population (Table 1). In contrast, a significant association was observed between *CYP6P9a* genotypes and the weight of mosquito (χ^2= 29.54, p<0. 0001). Indeed, proportions of RR and RS genotypes were higher than that of SS in the lowest weight class, whereas, for larger weight, mosquitoes with SS genotype were more abundant (67.2%). This association is further supported by odds ratio estimates showing that proportions of homozygote resistant (RR) (OR=5.4; CI 95%: 2.3-12.7; *p*<0.0001) and heterozygote (RS) (OR=5.6; CI 95%: 2.8-11.1; *p*<0.0001) mosquitoes are significantly higher in lowest weight class than the larger one when compared to homozygote susceptible mosquitoes (Table 1). Overall, mosquitoes harbouring the *CYP6P9a*-S susceptible allele displayed higher weight compared to those with the *CYP6P9a*-R resistant allele (OR=2.8; CI 95%: 1.5–5.0; p =0.0003 (Table 1) suggesting that over-expression of the *CYP6P9a* gene is reducing the weight of pyrethroid resistant *An. funestus* mosquitoes.

Table 1: level of association of *L119F-GSTe2* and *CYP6P9a*-R genotypes with mosquito weight by comparing low (0-1.0mg) and high (1-2.4mg) weight samples.

	L119F-GSTe2		***CYP6P9a-R***	
**Genotypes**	**Odds ratio**	***p*-value**	**Odds ratio**	***p*-value**
**RR vs SS**	1.1 (0.4-3.6)	0.5	5.4 (2.3-12.7)	< 0.0001
**RS vs SS**	0.9 (0.5-1.7)	0.4	5.6 (2.8-11.1)	< 0.0001
**RR vs RS**	1.2 (0.4-4.1)	0.5	1.0 (0.5-2.3)	0.5
**S vs R**	1 (0.5-2.0)	0.5	2.8 (1.5-5.0)	0.0003

SS: homozygote susceptible; RR: homozygote resistant; RS: heterozygote; * significant difference p < 0.05.

Impact of *L119F-GSTe2* and *CYP6P9a* mutations on *An. funestus* blood feeding success

***L119F-GSTe2*:** out of the 1,200 individuals from field strain mosquitoes that were allowed to take a blood meal, 457 (39.6%) successfully fed whereas 743 did not. Among blood-fed mosquitoes, a total of 360 were successfully genotyped and 7% (24/360) were homozygote resistant (RR), 28% (103/360) were heterozygotes resistant (RS) and 65% (233/360) were homozygotes susceptible (SS) (Figure 2a). On the other hand, out of the 300 unfed mosquitoes randomly selected for genotyping, 5% (15/300), 32% (62/300) and 63% (189/300), were homozygote resistant, heterozygotes and homozygote susceptible, respectively (Figure 2a). However, the distribution of L119F genotypes was not statistically different between blood-fed and unfed mosquitoes (χ^2=0.63, p=0.7). Furthermore, estimation of odds ratio. (OR=1; CI 95%: 0.5–2.0; p = 0.6) showed overall that mosquitoes bearing the 119F-R resistant allele have the same chance to have a successful blood feeding than those with the 119F-S susceptible alleles (Table 2). This suggests that the ability to take blood is not impacted by the *L119F-GSTe2* mutation in *An. funestus*.

Table 2: Assessment of the association of *L119F-GSTe2* and *CYP6P9a-R* mutations with *An. funestus* mosquito blood feeding

	L119F-GSTe2		***CYP6P9a-R***	
**Genotypes**	**Odds ratio**	***p*-value**	**Odds ratio**	***p*-value**
**RR vs SS**	0.7 (0.2-2.4)	0.4	3.3 (1.4-7.7)	0.01
RS vs SS	1.1 (0.62-2.1)	0.4	1.7 (0.9-3.1)	0.1
RR vs RS	0.6 (0.2-2.3)	0.3	1.9 (0.9-4.4)	0.1
R vs S	1 (0.5-2.0)	0.6	1.8 (1.1-3.2)	0.04

SS: homozygote susceptible; RR: homozygote resistant; RS: heterozygote; * significant difference $p < 0.05$.

CYP6P9a-R: Among a total of 273 mosquitoes that were offered a blood meal 140 successfully fed (51.3%) whereas, 133 did not. Out of the 140 mosquitoes that blood-fed, 134 were successfully genotyped for *CYP6P9a*-R allele revealing that 23% (31/134), 50% (67/134) and 27% (36/134) were homozygote resistant CYP6P9a-RR, heterozygotes CYP6P9a-RS and homozygote susceptible CYP6P9a-SS, respectively (Figure 2b). Among the unfed mosquitoes, 11.3% (15/133) were homozygote resistant CYP6P9a-RR, 47.4% (63/133) heterozygotes and 41.3% (55/133) were homozygote susceptible CYP6P9a-SS. The estimation of odds ratio showed that homozygote resistant CYP6P9a-RR mosquitoes are significantly more able to blood feed than homozygote susceptible (OR = 3.33; CI 95%: 1.4 -7.7; p =0.01). No difference was observed between heterozygote and homozygote resistant CYP6P9a-RR (OR= 1.9, 95%CI: 0.9-4.4; p=0.1) neither with homozygote susceptible CYP6P9a-SS (OR= 1.7, 95%CI: 0.9-3.1; p=0.1) mosquitoes (Table 2). Moreover, it was overall observed that mosquitoes with the *CYP6P9a*-R resistant allele have a greater chance to blood feed than those bearing the susceptible allele (OR = 1.9; CI 95%: 1.03-3.2; p =0.04) (Table 2).

Impact of *L119F-GSTe2* mutation on probing and blood feeding duration

Out of the 120 mosquitoes that were individually filmed to assess the influence of insecticide resistance genes on the probing and feeding duration, 7 (6.14%), 40 (35.08%) and 67 (58.77%) were genotyped as homozygous resistant 119F/F-RR, heterozygotes L119F-RS and hom[illegible]ygotes susceptible L/L119, respectively. Overall, regardless of the genotype, the median value of mosquito's probing duration was 49.5 seconds (minimum = 4s and maximum = 290s). No difference was observed in the probing time of resistant mosquitoes 119F/F-RR (Median = 53 seconds) and heterozygotes L119F-RS (Median = 52s) compared to the homozygote susceptible L/L119 (Median = 52s).

Regarding the blood feeding duration, it was observed that the median and mean time for a mosquito to have a full blood meal was 249.5 seconds and 303 ± 181 seconds respectively, with

a minimum = 68 seconds and a maximum =772 seconds. The feeding duration was longer (median=269s) in L/L119 mosquitoes compared to L119F-RS (229.5s) and 119F/F-RR (214s) but the difference was not statistically significant (p=0.19, Kruskal-Wallis test).

Impact of *L119F-GSTe2* and *CYP6P9a*-R mutations on the blood meal size of *An. funestus*

L119F-GSTe2 : From 457 individuals that took a full blood meal it was observed that the average weighted blood meal of a mosquito regardless of the *L119F-GSTe2* genotype was 3.4 µl/mg (minimum = 1.2 µl/mg; maximum = 9.2 µl/mg). However, the weighted blood meal was not significantly different (P=0.17; Kruskal-Wallis test; Figure 3a) in homozygote susceptible L119-SS (3.0µl/mg) compared to homozygote resistant L119-RR (2.8µl/mg) and heterozygote L119F-RS (3.3µl/mg) mosquitoes. This result suggests that the *L119F-GSTe2* mutation may not affect the volume of blood meal ingested by *An. funestus*.

CYP6P9a-R: The influence of the *CYP6P9a*-R mutation on the volume of blood meal taken by *An. funestus*, was assessed using the 134 blood fed mosquitoes that were successfully genotyped for *CYP6P9a*-R allele. Overall, irrespective of the genotype, the mean weighted blood volume ingested by a mosquito was 4.8 ± 2 µl/mg (minimum = 2 µl/mg; maximum = 13.3µl/mg). However, the weighted blood meal volume of CYP6P9a-SS mosquitoes (Median = 3.71µl/mg) was lower than that of CYP6P9a-RS (Median = 4.73 µl/mg) and of CYP6P9a-RR (Median = 4.78 µl/mg) (Figure 3; p<0.004 for RS vs SS and p<0.006 for RR vs SS, Mann-Whitney test). No difference in the volume of the blood meal was observed between CYP6P9a-RR and CYP6P9a-RS mosquitoes (P=0.7; Mann-Whitney test). This result suggests that the over-expression of *CYP6P9a* gene is associated with an increase of the volume of the blood meal ingested by *An. funestus*.

Expression profile of *AAPP* and *D7* family salivary genes according to *CYP6P9a*-R genotypes

Due to the association observed between the *CYP6P9a*-R genotypes and blood feeding, an attempt was made to assess whether the genotypes of this gene could impact the expression profile of key salivary genes. Analysis of the expression level of AAPP and 4 members of the D7 family salivary genes (D7r1, D7r2, D7r3 and D7r4) did not show a significant difference in expression in homozygote resistant (CYP6P9a-RR) and heterozygote (CYP6P9a-RS) mosquitoes when compared to homozygote susceptible genotype (CYP6P9a-SS) (Figure 4, Table 2) with average fold-change for all these genes lower than 1.5. This result suggests that *CYP6P9a*-R genotypes do not influence the expression profile of both AAPP and D7 family genes in the salivary glands of *An. funestus* mosquitoes.

Discussion.

Due to the absence of markers, the impact of metabolic resistance on life traits of *Anopheles* mosquitoes has been poorly elucidated. Recently, mutations in the *GST* epsilon 2 and in the promoter region of the cytochrome P450 *CYP6P9a*, were described as robust molecular markers for tracking metabolic resistance in pyrethroids resistant populations of *An. funestus* [22,24]. Using these two key markers, this study assess the impact of GST- and P450-based metabolic resistance to pyrethroids on the feeding process and blood meal volume of *An. funestus*.

Impact of metabolic resistance on blood feeding success

The present study revealed that *CYP6P9a* but not the *L119F-GSTe2* mutation could impact the blood feeding success of *An. funestus* mosquito as possessing the *CYP6P9a* resistant allele increased the likelihood of being successful in blood-feeding. Such selective advantage of *CYP6P9a* resistance allele was also previously reported in a semi-field study in experimental

hut trial which observed that homozygous CYP6P9a-RR mosquitoes were significantly more likely to blood feed than susceptible SS [24]. This result suggests that CYP6P9a -mediated metabolic resistance might influence the ability of *An. funestus* mosquito to blood feed. In contrast, the absence of association observed here for the *L119F-GSTe2* mutation needed to be confirmed by further studies as the low sample of L119F-RR homozygote resistance mosquitoes might have biased our analysis. Nevertheless, the mechanism whereby *CYP6P9a*-R resistant allele could impact mosquito feeding is unknown and was not investigated in the present study. One hypothesis to explain this association could be related to the motivation of mosquito to blood feed. In fact, it has been reported that some mosquito individuals that emerged with insufficient teneral reserves require an initial blood meal to compensate for insufficient teneral reserves rather than for egg development during their first gonotrophic cycle [33-35]. This phenomenon is mostly observed in smaller female mosquitoes that emerge with insufficient reserve [2]. Thus, we can presume that *CYP6P9a* resistant mosquitoes which were found significantly smaller than susceptible in the present study were more motivated to blood feed as they were probably the ones requiring more to compensate for their insufficient teneral reserves. Further studies investigating the impact of insecticide resistance on the motivation of *An. funestus* mosquito to blood feed would probably help in confirming this hypothesis.

Impact of metabolic resistance on probing time and feeding duration

The influence of metabolic resistance on probing time and feeding duration was assessed in the present study only for *L119F-GSTe2* mutation. Results revealed no significant impact of this metabolic resistance gene on the time spent by a mosquito to probe. The absence of impact of insecticide resistance on mosquito probing time was also reported for the *knock-down* (*kdr*) resistance gene in *Anopheles gambiae* with no difference in the probing time noticed between genotypes (RR, RS and SS) after exposure to untreated and insecticide-treated net [11]. This

seems to indicate that insecticide resistance might not impact the probing duration of *Anopheles* mosquito during blood feeding. However, this hypothesis must be taken with caution as, to our knowledge, and the exception of the present study as well as the one of Diop et *al*, very little information is available on the impact of insecticide resistance on the probing time during mosquito blood-feeding. In the other hand, even if the difference was not statistically significant, mosquitoes possessing an *119F-GSTe2* resistant allele (both homozygous and heterozygous) spent less time taking their blood meal than susceptible. This corroborate with observation previously made for *kdr* mutation in *An. gambiae* with lower feeding duration for homozygous resistant mosquitoes than heterozygote and homozygous susceptible [11]. The non-significant result observed may be due to the low number of resistant mosquitoes in the present study. However, from the results, it could be hypothesized that *L119F-GSTe2* mutation might confer an advantage to homozygous resistant mosquitoes as it was previously reported that rapid feeding reduces the risk to be killed by the host defensive behaviour [11,36].

Impact of metabolic resistance on blood meal volume

In the present study, we observed that the volume of blood ingested by a mosquito during a single blood feeding was associated with the genotype of the P450 *CYP6P9a* but not with the *L119F-GSTe2*-based metabolic resistance. This suggests that mechanisms involved in metabolic resistance to pyrethroids in *An. funestus* might influence mosquito life-traits differently. However, as already discussed above, we cannot exclude that the absence of the influence observed for *L119F-GSTe2* gene might also be related to the low number of L119F-RR mosquitoes used in the present study. This latter hypothesis seems moreover reinforced by the results of previous studies showing *L119F-GSTe2* mutation [23] and *CYP6P9a*-R resistance gene [37] affecting *An. funestus* fecundity in the same way. The positive association between *CYP6P9a*-R resistant allele and the volume of blood meal is a bit surprising knowing that activity of P450 monoxygenases as well as blood meal digestion, have been reported to generate

an excess production of reactive oxygen species (ROS) increasing oxidative stress which could induce several damages in the mosquito's system that can result to death [38,39]. In fact, because the *CYP6P9a*-R resistant allele was recently reported to be negatively associated with the fecundity of *An. funestus* [37], we were expecting to see *CYP6P9a* resistant mosquitoes taking lower blood meal than susceptible to reduce negative effects of oxidative stress. This suggests that association between the *CYP6P9a*-R resistant allele and mosquito's blood meal size could be an indirect consequence of some other physiological activities. For instance, because *CYP6P9a* resistant mosquitoes were significantly smaller than their susceptible counterparts, and noting that it has been demonstrated that the amount of teneral reserves is proportional to the body size of mosquito [2], we can presume that the high blood meal volume ingested by CYP6P9a-RR mosquitoes might be as a result of a need to compensate for the limited teneral reserves post emergence. In this case, the association observed here could be an indirect consequence of the negative impact of *CYP6P9a*-R resistant allele recently observed on the larval development of *An. funestus* [37] resulting to a small body size, and by consequence to insufficient teneral reserves for resistant mosquitoes. Indeed, it was demonstrated that encountering a nutritional environment by *Anopheles* larvae strongly influences adult fitness-related traits such as body size and teneral metabolic reserves [2,30,40]. However, our finding did not corroborate with the positive association previously reported between the volume of ingested blood meal and mosquito body size [2]. Further studies will help elucidate the underlying reason of this correlation between *CYP6P9a* genotypes and blood meal size.

Influence of CYP6P9a-R resistant allele on salivary gland genes expression

To obtain a successful blood meal, a female mosquito must balance the risk of death caused by host defensive behavior against the benefits to feed on a host species that maximize fertility [41]. Salivary components permit mosquitoes to reduce their engorgement time and increase

their likelihood of survival [5]. In the present study, we assessed the level of expression of genes encoding for some salivary proteins known to be involved on blood intake process of mosquitoes such as, AAPP and members of D7 family proteins [6,42,43]. The comparative analysis of the expression level of these genes between *CYP6P9a* genotypes showed no significant difference between mosquitoes bearing the resistant allele and those with the susceptible one. This result suggests that the expression of AAPP and D7 family salivary genes are not associated to the *CYP6P9a* mutation. This observation is intriguing as some salivary genes such as D7 family genes were previously reported to be over-expressed in resistant *An. funestus* mosquito compared to susceptible strain [22,44-47]. The lack of significance observed with the differential expression of genes in the present study could be explained by the fact that, our analyses in this study were performed on mosquitoes obtained after reciprocal crosses between two different strains and therefore sharing the same background, while other studies compared insecticide resistant field mosquitoes and susceptible laboratory strains. The absence of influence of the *CYP6P9a* gene on the expression level of salivary gland genes involved in the blood feeding process observed in the present study appears to indicate that the association found between this gene and the size of blood meal taken by *An. funestus* mosquito might not be related to the expression of these salivary genes encoding proteins which mediate the blood meal process.

This study revealed that GSTe2-mediated resistance does not affect the blood meal intake of *An. funestus* mosquitoes, whereas *CYP6P9a*-based resistance to pyrethroids is associated with a feeding success and a higher blood meal size. However, this influence on *Anopheles funestus* blood meal intake is not associated with differential expression of major salivary gland proteins involved in blood-feeding. Given the rapid growth of insecticide resistance, it would be interesting to study how this association could impact the fecundity and the vectorial capacity of *An. funestus* mosquitoes.

Author Contributions: E.E.N and C.S.W conceived the study; EEN, L.N, C.N SK and C.S.W designed the study; E.E.N, .L.N, A.B, T.A and M.T carried out the sample collection; L.N, A.B, and T.A reared and maintained the strain in the insectary; E.E.N, L.N A.B, and T.A performed blood feeding experiments. L.N, T.A and M.T performed the molecular analyses; E.E.N, L.N, A.B, M.T and C.N analyzed the data; E.E.N, L.N and C.S.W wrote the manuscript. M.T C.N and S.K reviewed the manuscript. All authors approved the manuscript.

Ethical approval and consent to participate

Ethical clearance was obtained from the National Ethics Committee of Cameroon's Ministry of Public Health (N°2018/04/1000/CE/CNERSH/SP) in conformity to the WMA Declaration of Helsinki. Informed verbal consent was obtained from household owners for using their houses for mosquito collection.

Acknowledgments: We thank Dr Micheal KUSIMO and Mr Francis NKEMNGO; native speakers of English language for accepting to review and edit this paper.

Funding: This study was funded by a Wellcome Trust Training fellowship (109930/Z/15/Z) awarded to ELANGA N'DILLE Emmanuel.

Conflicts of Interest: The authors declare no conflicts of interests.

References

1. WHO (2019) World Malaria Report 2019, Geneva: World Health Organization; 2019. Licence: CC BY-NC-SA 3.0 IGO.
2. Briegel H (1990) Fecundity, metabolism, and body size in *Anopheles* (Diptera: *Culicidae*), vectors of malaria. Journal of medical entomology 27: 839-850.
3. Hurd H (2003) Manipulation of medically important insect vectors by their parasites. Annual review of entomology 48: 141-161.
4. Roitberg BD, Gordon I (2005) Does the *Anopheles* blood meal-fecundity curve, curve? Journal of Vector Ecology 30: 83.
5. Ribeiro J (2000) Blood- feeding in mosquitoes: probing time and salivary gland anti-haemostatic activities in representatives of three genera (*Aedes*, *Anopheles*, *Culex*). Medical and veterinary entomology 14: 142-148.
6. Islam A, Emran TB, Yamamoto DS, Iyori M, Amelia F, et al. (2019) Anopheline antiplatelet protein from mosquito saliva regulates blood feeding behavior. Scientific reports 9: 3129.
7. Thiévent K, Hauser G, Elaian O, Koella JC (2019) The interaction between permethrin exposure and malaria infection affects the host-seeking behaviour of mosquitoes. Malaria journal 18: 79.
8. Taylor P, Hurd H (2001) The influence of host haematocrit on the blood feeding success of *Anopheles stephensi*: implications for enhanced malaria transmission. Parasitology 122: 491-496.
9. Edman JD, Lynn HC (1975) Relationship between blood meal volume and ovarian development in *Culex nigripalpus* (Diptera: *Culicidae*). Entomologia experimentalis et applicata 18: 492-496.
10. Lehane MJ (2005) The biology of blood-sucking in insects: Cambridge University Press.
11. Diop MM, Chandre F, Rossignol M, Porciani A, Chateau M, et al. (2019) Sub-lethal insecticide exposure affects host biting efficiency of Kdr-resistant *Anopheles gambiae*. bioRxiv: 653980.
12. Bhatt S, Weiss D, Cameron E, Bisanzio D, Mappin B, et al. (2015) The effect of malaria control on *Plasmodium falciparum* in Africa between 2000 and 2015. Nature 526: 207.
13. Ranson H, N'guessan R, Lines J, Moiroux N, Nkuni Z, et al. (2011) Pyrethroid resistance in African anopheline mosquitoes: what are the implications for malaria control? Trends in parasitology 27: 91-98.

14. Strode C, Donegan S, Garner P, Enayati AA, Hemingway J (2014) The impact of pyrethroid resistance on the efficacy of insecticide-treated bed nets against African anopheline mosquitoes: systematic review and meta-analysis. PLoS medicine 11.

15. Martinez- Torres D, Chandre F, Williamson M, Darriet F, Berge JB, et al. (1998) Molecular characterization of pyrethroid knockdown resistance (kdr) in the major malaria vector *Anopheles gambiae ss.* Insect molecular biology 7: 179-184.

16. Ranson H, Jensen B, Vulule J, Wang X, Hemingway J, et al. (2000) Identification of a point mutation in the voltage- gated sodium channel gene of Kenyan *Anopheles gambiae* associated with resistance to DDT and pyrethroids. Insect molecular biology 9: 491-497.

17. Berticat C, Boquien G, Raymond M, Chevillon C (2002) Insecticide resistance genes induce a mating competition cost in *Culex pipiens* mosquitoes. Genetics Research 79: 41-47.

18. ROWL[illegible]D M (1991) Behaviour and fitness of γHCH/dieldrin resistant and susceptible female *Anopheles gambiae* and *An. stephensi* mosquitoes in the absence of insecticide. Medical and Veterinary Entomology 5: 193-206.

19. Saingamsook J, Yanola J, Lumjuan N, Walton C, Somboon P (2019) Investigation of Relative Development and Reproductivity Fitness Cost in Three Insecticide-Resistant Strains of Aedes aegypti from Thailand. Insects 10: 265.

20. Kumar K, Sharma AK, Kumar S, Patel S, Sarkar M, et al. (2011) Multiple insecticide resistance/susceptibility status of *Culex quinquefasciatus,* principal vector of bancroftian filariasis from filaria endemic areas of northern India. Asian Pacific journal of tropical medicine 4: 426-429.

21. Martins AJ, Bellinato DF, Peixoto AA, Valle D, Lima JBP (2012) Effect of insecticide resistance on development, longevity and reproduction of field or laboratory selected *Aedes aegypti* populations. PloS one 7: e31889.

22. Riveron JM, Yunta C, Ibrahim SS, Djouaka R, Irving H, et al. (2014) A single mutation in the *GSTe2* gene allows tracking of metabolically based insecticide resistance in a major malaria vector. Genome biology 15: R27.

23. Tchouakui M, Riveron JM, Djonabaye D, Tchapga W, Irving H, et al. (2018) Fitness Costs of the Glutathione S-Transferase Epsilon 2 (*L119F-GSTe2*) Mediated Metabolic Resistance to Insecticides in the Major African Malaria Vector *Anopheles Funestus*. Genes 9: 645.

24. Weedall GD, Mugenzi LM, Menze BD, Tchouakui M, Ibrahim SS, et al. (2019) A cytochrome P450 allele confers pyrethroid resistance on a major African malaria vector,

reducing insecticide-treated bednet efficacy. Science translational medicine 11: eaat7386.

25. Menze BD, Riveron JM, Ibrahim SS, Irving H, Antonio-Nkondjio C, et al. (2016) Multiple insecticide resistance in the malaria vector *Anopheles funestus* from Northern Cameroon is mediated by metabolic resistance alongside potential target site insensitivity mutations. PloS one 11: e0163261.
26. Morgan JC, Irving H, Okedi LM, Steven A, Wondji CS (2010) Pyrethroid resistance in an *Anopheles funestus* population from Uganda. PLoS One 5: e11872.
27. Hunt R, Brooke B, Pillay C, Koekemoer L, Coetzee M (2005) Laboratory selection for and characteristics of pyrethroid resistance in the malaria vector *Anopheles funestus*. Medical and veterinary entomology 19: 271-275.
28. Riveron JM, Irving H, Ndula M, Barnes KG, Ibrahim SS, et al. (2013) Directionally selected cytochrome P450 alleles are driving the spread of pyrethroid resistance in the major malaria vector *Anopheles funestus.* Proceedings of the National Academy of Sciences 110: 252-257.
29. Wondji CS, Irving H, Morgan J, Lobo NF, Collins FH, et al. (2009) Two duplicated P450 genes are associated with pyrethroid resistance in *Anopheles funestus*, a major malaria vector. Genome research 19: 452-459.
30. Briegel H, Lea AO, Klowden MJ (1979) Hemoglobinometry as a method for measuring blood meal sizes of mosquitoes (Diptera: *Culicidae)*. Journal of Medical Entomology 15: 235-238.
31. Livak KJ (1984) Organization and mapping of a sequence on the *Drosophila melanogaster* X and Y chromosomes that is transcribed during spermatogenesis. Genetics 107: 611-634.
32. Koekemoer L, Kamau L, Hunt R, Coetzee M (2002) A cocktail polymerase chain reaction assay to identify members of the *Anopheles funestus* (Diptera: *Culicidae*) group. The American journal of tropical medicine and hygiene 66: 804-811.
33. Klowden MJ, Lea AO (1978) Blood meal size as a factor affecting continued host-seeking by *Aedes aegypti* (L.). The American journal of tropical medicine and hygiene 27: 827-831.
34. Ramasamy M, Srikrishnaraj K, Hadjirin N, Perera S, Ramasamy R (2000) Physiological aspects of multiple blood feeding in the malaria vector *Anopheles tessellatus*. Journal of insect physiology 46: 1051-1059.

35. Takken W, van Loon JJ, Adam W (2001) Inhibition of host-seeking response and olfactory responsiveness in *Anopheles gambiae* following blood feeding. Journal of insect physiology 47: 303-310.
36. Lyimo IN, Haydon DT, Mbina KF, Daraja AA, Mbehela EM, et al. (2012) The fitness of African malaria vectors in the presence and limitation of host behaviour. Malaria journal 11: 425.
37. Tchouakui M, Jacob Riveron M, Mugenzi LMJ, Djonabaye D, Wondji MJ, et al. (2020) Cytochrome P450 metabolic resistance (*CYP6P9a*) to pyrethroids imposes a fitness cost in the major African malaria vector *Anopheles funestus*. Genetics Society.
38. Rivero A, Vézilier J, Weill M, Read AF, Gandon S (2010) Insecticide Control of Vector-Borne Diseases: When Is Insecticide Resistance a Problem? PLoS Pathog 6.
39. Saeaue L, Phumala Morales N, Komalamisra N, Vargas RM (2011) Antioxidative systems defense against oxidative stress induced by blood meal in *Aedes aegypti*. Southeast Asian Journal of Tropical Medicineand Public Health 42: 542.
40. Telang A, Wells MA (2004) The effect of larval and adult nutrition on successful autogenous egg production by a mosquito. Journal of Insect Physiology 50: 677-685.
41. Edman JD, Scott TW (1987) Host defensive behaviour and the feeding success of mosquitoes. International Journal of Tropical Insect Science 8: 617-622.
42. Calvo E, Dao A, Pham VM, Ribeiro JMC (2007) An insight into the sialome of *Anopheles funestus* reveals an emerging pattern in anopheline salivary protein families. Insect Biochem Mol Biol 37: 164-175.
43. Calvo E, Mans BJ, Andersen JF, Ribeiro JM (2006) Function and evolution of a mosquito salivary protein family. J Biol Chem 281.
44. Elanga-Ndille E, Nouage L, Binyang A, Assatse T, Tene-Fossog B, et al. (2019) Overexpression of two members of D7 salivary genes family is associated with pyrethroid resistance in the malaria vector *Anopheles funestus ss* but not in *Anopheles gambiae* in Cameroon. Genes 10: 211.
45. Riveron JM, Ibrahim SS, Chanda E, Mzilahowa T, Cuamba N, et al. (2014) The highly polymorphic *CYP6M7* cytochrome P450 gene partners with the directionally selected *CYP6P9a* and *CYP6P9b* genes to expand the pyrethroid resistance front in the malaria vector *Anopheles funestus* in Africa. BMC genomics 15: 817.
46. Samb B, Konate L, Irving H, Riveron JM, Dia I, et al. (2016) Investigating molecular basis of lambda-cyhalothrin resistance in an *Anopheles funestus* population from Senegal. Parasites & vectors 9: 449.

47. Wilding C, Weetman D, Rippon E, Steen K, Mawejje H, et al. (2015) Parallel evolution or purifying selection, not introgression, explains similarity in the pyrethroid detoxification linked *GSTE4* of *Anopheles gambiae* and *An. arabiensis*. Molecular genetics and genomics 290: 201-215.

Figures legend

Figure 1: Distribution of genotypes of *L119F-GSTe2* (A) and *CYP6P9a*-R (B) markers according to *An. funestus* mosquito weight

Figure 2: Distribution of *L119F-GSTe2* (A) and *CYP6P9a-R* (B) genotypes between blood-fed and unfed *An. funestus* mosquitoes.

Figure 3: Blood meal size of *An. funestus* mosquitoes according to their *L119F-GSTe2* (A) and *CYP6P9a*-R (B) genotypes.

Figure 4: Expression level of AAPP and some members of D7 family genes in CYP6P9a-RR and CYP6P9a-RS mosquitoes in comparison with *CYP6P9a* susceptible mosquitoes. The dotted line indicates genes expression level in *CYP6P9a* susceptible mosquitoes used as standard.

Figure 1

Click here to access/download;Figure;Fig_1.tif

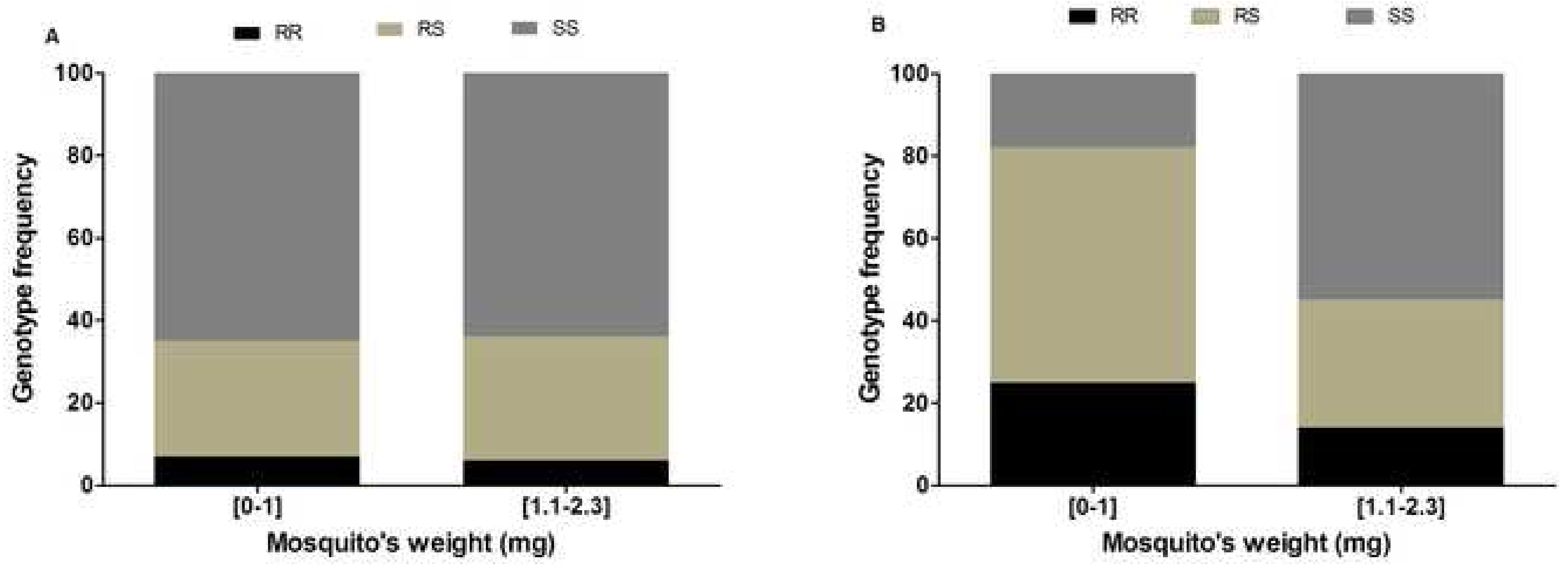

Figure 2

Click here to access/download;Figure;Fig_2.tif

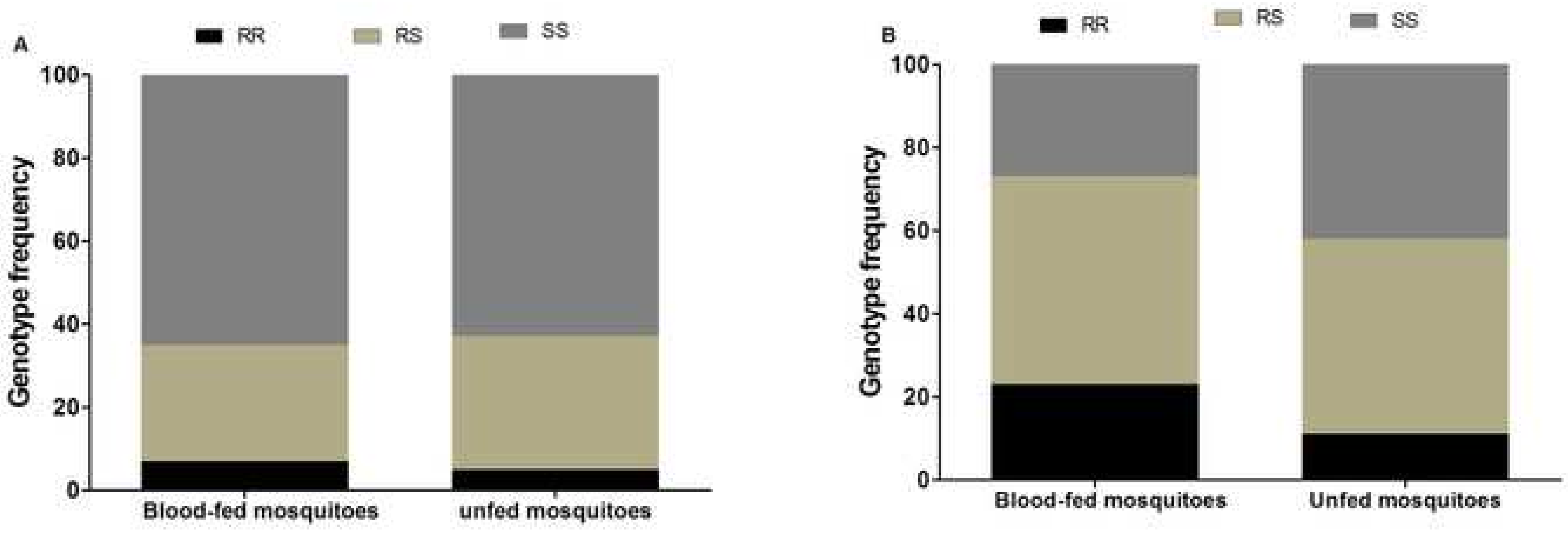

Figure 3

Click here to access/download;Figure;Fig_3.tif

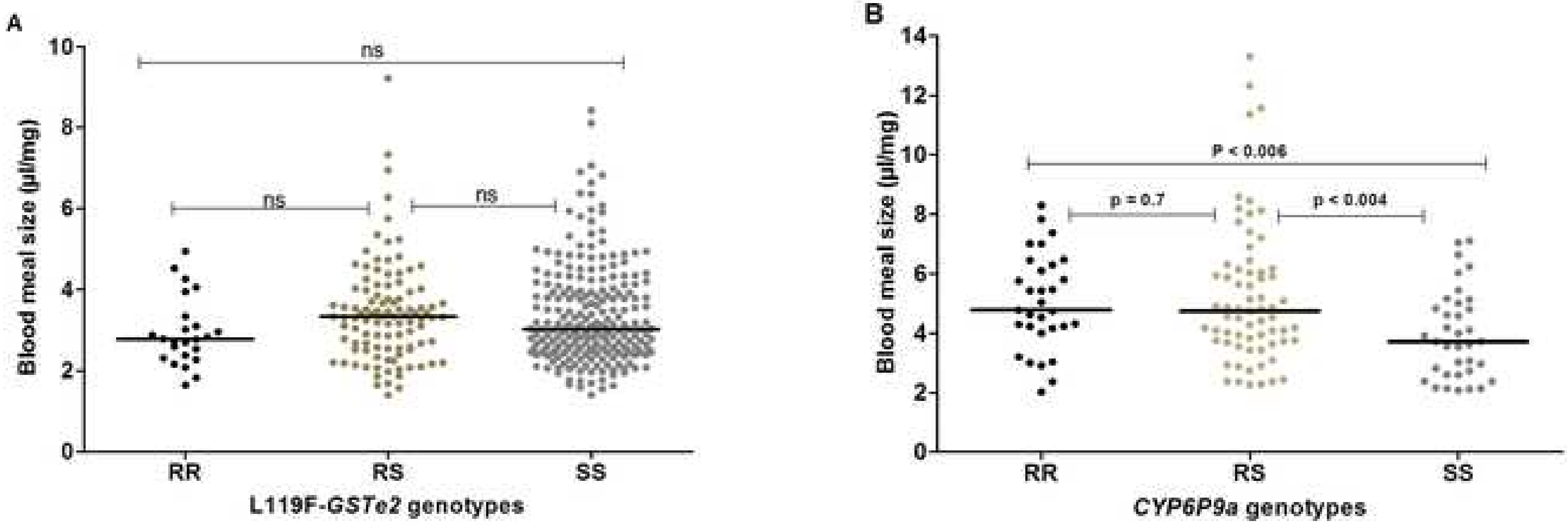

Click here to access/download;Figure;Fig_4.tif

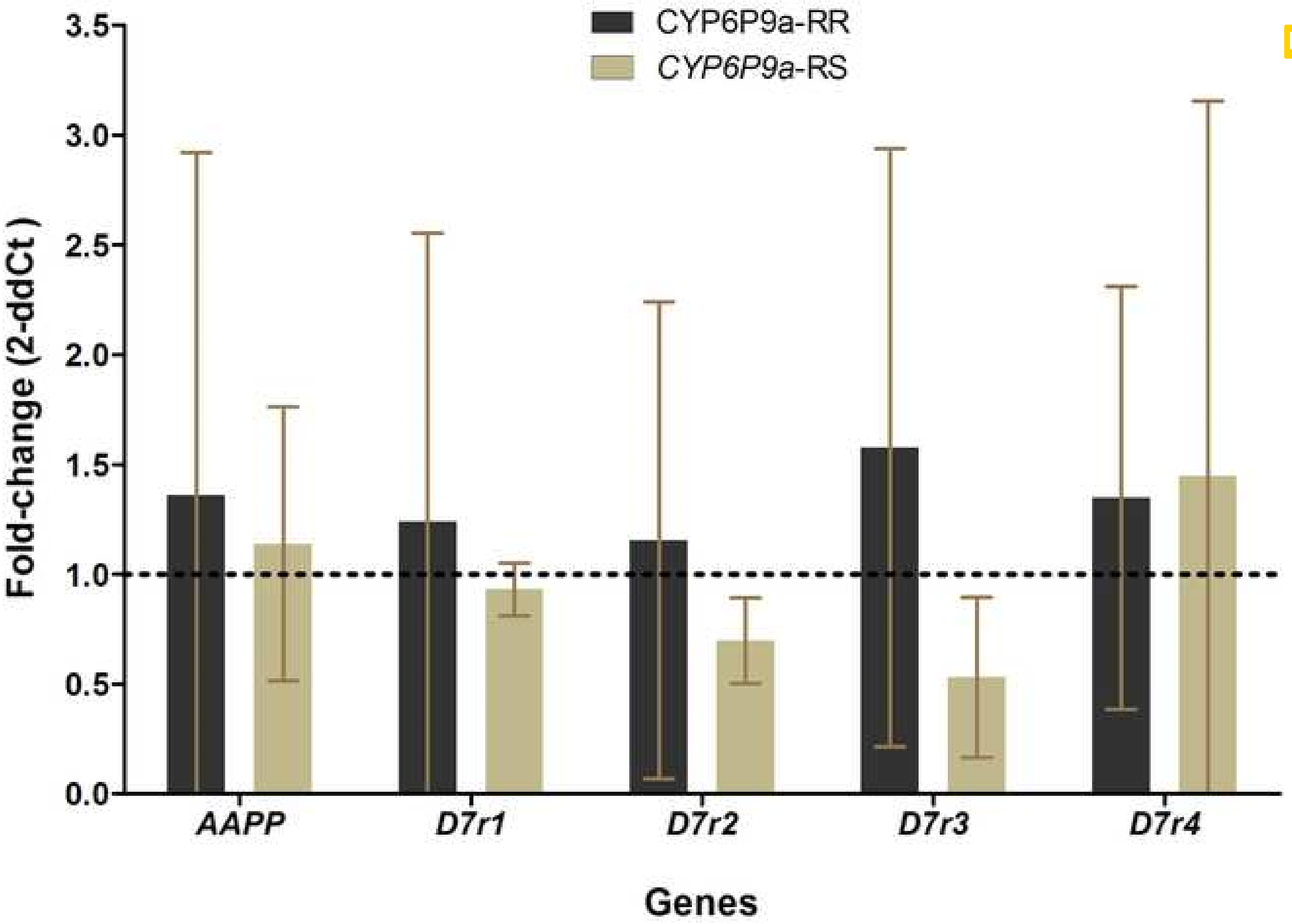

Click here to access/download
Preprint PDF
Nouage et al_Manuscript .pdf
