# Supplementary material for: Influence of GST- and P450-based metabolic resistance to pyrethroids on blood feeding in the major African malaria vector Anopheles funestus
Source: PLoS One. 2020 Sep 18;15(9):e0230984. doi: 10.1371/journal.pone.0230984 (PMC7500606; doi:10.1371/journal.pone.0230984)
Supplement: Attachment — Submitted filename: PONE-D-20-07208_R1.pdf [file pone.0230984.s004.pdf]

PLOS ONE

Influence of GST- and P450-based metabolic resistance to pyrethroids on blood feeding in the major African malaria vector Anopheles funestus

--Manuscript Draft--

**Manuscript Number:**	PONE-D-20-07208R1
**Article Type:**	Research Article
**Full Title:**	Influence of GST- and P450-based metabolic resistance to pyrethroids on blood feeding in the major African malaria vector Anopheles funestus
**Short Title:**	Bloodfeeding process and insecticide resistance in Anopheles funestus mosquito
**Corresponding Author:**	Lynda DJOUNKWA NOUAGE, Msc<br>Centre for research in Infectious diseases (CRID)<br>Yaounde, CAMEROON
**Keywords:**	Malaria, Mosquito, Anopheles funestus, Insecticide resistance, Metabolic resistance, GST, P450, Blood meal intake
**Abstract:**	Insecticide resistance genes are often associated with pleiotropic effects on various mosquito life-history traits. However, very little information is available on the impact of insecticide resistance on blood feeding process in mosquitoes. Here, using two recently detected DNA-based metabolic markers in the major malaria vector, An. funestus, we investigated how metabolic resistance genes could affect the blood meal intake.After allowing both the field F1 and lab F8 Anopheles funestus strains to feed on the human arm for 30 minutes, we assessed the association between key parameters of blood meal process including, probing time, feeding duration, blood feeding success, blood meal size, and markers of glutathione S-transferase (L119F-GSTe2) and cytochrome P450 (CYP6P9a_R) - mediated metabolic resistance. None of the parameters of blood meal process was associated with L119F-GSTe2 genotypes. By contrast, for CYP6P9a _R, homozygous resistant mosquitoes were significantly more able to blood-feed than homozygous susceptible (OR = 3.3; CI 95%: 1.4-7.7; P =0.01) mosquitoes. Moreover, the volume of blood meal ingested by CYP6P9a-SS mosquitoes was lower than that of CYP6P9a-RS (P<0.004) and of CYP6P9a-RR (P<0.006). This suggests that CYP6P9a geneis linked with the feeding success and blood meal size of An. funestus. However, no correlation was found in the expression of CYP6P9a and that of genes encoding for salivary proteins involved in blood meal process.<br>This study suggests that P450-based metabolic resistance may influence the blood feeding process of Anopheles funestus mosquito and consequencetly its ability to transmit malaria parasites.
**Order of Authors:**	Lynda NOUAGE, Msc
	Emmanuel ELANGA-NDILLE
	Achille Binyang
	Magellan Tchouakui
	Tatiane ATSATSE
	Cyrille Ndo
	Sévilor KEKEUNOU
	Charles S. Wondji
**Response to Reviewers:**	Editor’s comments<br>1. Please ensure that your manuscript meets PLOS ONE's style requirements, including those for file naming. The PLOS ONE style templates can be found at<br>https://journals.plos.org/plosone/s/file?id=wjVg/PLOSOne_formatting_sample_main_body.pdf and<br>https://journals.plos.org/plosone/s/file?id=ba62/PLOSOne_formatting_sample_title_authors_affiliations.pdf.<br>Thank you for this remark, the manuscript have been check completely for any

Powered by Editorial Manager® and ProduXion Manager® from Aries Systems Corporation

formatting mistake.
2. We noticed you have some minor occurrence of overlapping text with the following previous publication(s), which needs to be addressed:
https://journals.plos.org/plosone/article?id=10.1371%2Fjournal.pone.0103816
https://www.mdpi.com/2075-4450/10/9/265/htm
Answer: This observation was taken into account and we insured to strongly eliminated the overlapping even if we didn't know exactly which part of the text is overlapping
In your revision ensure you cite all your sources (including your own works), and quote or rephrase any duplicated text outside the methods section. Further consideration is dependent on these concerns being addressed.
Answer: Thank you so much for highlighting this. We have rephrased all the duplicated text detected and referenced the remaining parts as you can see in the revised version
3. In your Methods, please describe exactly how volunteers were recruited to provide blood meals in your study.
Answer: Thank you for this observation. This point was taken into account and a sentence describing briefly how volunteers were recruited to provide blood meal has been added in the revised manuscript (see lines 146 To 149).
4. PLOS requires an ORCID iD for the corresponding author in Editorial Manager on papers submitted after December 6th, 2016. Please ensure that you have an ORCID iD and that it is validated in Editorial Manager. To do this, go to 'Update my Information' (in the upper left-hand corner of the main menu), and click on the Fetch/Validate link next to the ORCID field. This will take you to the ORCID site and allow you to create a new iD or authenticate a pre-existing iD in Editorial Manager. Please see the following video for instructions on linking an ORCID iD to your Editorial Manager account: https://www.youtube.com/watch?v=_xcclfuvtxQ
Answer: Thank you so much for mentioning this. I created an ORCID iD which allowed to submit the revised version of the manuscript
5. Thank you for stating the following in the Acknowledgments Section of your manuscript:
'This study was funded by a Wellcome Trust Training fellowship (109930/Z/15/Z) awarded to ELANGA N'DILLE Emmanuel.
We note that you have provided funding information that is not currently declared in your Funding Statement. However, funding information should not appear in the Acknowledgments section or other areas of your manuscript. We will only publish funding information present in the Funding Statement section of the online submission form.
Please remove any funding-related text from the manuscript and let us know how you would like to update your Funding Statement. Currently, your Funding Statement reads as follows:
'The funders had no role in study design, data collection and analysis, decision to publish, or preparation of the manuscript'
Answer: This observation was taking in consideration and the funding-related text have been removed from the manuscript.
Reviewer #1: It is a technically well executed study and the manuscript well written and presented.
The subject is interesting. I would request that the authors please consider a few points in their revised version:
1.In general, I think "impact" of CYP6P9a-R genotypes on several blood feeding behavior parametres is too strong. Please consider replacing impact (causal factor?) with "possible association" or similar alternative term
For example, Line 358: "Due to the association observed between the CYP6P9a-R genotypes and blood feeding, an attempt was made to assess whether the genotypes of this gene could impact the expression profile of key salivary genes" and 451 influence of CYP6P9a-R resistant allele on salivary gland genes expression
I am not sure what is meant, how would the CYP6P9a-R IMPACT or Influence the expression of salivary glands? (Please consider alternative "possible association" term.)
Answer: We thank the reviewer for this comment. We agree with him that talking about 'impact'' in our study could be too strong to characterise our observations. According to its recommendation, we have replaced the term ''impact'' by '' possible association or effect "in the entire revised manuscript.
2. Assuming volume of blood meal ingested by CYP6P9a-R mosquitoes was (sligthly) lower than that of CYP6P9a-S, does that mean vectorial capacity is also affected? Please justify the link, or tone down the statement

Line 45 “This study suggests that P450-based metabolic resistance may increase the blood feeding ability of malaria vectors and potentially impacting their vectorial capacity” contradicts with line 477 “interesting to study how this association could impact the fecundity and the vectorial capacity of An. funestus mosquitoes”
Answer: We thank the reviewer for this remark. Does assumption that volume of blood meal ingested by CYP6P9a-R mosquitoes was (slightly) lower than that of CYP6P9a-S means that vectorial capacity is also affected? That is an interesting question to be investigated. At this stage, since we did not evaluate this aspect, we are just making hypothesis that association between the CYP6P9a-R allele and the volume of blood meal ingested may potentially influence the vectorial capacity. Indeed, since the vectorial capacity is a concept analogous which is a function of the vector's density in relation to its vertebrate host, it could be influenced by the variations in mosquito’s density in one area. So, given that mosquitoes’density hardly depends to mosquito fecundity and that the latter is itself significantly associated with the volume of blood meal ingested, we are hypothesising that, lower volume of blood meal ingested by CYP6P9a-R mosquitoes could affect its fecundity by reducing the number of eggs they lay. The reduction of the number of eggs laid would led to the decrease of the density of CYP6P9a-R mosquitoes in a given population and therefore to a reduction of vectorial capacity of these mosquitoes. However, since we did not assess this impact on the vectorial capacity and because vectorial capacity depends also to other parameters such as infectiousness, the longevity and the behaviour our statement remains one hypothesis and that is why in line 477, we are saying that it would be interesting to investigate this hypothesis through further studies. However, to avoid any confusion for the understanding of our statement, we rewrite the sentence at line 45 in the revised manuscript as followed: “This study suggests that P450-based metabolic resistance may influence the blood feeding process of Anopheles funestus mosquito and by consequence its ability to transmit malaria vectors and parasites.”
3. The data presented in Figure 4 are not clear what it is, and the variation shown (SEM? SD??) is not normal (or not well analysed and explained). If true, alternative ways could be considered to present this data.
Answer: We thanks the reviewer for this comment and we are totally agree with him that there is sometime wrong with this graphic. Indeed, since fold changes are not normally distributed data, there is no reason to represent them with standard errors. Moreover, these standards errors were not well estimated. After reanalysing these data we found that relative expression of each gene is the great way to present this result. Thus, since the trend of the result is the same, in the revised manuscript we replace the former figure 4 by a new one presenting the comparative relative expression of each between CYP6P9a-RR, CYP6P9a-RS and CYP6P9a-SS mosquitoes. Also, some few modifications were made in the part entitled “Expression profile of AAPP and D7 family salivary genes according to CYP6P9a-R genotypes” of the results section of the revised manuscript.

Reviewer #2:
Although the authors have done work that is reasonably methodologically sound, I am not fully convinced by the conclusions, rather than having a problem with the findings. The key problem is that the argument of the association of the genotype with behaviours is not strong enough. I will outline some of the key issues that need to be addressed to strengthen the argument enough to be published. The experiments are largely sound, but there needs to be a large scale restructuring of the discussion and the conclusions drawn, as the arguments are not strongly convincing.
Major comments:
There is a substantial body of work about the effects of metabolic resistance and life history. The novel factor of your work is that your work involved wild specimens, where previous studies on metabolic resistance and its effects were on laboratory strains where the resistance mechanisms were characterised. Although the metabolic screening tools are useful, this does not mean that studies on metabolic resistance could not happen before it. The success rate of genotyping of the wild specimens (78% success rate) is concerning. The substantial amount of unsuccessfully genotyped individuals could have altered the findings. This must be acknowledged.
Answer: We agree with the reviewer that our study is not the first one on metabolic resistance since some other studies were already done. However our study is among the most recent ones using a DNA based marker to investigate the physiological impact of metabolic resistance in Anopheles mosquitoes. Concerning the L119F-

GSTe2 genotyping, we agree with the reviewer that the number of unsuccessfully genotyped individuals is not negligible and could have altered our findings for this marker. This aspect was taken into account as it could be the source of low number of L119F-GSTe2-RR mosquitoes in this study. That is why in the first manuscript we have indicated that our findings for the L119F-GSTe2 mutation could have been biased by the low number of L119F-GSTe2-RR mosquitoes. However to be more understandable we added more comments in the revised manuscript (see lines 410 to 414). Nevertheless, since our analyses were performed on approximately 78% (360/457) of our total sample we think that this sample size was more than enough for this assessment.

The authors need to guide the readers a little more. There are times in the methods section where it is confusing as to why the methods were being employed, but this was clarified in the results section. It would make the MS easier to read if the reason for the methodology was introduced prior to the description of the method.

Answer: We thanks the reviewer for this suggestion. This remark have been taken into consideration and the methods section were rewritten in the revised manuscript to help the readers understanding why each method was employed in the study.

During the PCR methods section, it seems that the different sections were written by different people. In some parts it is satisfactory, and in others, it seems that the methods come from a dissertation. PCR methods in a manuscript include concentrations and not volumes, and these sections are more suited for a standard operating procedure than a manuscript. Centrifugation data must be reported in rcf (g) not rpm. Please ensure that the language of the molecular methods is appropriate.

Answer: All these remarks were taken into consideration. We have revised the PCR section in the revised manuscript and we replaced the volume of the reagents by the concentrations. We also converted the centrifugation data from rpm to rcf.

The weight of the An. funestus mosquitoes you describe is very high. In my experience mosquitoes weighing close to a milligram normally have wingspans of between 3.5 to 4mm, and these are very large An. gambiae. This result is therefore quite startling. It also leads to questions about the conclusions about genotype and size.

Answer: The observation of the review about the size of the weight of mosquitoes used in this study is understandable, since these mosquitoes look bigger than what usually reported. However, we think that there are important points to consider here before comparing the weight of our mosquitoes and those from previous studies. These points could certainly explained the huge differences between our study and other ones. For instance, instead of weighing dead and dried mosquitoes as commonly done in previous studies, mosquitoes used in our study were not killed and dried before being weighted. So mosquitoes' weight in our study is not a dried weight. This difference in the method used is important since there are numerous components that can influence mosquito's weight which are eliminated when mosquito is drying. Additionally, in our study we worked with mosquitoes of 3 to 7 days old whereas other previous studies mainly worked with mosquitoes of 1 day old maximum. This difference in age can also lead to the difference of the weight between two mosquitoes. One other important element that could explain the bigger weight of mosquitoes used in this study compared to other ones, is the fact that unlike of what done in previous studies, mosquitoes here were fed with sugar solution before being weighting after being starved during 24h. Overall, all these parameter could explained why our mosquitoes look bigger than usual. Furthermore, is important to noted that the weight our mosquitoes is not so different to what Roitberg and its colleagues obtained by weighting also fresh mosquitoes (Roitberg BD, Mondor EB, Tyerman JGA (2003) Pouncing spider, flying mosquito: blood acquisition increases predation risk in mosquitoes. Behavioral Ecology, Vol. 14 No. 5: 736–740, DOI: 10.1093/beheco/arg055). However, it could be interesting in further studies to assess the weight of Anopheles funestus mosquito when he is dried.

Furthermore, it is also concerning that you talk about size, and the relationship between genotype and size, without describing how you controlled for the larval diet. If this was not controlled for, then genotype may be related to larval feeding propensity that resulted in larger adults, which is a slightly different situation. It may also simply be that there was feeding variation between cohorts. Therefore, if you cannot describe how larval feeding was standardised, then conclusions about size cannot be made.

Answer: We completely agree with the reviewer that the mosquito's weight hardly depend of larval rearing and feeding condition. Larval diet strongly impact the weight of mosquitoes. But in the case of our study, the potential bias due to the larval rearing condition could not impact our findings. Indeed, at the larval stage, mosquitoes were

Powered by Editorial Manager® and ProduXion Manager® from Aries Systems Corporation

reared and fed in the same conditions since eggs laid by parent were put in the same trays regardless the genotypes. It's only at the adult stage and after the weighting and the blood-feeding that mosquitoes were genotyped and separated according to their genotypes. Before the genotyping mosquitoes at all stages were pooled and reared in the same conditions. This is one of the main advantage of using the molecular markers for such assessment since they help to reduce all the confounding factors which could impact the outcome of the study.
Something that also jumps out is that CYP6P9-SS adults are bigger. This is quite unusual, as larger mosquitoes tend to be more tolerant of insecticides, or that resistant phenotypes are associated with larger body sizes (eg: Osuwu et al: Sci Rep. 2017 Jun 16;7(1):3667, Jeanrenaud et al, PLoS One. 2019 Apr 18;14(4):e0215552). Such an unusual result must be discussed.
Answer: Thanks for this remark. We were also surprised by this result since we were expecting to observe the opposite pattern according to the fact that resistant phenotypes are often reported associated with larger body sizes. As suggested by the reviewer, this point is now discussed in the revised manuscript (See lines: 422-437).
Line 375-396: Impact of metabolic resistance on blood feeding success
This particular argument does not hold water. Firstly, you have just given the definition of an anautogenous mosquito. Secondly, you hypothesize that CYP6P9 could affect feeding because of teneral reserves. You have not examined this, and crucially, as have described above, you do not describe how feeding was controlled for and this is crucial for comments on size. Also, as I have described above, the mosquitoes you have described are extremely large for An. funestus, so I don't think poor teneral reserves can account for the findings you have described. Related to this, later there are discussions about resistance and blood feeding, hypothesising that the smaller resistant mosquito (which is unusual in itself) needs to take more blood because they have lower teneral reserves. This line of argument is not well supported as there was no evidence that food quantity was controlled, and therefore there cannot be arguments about teneral reserves. Teneral reserves are not only represented by size, and if there is an argument to made about this, there are simple calorimetric assays described in the MR4 manual to measure this. Finally, the fact that these mosquitoes seem to be very large for their species, it seems problematic to ascribe this behaviour to poor teneral reserves.
Answer: We well understand the concern highlighted by the reviewer. However, as we mentioned above, we have eliminated all potential bias related to feeding and rearing conditions since our mosquitoes were genotyped and sorted according their genotype only after blood-feeding experiments mosquitoes used in this study were reared in the same conditions. Indeed, larvae were pooled in the same tray, fed in the same conditions. After emergence, adult mosquitoes were in the same cages and were fed with the same sugar solution. It is only after all the experiments and genotyping that the link between body size and the genotype was assessed. However, although we have reduced the bias related to breeding conditions, it cannot be excluded that the weight of mosquitoes was influenced by the digestion of the sugar solution which could have been incomplete in some individuals. Other studies based on calorimetric analyses could probably allow a better comparison of the quantity of teneral reserves between resistant and susceptible mosquitoes. This aspect was pointed out and discussed in the revised manuscript.
Line 398-417 Impact of metabolic resistance on probing time and feeding duration
Besides the comment that I have made on the document that it is not worth discussing results that are not statistically significant, there is another methodical flaw that is revealed in this discussion. By the authors' admission, there were not a large amount of resistant heterozygotes. Although crosses were performed to generate heterozygotes, if Fumoz was available for crossing, surely uncrossed Fumoz would have been a useful source of RR mosquitoes? Is there a reason why RR and SS individuals were not obtained for study from the original strains?
Answer: We thank the reviewer for the comment. Since our study aimed to assess the impact of metabolic resistance in important feeding parameters of Anopheles funestus mosquito, it was crucial to work with mosquitoes shearing the same genetic background. For this purpose we worked with Fumoz (full resistance) and FANG (full susceptible) strains. Since the genetic background of these two lab strain is huge different, we thought that it would not have been reasonable to work separately with RR (from Furmoz) and SS (from FANG). So to limit the impact of this genetic background differences the option chosen and which is recommended (Thiago Affonso Belinato and Ademir Jesus Martins (March 2nd 2016). Insecticide Resistance and

Fitness Cost, Insecticides Resistance, Stanislav Trdan, IntechOpen, DOI: 10.5772/61826. Available from: https://www.intechopen.com/books/insecticides-resistance/insecticide-resistance-and-fitness-cost), was to work with mosquitoes generated after crossing FANG and Fumoz. Working with individuals generated from crosses avoid our funding being influenced by other several genetic difference.
Line 418-450: Impact of metabolic resistance on blood meal volume
There are some issues in this section.
Line 427-431. The authors describe that "the positive association CYP6P9a-R resistant allele and the volume of blood meal is a bit surprising knowing that activity of P450 monoxygenases as well as blood meal digestion, have been reported to generate an excess production of reactive oxygen species (ROS) increasing oxidative stress which could induce several damages in the mosquito's system that can result to death [38,39]. This is not really that surprising if you consider that the resistant An. funestus strains Fumoz and in particular Fumoz R has a marked capacity to cope with oxidative stress, both phenotypically and due to increased gluthatione peroxidase and catalase activity (Oliver and Brooke PLoS One. 2016 Mar 10; 11(3):e0151049): Your reference cites information about oxidative stress in Aedes. There are better references, as there is work on oxidative stress in Anopheles, not only the previously mentioned which shows that oxidative stress in An. funestus specifically is quite different to Aedes. Champion and Xu (Sci Rep 8, 13054 (2018)) show the complexity of the interaction between oxidative stress, insecticide resistance and fecundity in An. gambiae. Crucially, what they show a relationship between oxidative stress and resistance, with resistant An. gambiae also has a greater oxidative stress capacity. Over and above this, epsilon class GSTs are associated with oxidative stress defence. In the selections, did these genotypes segregate separately (was this checked), because it will be difficult to discuss oxidative stress in relation to CYP6P9 (where P450s do increase oxidative stress) if the effect of the GSTe2 in the same individual has not been considered.
Answer: The point highlighted by the reviewer is very interesting and was taken into consideration. We really found interesting all papers mentioned by the reviewer, since they help us improving our discussion section in the revised manuscript. Nevertheless, We would like to precise that the effect of GSTe2 and P450s where not assessed on the same mosquitoes since the GSTe2, unlike to the duplicated CYP6P9a and b, is not implicated in resistance in FUMOZ strain.That is the reason why we used two different strains to assess the impact of metabolic resistance in An. funestus blood-feeding process: i) FUMOZ lab strain to assess the impact of CYP6P9a and ii) for the impact of GSTe2 we used the field collected mosquitoes from Cameroon where this gene where previously shown to be overexpressed in DDT and Permethrin resistant mosquitoes (Riveron JM, Yunta C, Ibrahim SS, Djouaka R, Irving H, et al. Genome Biology (2014)). Furthermore, we agree with the reviewer that it will be difficult to discuss oxidative stress in relation to CYP6P9 if the effect of the GSTe2 in the same individual has not been considered, since both enzyme have opposite effect. In our study we did not assess this point because as said above, among the two genes concerned in our study, only the CYP6P9a is reported over-expressed and evolved in insecticide resistance of FUMOZ-R strain. However, although GSTe2 is not over-expressed and evolved in insecticide resistance in Fumoz strain, I would have been interesting to measure its activity in CYP6P9a-RR mosquitoes before and after blood-feeding. This would have been more informative about the mechanism used by the CYP6P9a-RR mosquito to cope with the oxidative stress generated by the positive association between high blood meal volume and the CYP6P9a-R mutation. In the same vein, the assessment of the activity of other antioxidant agents would be helpful for the great understanding of the control of oxidative damage by CYP6P9a-RR mosquito after blood-feeding. All these points are now included in the discussion section in the revised manuscript.
Another major issue that I have with this argument has to do with the argument about fitness costs in An. funestus. Although your reference for this is a manuscript where fitness costs are noted, this manuscript examined it in crosses, as you have done in this experiment. However, that study and the present study ignore the work of Okoye et al (Bull Entomol Res. 2007 Dec; 97(6):599-605), which did not find a fitness cost to resistance in Fumoz. Their conclusion is supported by the fact that Fumoz-R maintains high deltamethrin resistance intensity without selection. Therefore, a fitness cost cannot be unambiguously assigned to the resistant funestus.
Answer: First of all, we would like to reassure the reviewer that our study did not ignore the work of Okoye et al. We did not referred to this previous work because the authors

used two strains of different genetic background to evaluate the fitness associated with resistance in Fumoz since they do not have a DNA-based marker. The main approach for such assessment is to cross the resistant and the susceptible strains so that the fitness cost will be evaluated on mosquitoes with the same genetic background. This is the approach used in this study and previously by Tchouakui et al 2020. However, since our findings appeared to show no reduction of the fitness of CYP6P9a-RR mosquitoes as previously observed by Okoye et al in phenotypically resistant FUMOZ strain, we have revised and restructured our discussion. In the revised manuscript we are now taking into account the fact that our findings seem to suggest that the CYP6P9a-based metabolic resistance might not probably compromise some life straits of Anopheles funestus mosquito, including its ability to have a blood meal and the volume of blood it is able to ingest. Because of these modification of the discussion of our results, the paper of Okoye et al, is now cited in the revised manuscript.

The studies on salivary gene expression are problematic. I assumed that there would not be a different as the error bars (if they are error bars and not standard deviation) are huge. This is concerning and this is not a good quality result for publication. Is there an explanation for this?
Answer: We thanks the reviewer for this comment. As already said above, there was sometime wrong with this graphic. Indeed, since fold changes are not normally distributed data, there is no reason to represent them with standard errors. Moreover, these standards errors were not well estimated. After reanalysing these data we found that relative expression of each gene is the great way to present this result. Thus, since the trend of the result is the same, in the revised manuscript we replace the former figure 4 by a new one presenting the comparative relative expression of each between CYP6P9a-RR, CYP6P9a-RS and CYP6P9a-SS mosquitoes. Also, some few modifications were made in the part entitled “Expression profile of AAPP and D7 family salivary genes according to CYP6P9a-R genotypes” of the results section of the revised manuscript
Minor comments
Throughout the document: change homo or heterozygote to heterozygous where it is used as an adjective rather than a noun.
Thanks to the reviewer, homo or heterozygote has been changed to heterozygous thought out the manuscript
There are numerous small comments annotated on the manuscript that must be addressed.
Thanks a lot for all the attention on the manuscript all the comments have been addressed as you can see in the revised version
How was RNA quality assessed?
Answer: This was mainly evaluated based on the ratio obtained from Nanodrop with ratio ranged from 1.8 - 2.0. Using the bioanalyzer could have been the best option for that, but; unfortunately this expensive equipment is not yet available in our lab.
I do not think that actin was the best choice of housekeeping gene for a study about a blood feeding response. Actin would not stay stable during blood feeding as the abdominal expansion associated with blood feeding would alter actin expression.
Answer: Actin has been used for many studies on the expression profiling of insecticide resistance genes in An. funestus. Probably, the abdominal expansion associated with blood feeding can alter actin expression as mentioned the reviewer but in our study we assessed genes expression in the salivary glands. Nevertheless this has to be taken into account for further studies on this aspect.
Line 291: This is quite unusual. The laboratory strains would have fed quite well if they were allowed to feed for 30 minutes. How old were these mosquitoes when they were fed? Over and above the parameters I described before, it may be that the mosquitoes did not feed well, as 3 days is not the optimal feeding age for An. funestus.
Answer: The mosquitoes used for the experiments aged between 3-7 days old. Probably 3 days is not the optimal feeding age for An. funestus but all the mosquitoes did not have 3 days. Also this cannot impact the outcome of the association between the markers and the feeding since the age cannot affect only one of the three genotypes given that mosquitoes were reared, fed and kept together.
Did the mean amount of blood taken in the GST genotype differ significantly from the CYP6 genotype?
Yes a significant difference was found between GST and CYP6, with mosquitoes displaying low mean blood meal volume for GSTs ($P<0.0001$). However, since our aims was to compare these two mosquitos’ strains, we did not include this analysis in

	our manuscript. Also, we thought that this analyse might have been biased by the fact that one type of mosquito was lab strain (CYP6P9a-mosquitoes) which is most able to take it blood meal in lab condition than field collected (GSTe2 mosquitoes) field.<br>Line 368: This is not true. There is a body of work that describes the interplay between metabolic resistance and longevity and stress response, among others. It is not as well examined as target site resistance, but there is a body of work on the subject. If the statement was amended to say that this is so for wild specimens, that is true because the studies were primarily studied in lab strains where the metabolic resistance profile had been defined.<br>This comment was take into consideration and Line 368 was removed in the revised manuscript
Additional Information:	
Question	Response
**Financial Disclosure**<br><br>Enter a financial disclosure statement that describes the sources of funding for the work included in this submission. Review the submission guidelines for detailed requirements. View published research articles from *PLOS ONE* for specific examples.<br><br>This statement is required for submission and **will appear in the published article** if the submission is accepted. Please make sure it is accurate.<br><br>**Unfunded studies**<br>Enter: *The author(s) received no specific funding for this work.*<br><br>**Funded studies**<br>Enter a statement with the following details:<br>• Initials of the authors who received each award<br>• Grant numbers awarded to each author<br>• The full name of each funder<br>• URL of each funder website<br>• Did the sponsors or funders play any role in the study design, data collection and analysis, decision to publish, or preparation of the manuscript?<br>• **NO** - Include this sentence at the end of your statement: *The funders had no role in study design, data collection and analysis, decision to publish, or preparation of the manuscript.*<br>• **YES** - Specify the role(s) played.<br><br>* typeset	The funders had no role in study design, data collection and analysis, decision to publish, or preparation of the manuscript
**Competing Interests**	The authors have declared that no competing interests exist.

Use the instructions below to enter a competing interest statement for this submission. On behalf of all authors, disclose any competing interests that could be perceived to bias this work—acknowledging all financial support and any other relevant financial or non-financial competing interests.<br><br>This statement **will appear in the published article** if the submission is accepted. Please make sure it is accurate. View published research articles from *PLOS ONE* for specific examples.<br><br>**NO authors have competing interests**<br><br>Enter: *The authors have declared that no competing interests exist.*<br><br>**Authors with competing interests**<br><br>Enter competing interest details beginning with this statement:<br><br>*I have read the journal's policy and the authors of this manuscript have the following competing interests: [insert competing interests here]*<br><br>* typeset	
**Ethics Statement**<br><br>Enter an ethics statement for this submission. This statement is required if the study involved:<br><br>• Human participants<br>• Human specimens or tissue<br>• Vertebrate animals or cephalopods<br>• Vertebrate embryos or tissues<br>• Field research<br><br>Write "N/A" if the submission does not require an ethics statement.<br><br>General guidance is provided below. Consult the submission guidelines for detailed instructions. **Make sure that all**	Following to the protocol for which we obtained an ethical clearance was delivered to us by the Cameroonian national ethical committee, human volunteer was recruited among technicians working at the insectary of CRID and who by consequence are frequently exposed to mosquitoes. An informed consent was obtained after explanation of the purpose of the study.

Powered by Editorial Manager® and ProduXion Manager® from Aries Systems Corporation

**information entered here is included in the Methods section of the manuscript.**<br><br>**Format for specific study types**<br><br>**Human Subject Research (involving human participants and/or tissue)**<br>• Give the name of the institutional review board or ethics committee that approved the study<br>• Include the approval number and/or a statement indicating approval of this research<br>• Indicate the form of consent obtained (written/oral) or the reason that consent was not obtained (e.g. the data were analyzed anonymously)<br><br>**Animal Research (involving vertebrate animals, embryos or tissues)**<br>• Provide the name of the Institutional Animal Care and Use Committee (IACUC) or other relevant ethics board that reviewed the study protocol, and indicate whether they approved this research or granted a formal waiver of ethical approval<br>• Include an approval number if one was obtained<br>• If the study involved *non-human primates*, add *additional details* about animal welfare and steps taken to ameliorate suffering<br>• If anesthesia, euthanasia, or any kind of animal sacrifice is part of the study, include briefly which substances and/or methods were applied<br><br>**Field Research**<br><br>Include the following details if this study involves the collection of plant, animal, or other materials from a natural setting:<br>• Field permit number<br>• Name of the institution or relevant body that granted permission	
**Data Availability**<br><br>Authors are required to make all data underlying the findings described fully available, without restriction, and from the time of publication. PLOS allows rare exceptions to address legal and ethical	Yes - all data are fully available without restriction

concerns. See the PLOS Data Policy and FAQ for detailed information.<br><br>A Data Availability Statement describing where the data can be found is required at submission. Your answers to this question constitute the Data Availability Statement and **will be published in the article**, if accepted.<br><br>**Important:** Stating 'data available on request from the author' is not sufficient. If your data are only available upon request, select 'No' for the first question and explain your exceptional situation in the text box.<br><br>Do the authors confirm that all data underlying the findings described in their manuscript are fully available without restriction?	
**Describe where the data may be found in full sentences. If you are copying our sample text, replace any instances of XXX with the appropriate details.**<br><br>• If the data are **held or will be held in a public repository**, include URLs, accession numbers or DOIs. If this information will only be available after acceptance, indicate this by ticking the box below. For example: *All XXX files are available from the XXX database (accession number(s) XXX, XXX.*).<br>• If the data are all contained **within the manuscript and/or Supporting Information files**, enter the following: *All relevant data are within the manuscript and its Supporting Information files.*<br>• If neither of these applies but you are able to provide **details of access elsewhere**, with or without limitations, please do so. For example:<br><br>*Data cannot be shared publicly because of [XXX]. Data are available from the XXX Institutional Data Access / Ethics Committee (contact via XXX) for researchers who meet the criteria for access to confidential data.*	All relevant data are within the manuscript.

The data underlying the results presented in the study are available from (include the name of the third party and contact information or URL).<br>• This text is appropriate if the data are owned by a third party and authors do not have permission to share the data.<br><br>* typeset	
Additional data availability information:	

Powered by Editorial Manager® and ProduXion Manager® from Aries Systems Corporation

Manuscript

Click here to access/download;Manuscript;Clean version Nouage et al_Manuscript.docx

Influence of GST- and P450-based metabolic resistance to pyrethroids on blood feeding in the major African malaria vector *Anopheles funestus*

Lynda NOUAGE[1,2¥*], Emmanuel ELANGA-NDILLE[1¥*], Achille BINYANG[1,2], Magellan TCHOUAKUI[1,2], Tatiane ATSATSE[1,2], Cyrille NDO[3, 4,] , Sévilor KEKEUNOU[2], Charles S. WONDJI[1,5]

[1]Departement of Medical Entomology, Centre for Research in Infectious Diseases (CRID) P.O. BOX 13591, Yaoundé, Cameroon

[2]Department of Animal Biology and Physiology, Faculty of Science, University of Yaoundé 1, P.O. Box 812, Yaoundé, Cameroon

[3]Departement of Parasitology and Microbiology, Centre for Research in Infectious Diseases (CRID) P.O. BOX 13591, Yaoundé, Cameroon

[4]Department of Biological Sciences, Faculty of Medicine and Pharmaceutical Sciences, University of Douala, P.O. Box 24157, Douala, Cameroon.

[5]Department of Vector Biology, Liverpool School of Tropical Medicine, Pembroke Place, Liverpool L3 5QA, UK

¥: These authors contributed equally to this work

*: Corresponding authors: Emmanuel ELANGA-NDILLE (emmsdille@yahoo.fr; emmanuel.elanga@crid-cam.net) and Lynda NOUAGE (lnouage@gmail.com)

Abstract

Insecticide resistance genes are often associated with pleiotropic effects on various mosquito life-history traits. However, very little information is available on the impact of insecticide resistance on blood feeding process in mosquitoes. Here, using two recently detected DNA-based metabolic markers in the major malaria vector, *An. funestus,* we investigated how metabolic resistance genes could affect the blood meal intake.After allowing both the field F1 and lab F8 *Anopheles funestus* strains to feed on the human arm for 30 minutes, we assessed the association between key parameters of blood meal process including, probing time, feeding duration, blood feeding success, blood meal size, and markers of glutathione S-transferase (*L119F-GSTe2*) and cytochrome P450 (*CYP6P9a_R*) - mediated metabolic resistance. None of the parameters of blood meal process was associated with *L119F-GSTe2* genotypes. By contrast, for *CYP6P9a*_R, homozygous resistant mosquitoes were significantly more able to blood-feed than homozygous susceptible (OR = 3.3; CI 95%: 1.4-7.7; P =0.01) mosquitoes. Moreover, the volume of blood meal ingested by CYP6P9a-SS mosquitoes was lower than that of CYP6P9a-RS (P<0.004) and of CYP6P9a-RR (P<0.006). This suggests that *CYP6P9a* gene[illegible] linked with the feeding success and blood meal size of *An. funestus.* However, no correlation was found in the expression of *CYP6P9a* and that of genes encoding for salivary proteins involved in blood meal process.

This study suggests that P450-based metabolic resistance may influence the blood feeding process of *Anopheles funestus* mosquito and consequencetly its ability to transmit malaria parasites.

Keywords: Malaria, Mosquito, *Anopheles funestus*, Insecticide resistance, Metabolic resistance, GST, P450, Blood meal intake

Introduction

Malaria remains a major public health scourge in sub-Sahara Africa despite significant progress made since the 2000s in reducing its burden [1]. This disease is caused by a *Plasmodium* parasite transmitted by *Anopheles* mosquito species while taking a blood meal from humans. Blood feeding is essential for female mosquito's fecundity [2] as *Anopheles* species like all anautogenous female mosquitoes, require a blood meal to obtain amino acids needed to synthesize yolk proteins for eggs maturation [3,4]. The blood feeding success of mosquitoes is facilitated by the biochemical proprieties of salivary gland proteins [5]. Indeed, some salivary proteins such as anopheline antiplatelet protein (AAPP), apyrase, gambiae Salivary Gland protein 6 (gSG6) and members of D7 family have been identified as vasodilators, anti-coagulants and inhibitors of platelet aggregation allowing mosquitoes to overcome host haemostatic mechanisms and to have a successful blood meal [5-8]. Mosquito fecundity was shown to vary by source and size of the blood meal with a difference of these two parameters resulting in significant variations of the number of eggs laid by each female mosquito [4,8]. It has been shown that the number of eggs laid per female is positively associated to the amount of blood ingested as larger blood meals resulted in an increase of the number of females that developed eggs and the number of eggs per female [9,10]. The volume of blood taken by a mosquito could be affected by a range of intrinsic (host immunity) and extrinsic factors including ambient temperatures, mosquito age, parity status, gonotrophic cycle, blood feeding history and infection status [10]. More recently, it was reported that exposure to pyrethroids could also significantly influence the blood meal the process of taking a blood meal and the blood meal volume ingested by Kdr-resistant *Anopheles gambiae* females [11]. Pyrethroids (PY) are the insecticide class mostly used in the last two decades through ITNs and IRS strategies to control malaria transmission [12]. Unfortunately, the widespread use of these insecticides has favoured the development of resistance in malaria vector species

[13,14]. Resistance to pyrethroids involves two main mechanisms: (i) metabolic resistance, due to the increase expression level of detoxifying enzymes, belonging to three families: the cytochrome P450 monooxygenases, the glutathione S-transferases and the carboxylesterases; and (ii) target-site resistance due to mutations in the voltage sodium channels known as knock-down (kdr) mutations [15,16]. Although resistance mechanisms help mosquitoes to survive under continuous insecticide pressure, these actions are costly and may negatively affect mosquito's fitness including body size, adult longevity, larval development time, fecundity, fertility, mating competitiveness and blood feeding capability [17-19]. For target-site resistance, a decreased longevity and an increased larval development time have been reported in *kdr*-pyrethroid-resistant mosquitoes [20,21]. Moreover, a recent study suggested that kdr-based resistance could impact blood feeding with heterozygote (*kdr*-RS) and susceptible (*kdr*-SS) mosquitoes taking higher blood volume than homozygote (*kdr*-RR) resistant individuals [11]. In some cases, resistant mosquitoes displayed a significant advantage compared to their susceptible counterparts as shown recently for female longevity [22] and vectorial capacity [23]. However, little is known on the impact of metabolic resistance as DNA-based markers were not previously available for this mechanism; thereby limiting the ability to investigate its physiological impact on the blood feeding process in mosquitoes. However, taking advantage of the identification of the first DNA-based metabolic marker in *An. funestus* mosquito, one study reported that a GST-based metabolic resistance caused by a leucine to phenylalanine amino acid change at codon 119 in the glutathione S-transferase epsilon 2 *(L119F-GSTe2)* [24], has a detrimental impact on *An. funestus* fitness. The authors reported that field-resistant mosquitoes exhibited a reduced fecundity and slower larval development but an increased adult longevity [22]. On the other hand, a new DNA-based assay was recently designed for cytochrome P450-mediated resistance (the *CYP6P9a*-R) in *An. funestus.* This marker showed that mosquitoes carrying this P450-resistant allele survived and succeeded in blood feeding

more often than did susceptible mosquitoes when exposed to insecticide-treated nets [25]. The design of assays for both GST- and P450-based resistance now offers a great opportunity to explore how the blood feeding process is influenced by metabolic resistance mechanism in malaria vectors and further assess how resistance may impact the vectorial capacity of mosquitoes to transmit malaria in the natural environment.

Here, we investigated the effect of metabolic resistance to pyrethroids on the blood feeding process in *An. funestus*, using the two DNA-based metabolic resistance markers: *L119F-GSTe2* and *CYP6P9a*-R [24,25]. Specifically, we assessed the association between the genotypes of these metabolic resistance markers and key parameters of blood feeding including mosquito probing time, feeding duration and the blood meal size.

Material and Methods

Mosquito collection and rearing

Experiments were carried out using both field and lab strains of *An. funestus*. Field mosquitoes (F_1) were generated from indoor resting female (F0) collected in Mibellon (6°46'N, 11^{0} 70'E), a village located in a rural area of the savanna-forest region in Cameroon, Central Africa where the *L119F-GSTe2* has been reported [26]. Blood-fed field collected females were kept in paper cups and transported to the insectary of the Centre for Research in Infectious Diseases (CRID) in Yaoundé where they were kept for 4–5 days until they became fully gravid and were then induced to lay eggs using the forced eggs-laying method [27]. The eggs were placed in paper cups containing water to hatch, after which the larvae were transferred in to trays and reared to adults. To assess the effect of *CYP6P9a* marker, F_8 progenies were generated from crosses established between the pyrethroid susceptible laboratory strain (FANG) and the resistant (FUMOZ-R) lab strain. These two *An. funestus* lab strains were colonized from

mosquitoes collected in Southern Africa region. FUMOZ is a pyrethroid resistant strain established in the insectary from wild-caught *An. funestus* mosquito species from southern Mozambique [28]. The previous study reported that the over-expression of two duplicated P450 genes, *CYP6P9a* and *CYP6P9b*, constitute the main mechanism driving pyrethroid resistance in this strain [29,30] for which the *119F-GSTe2* allele is absent [24]. The FANG strain is completely susceptible to pyrethroids colonized from Calueque in southern Angola [28].

Blood feeding experiments and blood meal size quantification

Blood feeding process

Since blood meal volume has previously been reported to correlate with mosquito size [2], individuals used for blood feeding experiments were firstly starved for 24h then grouped according to their size. Mosquito size was determined by weighing (using an analytical micro-scale, SARTORIUS, Goettingen, Germany). Each starved individual (adult females aged 3-7 days) was immobilized by chilling for 2 minutes at 5°C. Each mosquito was then placed in paper cups covered with black sheet for about an hour before given a blood meal. In order to evaluate the association between metabolic resistant markers on blood meal size, mosquitoes were allowed to bite for 30 min on the bare forearm of a single human volunteer and then genotyped for L119F-GSTe2 and CYP6P9a. Following to the protocol for which we obtained an ethical clearance was delivered to us by the Cameroonian national ethical committee, human volunteer was recruited among technicians working at the insectary of CRID and who by consequence are frequently exposed to mosquitoes. An informed consent was obtained after explanation of the purpose of the study.

The duration of probing and blood feeding was assessed using a batch of 120 F_1 female field-collected mosquitoes. For this purpose, mosquitoes were individually transferred in polystyrene plastic cups covered with netting. They were allowed to rest for 15 min before

observations began. During the blood intake, each mosquito was filmed with a Digital HD Video Camera (Canon PC2154, Canon INC, Japan) placed beside the plastic cup. At the end of the time allowed for feeding, the film for each mosquito was analysed and the parameters such as probing time (defined as the time taken from initial insertion of the mouthparts in the skin until the initial engorgement of blood) [5] and total feeding duration, were recorded, using a digital timer. Due to the low density of female mosquitoes obtained at F_8 generation from crosses of the lab strain mosquitoes, experiments to estimate the probing and the feeding duration of this strain were not investigated.

To determine the blood meal size for both strains, batches of 25 mosquitoes grouped according to their weight were allowed to bite on a human arm. In this case, neither the probing time nor the feeding duration was recorded. After the trial, the whole abdomen of successfully fed mosquitoes (evident by red-coloration engorgement of the abdomen) was extracted and stored in an individual 1.5 ml microtube at – 20°C to measure the blood meal size. The rest of the carcasses as well as unfed mosquitoes were kept individually in a microtube containing RNA-later and stored at -20°C.

Blood meal size quantification

To compare the volume of blood ingested between resistant and susceptible mosquitoes, the volume of blood ingested by each mosquito was determined by quantifying the haemoglobin amount, as previously described [31]. Briefly, abdomens of blood fed mosquitoes were homogenized in 0.5 ml of Drabkin's reagent (containing 1.0g of sodium bicarbonate, 0.1g potassium carbonate, 0.05g potassium cyanide, 0.2g potassium ferricyanide all diluted in 1L of distilled water). This reagents converts the haemoglobin into haemoglobin cyanide (HiCN).

After 20 minutes at room temperature and the addition of 0.5 ml of chloroform solution, samples were centrifuged at 5600 rpm (3512 rcf) for 5 min. The aqueous supernatant containing HiCN was transferred in a new 1.5 ml microtube. An aliquot of 200µl from each sample was transferred to a microplate and the optical density (OD) read at a wavelength of 620nm in a spectrophotometer (EZ Read 400, biochrom, Cambridge, UK). OD for each sample were read in duplicate and the average value between the two replicates was considered as OD value of the sample. In parallel, OD read on various amounts of human volunteer blood added to Drabkin's reagent in individual microtubes were used as control to generate calibration curves and the regression line used to assess the relationship between OD and blood volume. For each sample, the blood meal size was estimated according to the weight by dividing the blood volume estimated using the regression line by the average weigh of each batch of mosquitoes constituted after the weighing. The blood meal size was then expressed in µL of blood per mg of weight.

Molecular species identification

To determine the species composition of *An. funestus* group among the samples, genomic DNA (gDNA) was extracted from both blood-fed and unfed mosquitoes using the Livak protocol [32]. Instead of using the whole body as done for unfed mosquitoes, DNA was extracted from the carcasses of fed mosquitoes after removing the abdomen for blood volume quantification. The concentration and purity of the extracted gDNA were subsequently determined using a NanoDrop™ spectrophotometer (Thermo Scientific, Wilmington, USA) before storage at −20 °C. An aliquot of gDNA extracted from field-collected strain was used for molecular identification by a polymerase chain reaction [33].

Genotyping of *L119F-GSTe2* mutation in field-collected strain

The *L119F-GSTe2* mutation was genotyped using gDNA extracted from carcasses of field-collected strains following an allele specific PCR diagnostic assay previously described [22]. The primers sequences are given in table S1. PCR was performed in Gene Touch thermal cycler (Model TC-E-48DA, Hangzhou, 310053, China), in a reaction volume of 15 µl using 10 µM of each primer, 10X Kapa Taq buffer A, 0.2 mM dNTPs, 1.5 mM $MgCl_2$, 1U Kapa Taq (Kapa Biosystems, Wilmington, MA, USA) and 1µl of genomic DNA as template. The cycle parameters were: 1 cycle at 95 °C for 2 min; 30 cycles of 94 °C for 30 s, 58 °C for 30 s, 72 °C for 1 min and then a final extension at 72 °C for 10 min. The PCR products were size separated on a 2 % agarose gel stained with Midori Green Advance DNA Stain (Nippon genetics Europe GmbH) and visualised using a gel imaging system. The size of the diagnostic band was 523 bp for homozygous resistant (RR) and 312 bp for homozygous susceptible (SS), while heterozygous (RS) showed the two bands.

Genotyping of *CYP6P9a*-R allele in lab strain mosquitoes

The *CYP6P9a* resistance marker was genotyped using the protocol recently designed by [25]. A PCR-RFLP were carried out using gDNA extracted from the carcasses of F_8 generation individuals obtained from the reciprocal crosses between FANG and FUMOZ strains used for blood feeding. Briefly, a partial *CYP6P9a* upstream region was amplified in a final volume of 15µl PCR mixture containing 10X Kapa Taq buffer A (Kapa Biosystems, Wilmington, MA, USA), 5 U/µl KAPA taq, 25µM dNTP, 25µM $MgCl_2$, 10 mM of each primer, 10.49µl of dH_2O and 1µl of genomic DNA. The PCR cycle parameters were as follows: the initial denaturation step at 95°C for 5 minutes followed by 35 cycles of 94°C for 30 seconds, 58°C for 30 seconds

and 72°C for 45 seconds and a final extension step of 72°C for 10 minutes. The PCR products were size separated on a 1.5 % agarose gel stained with Midori Green Advance DNA Stain (Nippon genetics Europe GmbH) and visualised using a gel imaging system to confirm the product size (450bp). Then, the PCR product was incubated at 65°C for 2 hours. This was done in 0.2ml PCR strip tubes using 5µl of PCR product, 1µl of cutSmart buffer, 0.2µl of 2 units of Taq1 enzyme (New England Biolabs, catalog: ER0672) and 3.8µl of dH20. Size separation was done on a 2.0% agarose gel stained with Midori Green Advance DNA Stain at 100V for 30 minutes. The gel was visualised using the gel imaging system.

Gene expression profiling of major salivary genes encoding proteins involved in blood meal process.

The expression profiles of a set of salivary genes encoding for proteins involved in blood meal process was compared between CYP6P9a-RR, CYP6P9a-RS and CYP6P9a-SS *An. funestus* mosquitoes. For each gene, two pairs of exon-spanning primers was designed for each gene using Primer3 online software (v4.0.0; http://bioinfo.ut.ee/primer3/) and only primers with PCR efficiency between 90 and 110% determined using a cDNA dilution series obtained from a single sample, were used for qPCR analysis. Taking into account this criteria of efficiency, only the AAPP and four members of D7 family genes (D7r1, D7r2, D7r3, and D7r4) were used for this analysis. Primers are listed in Table S1. Total RNA was extracted from three batches of 10 whole females of 3–5 days old from CYP6P9a-RR, CYP6P9a-RS and CYP6P9a-SS mosquitoes. RNA was isolated using the RNAeasy Mini kit (Qiagen) according to the manufacturer's instructions. The RNA quantity was assessed using a NanoDrop ND1000 spectrophotometer (Thermo Fisher) and 1µg from each of the three biological replicates for each batch of mosquitoes was used as a template for cDNA synthesis using the SuperScript III

(Invitrogen, Waltham, Massachusetts, USA) with oligo-dT20 and RNase H, following the manufacturer's instructions. The qPCR assays were carried out in a MX 3005 real-time PCR system (Agilent, Santa Clara, CA 95051, United States) using Brilliant III Ultra-Fast SYBR Green qPCR Master Mix (Agilent). A total of 10 ng of cDNA from each sample was used as template in a three-step program involving a denaturation at 95 °C for 3 min followed by 40 cycles of 10 s at 95 °C and 10 s at 60 °C and a last step of 1 min at 95 °C, 30 s at 55 °C, and 30 s at 95 °C. The relative expression and fold-change of each target gene in CYP6P9a-RR and CYP6P9a-RS relative to CYP6P9a-SS was calculated according to the $2^{-\Delta\Delta CT}$ method incorporating PCR efficiency after normalization with the housekeeping RSP7 ribosomal protein S7 (VectorBase ID: AFUN007153;) and the actin 5C (vectorBase ID: AFUN006819) genes for *An. funestus*.

Statistical analysis

All analyses were conducted using GraphPad Prism version 7.00 software. We estimated a Fisher's exact probability test and the odds-ratio of *L119F-GSTe2* and *CYP6P9a* genotypes (homozygous resistant = RR, heterozygote resistant=RS and homozygous resistant =SS) and both susceptible (S) and resistant (R) alleles. This allowed to the assessment of association between: a) insecticide resistance and mosquito's weight by comparing the proportions of the genotypes of both genes in each group established after weighing; b) blood feeding success and insecticide resistance by comparing the proportion of each genotype in both fed and unfed mosquitoes. The feeding duration was grouped into four intervals with 1 minute (60s) amplitude for each interval.The duration of probing and feeding was analysed by comparing the proportion of mosquitoes (for *L119F-GSTe2* and *CYP6P9a*) with different genotypes in each defined intervals using chi-square test. After estimating the median of weighted blood meal for each genotype, Kruskal-Wallis and Mann-Whitney tests were used to

compare the differences between more than two groups and between two groups, respectively as Shapiro welch test showed non normal distribution.

Results

Metabolic resistance genes and *An. funestus* mosquito's weight

A total of 1,200 and 273 female mosquitoes were weighed, respectively for field strain (F_1 generation) and lab strain (F_8 generation). The mean weight of a mosquito was 0.9 ± 0.010 mg (minimum = 0.2 mg; maximum = 2.3mg) and 0.89 ± 0.016 mg (minimum = 0.2 mg; maximum = 1.7mg) for field and lab strain respectively. No significant difference was found between the mean weights of two strains. For all the analyses, we arbitrarily grouped mosquitoes according to their weight values, into two different classes as followed: [0 - 1.0] mg and [1.1 - 2.3] mg. Analysis of the distribution of *L119F-GSTe2* mutation genotypes in each class of field strain mosquitoes showed no association between the mosquito's weight and *L119F-GSTe2* genotypes (χ^2 = 0.15; *p* = 0.9; OR =1.2, 95%CI: 0.3742 - 4.176, for RR *vs* RS; OR=1.1, 95%CI: 0.3659 - 3.606 for RR *vs* SS; OR = 0.9, 95%CI: 0.4943 - 1.709 for RS *vs* SS) (Figure 1 and Table 1). This absence of correlation between the *L119F-GSTe2* genotypes and the weight of mosquitoes was confirmed at the allele level (OR=1; 95%: CI: 0.5–2.0; *p* =0.5) showing that the L119F mutation may not associated with the weight of this *An. funestus* field population (Table 1). In contrast, a significant association was observed between *CYP6P9a* genotypes and the weight of mosquito (χ^2= 29.54, p<0. 0001). Indeed, proportions of RR and RS genotypes were higher than that of SS in the lowest weight class, whereas, for larger weight, mosquitoes with SS genotype were more abundant (67.2%). This association is further supported by odds ratio estimates showing that proportions of homozygote resistant (RR) (OR=5.4; CI 95%: 2.3-12.7; *p*<0.0001) and heterozygote (RS) (OR=5.6; CI 95%: 2.8-11.1;

p<0.0001) mosquitoes are significantly higher in lowest weight class than the larger one when compared to homozygote susceptible mosquitoes (Table 1). Overall, mosquitoes harbouring the *CYP6P9a*-S susceptible allele displayed higher weight compared to those with the *CYP6P9a*-R resistant allele (OR=2.8; CI 95%: 1.5–5.0; p =0.0003 (Table 1) suggesting that over-expression of the *CYP6P9a* gene is reducing the weight of pyrethroid resistant *An. funestus* mosquitoes.

Table 1: level of association of *L119F-GSTe2* and *CYP6P9a*-R genotypes with mosquito weight by comparing low (0-1.0mg) and high (1-2.4mg) weight samples.

	L119F-GSTe2		***CYP6P9a-R***	
**Genotypes**	**Odds ratio**	***p*-value**	**Odds ratio**	***p*-value**
**RR vs SS**	1.1 (0.4-3.6)	0.5	5.4 (2.3-12.7)	< 0.0001
**RS vs SS**	0.9 (0.5-1.7)	0.4	5.6 (2.8-11.1)	< 0.0001
**RR vs RS**	1.2 (0.4-4.1)	0.5	1.0 (0.5-2.3)	0.5
**S vs R**	1 (0.5-2.0)	0.5	2.8 (1.5-5.0)	0.0003

SS: homozygote susceptible; RR: homozygote resistant; RS: heterozygote; * significant difference p < 0.05.

Influence of *L119F-GSTe2* and *CYP6P9a* mutations on *An. funestus* blood feeding success

***L119F-GSTe2*:** out of the 1,200 individuals from field strain mosquitoes that were allowed to take a blood meal, 457 (39.6%) successfully fed whereas 743 did not. Among blood-fed mosquitoes, a total of 360 were successfully genotyped and 7% (24/360) were homozygous resistant (RR), 28% (103/360) were heterozygous resistant (RS) and 65% (233/360) were homozygous susceptible (SS) (Figure 2a). On the other hand, out of the 300 unfed mosquitoes randomly selected for genotyping, 5% (15/300), 32% (62/300) and 63% (189/300), were

homozygous resistant, heterozygotes and homozygote susceptible, respectively (Figure 2a). However, the distribution of L119F genotypes was not statistically different between blood-fed and unfed mosquitoes (χ^2=0.63, *p*=0.7). Furthermore, estimation of odds ratio. (OR=1; CI 95%: 0.5–2.0; *p* = 0.6) showed overall that mosquitoes bearing the 119F-R resistant allele have the same chance to have a successful blood feeding than those with the 119F-S susceptible alleles (Table 2). This suggests that the ability to take blood is not associated with the *L119F-GSTe2* mutation in *An. funestus*.

Table 2: Assessment of the association of *L119F-GSTe2* and *CYP6P9a-R* mutations with *An. funestus* mosquito blood feeding

	L119F-GSTe2		***CYP6P9a-R***	
**Genotypes**	**Odds ratio**	***p*-value**	**Odds ratio**	***p*-value**
**RR vs SS**	0.7 (0.2-2.4)	0.4	3.3 (1.4-7.7)	0.01
RS vs SS	1.1 (0.62-2.1)	0.4	1.7 (0.9-3.1)	0.1
RR vs RS	0.6 (0.2-2.3)	0.3	1.9 (0.9-4.4)	0.1
R vs S	1 (0.5-2.0)	0.6	1.8 (1.1-3.2)	0.04

SS: homozygote susceptible; RR: homozygote resistant; RS: heterozygote; * significant difference p < 0.05.

CYP6P9a-R: Among a total of 273 mosquitoes that were offered a blood meal 140 successfully fed (51.3%) whereas, 133 did not. Out of the 140 mosquitoes that blood-fed, 134 were successfully genotyped for *CYP6P9a*-R allele revealing that 23% (31/134), 50% (67/134) and 27% (36/134) were homozygote resistant CYP6P9a-RR, heterozygotes CYP6P9a-RS and homozygote susceptible CYP6P9a-SS, respectively (Figure 2b). Among the unfed mosquitoes, 11.3% (15/133) were homozygote resistant CYP6P9a-RR, 47.4% (63/133) heterozygotes and 41.3% (55/133) were homozygote susceptible CYP6P9a-SS. The estimation of odds ratio

showed that homozygote resistant CYP6P9a-RR mosquitoes are significantly more able to blood feed than homozygote susceptible (OR = 3.33; CI 95%: 1.4 -7.7; *p* =0.01). No difference was observed between heterozygote and homozygote resistant CYP6P9a-RR (OR= 1.9, 95%CI: 0.9-4.4; p=0.1) neither with homozygote susceptible CYP6P9a-SS (OR= 1.7, 95%CI: 0.9-3.1; p=0.1) mosquitoes (Table 2). Moreover, it was overall observed that mosquitoes with the *CYP6P9a*-R resistant allele have a greater chance to blood feed than those bearing the susceptible allele (OR = 1.9; CI 95%: 1.03-3.2; *p* =0.04) (Table 2).

Association between the *L119F-GSTe2* mutation and probing/ blood feeding duration

Out of the 120 mosquitoes that were individually filmed to assess the influence of insecticide resistance genes on the probing and feeding duration, 7 (6.14%), 40 (35.08%) and 67 (58.77%) were genotyped as homozygous resistant 119F/F-RR, heterozygous L119F-RS and homozygous susceptible L/L119, respectively. Overall, regardless of the genotype, the median value of mosquito's probing duration was 49.5 seconds (minimum = 4s and maximum = 290s). No difference was observed in the probing time of resistant mosquitoes 119F/F-RR (Median = 53 seconds) and heterozygotes L119F-RS (Median = 52s) compared to the homozygote susceptible L/L119 (Median = 52s).

Regarding the blood feeding duration, it was observed that the median and mean time for a mosquito to have a full blood meal was 249.5 seconds and 303 ± 181 seconds respectively, with a minimum = 68 seconds and a maximum =772 seconds. The feeding duration was longer (median=269s) in L/L119 mosquitoes compared to L119F-RS (229.5s) and 119F/F-RR (214s) but the difference was not statistically significant (*p*=0.19, Kruskal-Wallis test).

Effect of *L119F-GSTe2* and *CYP6P9a*-R mutations on the blood meal size of *An. funestus*

L119F-GSTe2 : From 457 individuals that took a full blood meal it was observed that the average weighted blood meal of a mosquito regardless of the *L119F-GSTe2* genotype was 3.4± 1.3 µl/mg (minimum = 1.2 µl/mg; maximum = 9.2 µl/mg). However, the weighted blood meal was not significantly different (P=0.17; Kruskal-Wallis test; Figure 3a) in homozygote susceptible L119-SS (3.0µl/mg) compared to homozygote resistant L119-RR (2.8µl/mg) and heterozygote L119F-RS (3.3µl/mg) mosquitoes. This result suggests that the *L119F-GSTe2* mutation is not associated with the volume of blood meal ingested by *An. funestus*.

CYP6P9a-R: The influence of the *CYP6P9a*-R mutation on the volume of blood meal taken by *An. funestus*, was assessed using the 134 blood fed mosquitoes that were successfully genotyped for *CYP6P9a*-R allele. Overall, irrespective of the genotype, the mean weighted blood volume ingested by a mosquito was 4.8 ± 2 µl/mg (minimum = 2 µl/mg; maximum = 13.3µl/mg). However, the weighted blood meal volume of CYP6P9a-SS mosquitoes (Median = 3.71µl/mg) was lower than that of CYP6P9a-RS (Median = 4.73 µl/mg) and of CYP6P9a-RR (Median = 4.78 µl/mg) (Figure 3; p<0.004 for RS vs SS and p<0.006 for RR vs SS, Mann-Whitney test). No difference in the volume of the blood meal was observed between CYP6P9a-RR and CYP6P9a-RS mosquitoes (P=0.7; Mann-Whitney test). This result suggests that the over-expression of *CYP6P9a* gene is associated with an increase of the volume of the blood meal ingested by *An. funestus*.

Expression profile of *AAPP* and *D7* family salivary genes according to *CYP6P9a*-R genotypes

Due to the association observed between the *CYP6P9a*-R genotypes and blood feeding, an attempt was made to assess whether the genotypes of this gene could be possible associated with the expression profile of key salivary genes. The expression level of AAPP and 4 members of the D7 family salivary genes (D7r1, D7r2, D7r3 and D7r4) was analysed and compared between homozygous resistant (CYP6P9a-RR), heterozygous (CYP6P9a-RS) and homozygous susceptible genotype (CYP6P9a-SS) mosquitoes. No significant difference in the expression level of these genes was observed between the three types of mosquitoes although D7 familly genes appeared slightly over-expressed in CYP6P9a-RR and CYP6P9a-RS when compared to CYP6P9a-SS (Figure 4). This result suggests that *CYP6P9a*-R genotypes do not influence the expression profile of both AAPP and D7 family genes in the salivary glands of *An. funestus* mosquitoes.

Discussion.

Recently, mutations in the *GST* epsilon 2 and in the promoter region of the cytochrome P450 *CYP6P9a*, were described as robust molecular markers for tracking metabolic resistance in pyrethroids resistant populations of *An. funestus* [24,25]. Using these two key markers, this study assess the possible association of GST- and P450-based metabolic resistance to pyrethroids on the feeding process and blood meal volume of *An. funestus*.

Association of metabolic resistance on blood feeding success

The present study revealed that *CYP6P9a* but not the *L119F-GSTe2* mutation could impact the blood feeding success of *An. funestus* mosquito as possessing the *CYP6P9a* resistant allele increased the likelihood of being successful in blood-feeding. Such selective advantage of *CYP6P9a* resistance allele was also previously reported in a semi-field study in experimental hut trial which observed that homozygous CYP6P9a-RR mosquitoes were significantly more likely to blood feed than susceptible SS [25]. This result suggests that CYP6P9a -mediated metabolic resistance might influence the ability of *An. funestus* mosquito to blood feed. In contrast, the absence of association observed here for the *L119F-GSTe2* mutation needed to be confirmed by further studies as the low sample of L119F-RR homozygous resistance mosquitoes might have biased our analysis. This low number of L119F-RR mosquitoes could itself be linked to unsuccessfully genotyping of this marker in approximately 20% of samples analysed in this study. This important point highlights the need for further studies to improve the optimization of the protocol used in this study for the genotyping of the L119F-GSTe2 mutation. On the other hands, the mechanism whereby *CYP6P9a*-R resistant allele could influence mosquito feeding is unknown and was not investigated in the present study. One hypothesis to explain this association could be related to the motivation of mosquito to blood feed. In fact, it has been reported that some mosquito individuals that emerged with insufficient teneral reserves require an initial blood meal to compensate for insufficient teneral reserves rather than for egg development during their first gonotrophic cycle [34-36]. This phenomenon is mostly observed in smaller female mosquitoes that emerge with insufficient reserve [2]. Thus, we can presume that *CYP6P9a* resistant mosquitoes which were found significantly smaller than susceptible in the present study were more motivated to blood feed as they were probably

the ones requiring more to compensate for their insufficient teneral reserves. However, it's important to note that it was surprising and unusual to observed CYP6Pa-SS mosquitoes bigger that CYP6Pa-RR ones since previous studies often reported larger mosquitoes tend to be more tolerant of insecticides, or that resistant phenotypes are associated with larger body sizes[37,38]. This unusual observation could be explained by the fact that, instead of using dead dried and unfed mosquito as usually done, in the present study the weight was estimated using alive fresh mosquitoes which were fed with sugar solution until 24 hours before being weighted. With this approach, mosquito's body weight may have been influenced by water and/or elements of sugar digestion that are eliminated when the mosquito is dried. This point highlight the need to perform further studies working for instance with dried mosquitoes before confirm our hypothesis about the association of teneral reserve and CYP6P9a mutation. One other approach could be to carry out calorimetric assays comparing teneral reserve between CYP6Pa-SS and CYP6Pa-RR mosquitoes. These further studies would certainly be more informative on the influence of metabolic resistance on the motivation of *An. funestus* mosquito to blood feed.

Influence of metabolic resistance on probing time and feeding duration

The influence of metabolic resistance on probing time and feeding duration was assessed in the present study only for *L119F-GSTe2* mutation. Results revealed no significant association of this metabolic resistance gene on the time spent by a mosquito to probe. The absence ofassociation of insecticide resistance on mosquito probing time was also reported for the *knock-down* (*kdr*) resistance gene in *Anopheles gambiae* with no difference in the probing time noticed between genotypes (RR, RS and SS) after exposure to untreated and insecticide-treated net [11]. This seems to indicate that insecticide resistance might not impact the probing duration

of *Anopheles* mosquito during blood feeding. However, this hypothesis must be taken with caution as, to our knowledge, and the exception of the present study as well as the one of Diop et *al*, very little information is available on the impact of insecticide resistance on the probing time during mosquito blood-feeding. In the other hand, even if the difference was not statistically significant, mosquitoes possessing an *119F-GSTe2* resistant allele (both homozygous and heterozygous) spent less time taking their blood meal than susceptible. This corroborate with observation previously made for *kdr* mutation in *An. gambiae* with lower feeding duration for homozygous resistant mosquitoes than heterozygote and homozygous susceptible [11]. The non-significant result observed may be due to the low number of resistant mosquitoes in the present study. However, from the results, it could be hypothesized that *L119F-GSTe2* mutation might confer an advantage to homozygous resistant mosquitoes as it was previously reported that rapid feeding reduces the risk to be killed by the host defensive behaviour [11,39].

Effect of metabolic resistance on blood meal volume

In this study, we observed that the volume of blood ingested by a mosquito during a single blood feeding was associated with the genotype of the P450 *CYP6P9a* but not with the *L119F-GSTe2*-based metabolic resistance. This suggests that mechanisms involved in metabolic resistance to pyrethroids in *An. funestus* might influence mosquito life-traits differently. However, as already discussed above, we cannot exclude that the absence of the influence observed for *L119F-GSTe2* gene might also be related to the low number of L119F-RR mosquitoes used in the present study. This latter hypothesis seems moreover reinforced by the results of previous studies showing *L119F-GSTe2* mutation [22] and *CYP6P9a*-R resistance gene [40] influencing *An. funestus* fecundity in the same way. The positive association between

CYP6P9a-R resistant allele and the volume of blood meal is in line with the work of Okoye and collaborators reporting that pyrethroid resistance mechanism in southern African *An. funestus* cause no reduction in fitness of this mosquito [41]. Thus, our finding suggests that the over-expression of CYP6P9a gene might probably not compromise the volume of blood ingested of individual mosquitoes carrying the CYP6P9a-R resistant allele. Given that activity of P450 monoxygenases as well as blood meal digestion, have been reported to generate an excess production of reactive oxygen species (ROS) increasing oxidative stress which could induce several damages in the mosquito's system that can result to death [42,43], it could have been expected to see CYP6P9a-RR mosquitoes taking lower blood to reduce negative effects of oxidative stress. This observation could certainly be explained by the ability of *Anopheles* mosquitoes to cope with oxidative damage after blood feeding by increasing the antioxidant activity enzymes including, Cu Zn and Mn superoxide dismutase (SOD), catalase, glutathione peroxides and thioredoxin reductase [44].. This suggests that association between the *CYP6P9a*-R resistant allele and mosquito's blood meal size could be an indirect consequence of some other physiological activities. For instance, because *CYP6P9a* resistant mosquitoes were significantly smaller than their susceptible counterparts, and noting that it has been demonstrated that the amount of teneral reserves is proportional to the body size of mosquito [2], we can presume that the high blood meal volume ingested by CYP6P9a-RR mosquitoes might be as a result of a need to compensate for the limited teneral reserves post emergence. In this case, the association observed here could be an indirect consequence of the negative association of *CYP6P9a*-R resistant allele recently observed on the larval development of *An. funestus* [40] resulting to a small body size, and by consequence to insufficient teneral reserves for resistant mosquitoes. Indeed, it was demonstrated that encountering a nutritional environment by *Anopheles* larvae strongly influences adult fitness-related traits such as body size and teneral metabolic reserves [2,31,45]. However, our finding did not corroborate with

the positive association previously reported between the volume of ingested blood meal and mosquito body size [2]. Further studies will help elucidate the underlying reason of this correlation between *CYP6P9a* genotypes and blood meal size.

Possible association *of CYP6P9a-R* resistant allele on salivary gland genes expression

To obtain a successful blood meal, a female mosquito must balance the risk of death caused by host defensive behavior against the benefits to feed on a host species that maximize fertility [46]. Salivary components permit mosquitoes to reduce their engorgement time and increase their likelihood of survival [5]. In the present study, we assessed the level of expression of genes encoding for some salivary proteins known to be involved on blood intake process of mosquitoes such as, AAPP and members of D7 family proteins [6,47,48]. The comparative analysis of the expression level of these genes between *CYP6P9a* genotypes showed no significant difference between mosquitoes bearing the resistant allele and those with the susceptible one. This result suggests that the expression of AAPP and D7 family salivary genes are not associated to the *CYP6P9a* mutation. This observation is intriguing as some salivary genes such as D7 family genes were previously reported to be over-expressed in resistant *An. funestus* mosquito compared to susceptible strain [24,49-52]. The lack of significance observed with the differential expression of genes in the present study could be explained by the fact that, our analyses in this study were performed on mosquitoes obtained after crosses between two different strains and therefore sharing the same background, while other studies compared insecticide resistant field /laboratory mosquitoes and susceptible laboratory strains with different genetic background [24,49,53]. The absence of influence of the *CYP6P9a* gene on the expression level of salivary gland genes involved in the blood feeding process observed in the present study appears to indicate that the association found between this gene and the size of

blood meal taken by *An. funestus* mosquito might not be related to the expression of these salivary genes encoding proteins which mediate the blood meal process.

This study revealed that GSTe2-mediated resistance is not associated with the blood meal intake of *An. funestus* mosquitoes, whereas *CYP6P9a*-based resistance to pyrethroids is associated with a feeding success and a higher blood meal size. However, this influence on *Anopheles funestus* blood meal intake is not associated with differential expression of major salivary gland proteins involved in blood-feeding. Given the rapid growth of insecticide resistance, it would be interesting to study how this association could affect the fecundity and the vectorial capacity of *An. funestus* mosquitoes.

Author Contributions: E.E.N and C.S.W conceived the study; EEN, L.N, C.N SK and C.S.W designed the study; E.E.N, .L.N, A.B, T.A and M.T carried out the sample collection; L.N, A.B, and T.A reared and maintained the strain in the insectary; E.E.N, L.N A.B, and T.A performed blood feeding experiments. L.N, T.A and M.T performed the molecular analyses; E.E.N, L.N, A.B, M.T and C.N analyzed the data; E.E.N, L.N and C.S.W wrote the manuscript. M.T C.N and S.K reviewed the manuscript. All authors approved the manuscript.

Ethical approval and consent to participate

Ethical clearance was obtained from the National Ethics Committee of Cameroon’s Ministry of Public Health (N°2018/04/1000/CE/CNERSH/SP) in conformity to the WMA Declaration of Helsinki. Informed verbal consent was obtained from household owners for using their houses for mosquito collection.

Acknowledgments: We thank Dr Micheal KUSIMO and Mr Francis NKEMNGO; native speakers of English language for accepting to review and edit this paper.

Conflicts of Interest: The authors declare no conflicts of interests.

References

1. WHO (2019) World Malaria Report 2019, Geneva: World Health Organization; 2019. Licence: CC BY-NC-SA 3.0 IGO.

2. Briegel H (1990) Fecundity, metabolism, and body size in *Anopheles* (Diptera: *Culicidae*), vectors of malaria. Journal of medical entomology 27: 839-850.

3. Hurd H (2003) Manipulation of medically important insect vectors by their parasites. Annual review of entomology 48: 141-161.

4. Roitberg BD, Gordon I (2005) Does the *Anopheles* blood meal-fecundity curve, curve? Journal of Vector Ecology 30: 83.

5. Ribeiro J (2000) Blood- feeding in mosquitoes: probing time and salivary gland anti- haemostatic activities in representatives of three genera (*Aedes, Anopheles, Culex*). Medical and veterinary entomology 14: 142-148.

6. Islam A, Emran TB, Yamamoto DS, Iyori M, Amelia F, et al. (2019) Anopheline antiplatelet protein from mosquito saliva regulates blood feeding behavior. Scientific reports 9: 3129.

7. Thiévent K, Hauser G, Elaian O, Koella JC (2019) The interaction between permethrin exposure and malaria infection affects the host-seeking behaviour of mosquitoes. Malaria journal 18: 79.

8. Taylor P, Hurd H (2001) The influence of host haematocrit on the blood feeding success of *Anopheles* stephensi: implications for enhanced malaria transmission. Parasitology 122: 491-496.

9. Edman JD, Lynn HC (1975) Relationship between blood meal volume and ovarian development in *Culex nigripalpus* (Diptera: *Culicidae*). Entomologia experimentalis et applicata 18: 492-496.

10. Lehane MJ (2005) The biology of blood-sucking in insects: Cambridge University Press.

11. Diop MM, Chandre F, Rossignol M, Porciani A, Chateau M, et al. (2019) Sub-lethal insecticide exposure affects host biting efficiency of Kdr-resistant *Anopheles gambiae*. bioRxiv: 653980.

12. Bhatt S, Weiss D, Cameron E, Bisanzio D, Mappin B, et al. (2015) The effect of malaria control on *Plasmodium falciparum* in Africa between 2000 and 2015. Nature 526: 207.

13. Ranson H, N'guessan R, Lines J, Moiroux N, Nkuni Z, et al. (2011) Pyrethroid resistance in African anopheline mosquitoes: what are the implications for malaria control? Trends in parasitology 27: 91-98.

14. Strode C, Donegan S, Garner P, Enayati AA, Hemingway J (2014) The impact of pyrethroid resistance on the efficacy of insecticide-treated bed nets against African anopheline mosquitoes: systematic review and meta-analysis. PLoS medicine 11.

15. Martinez- Torres D, Chandre F, Williamson M, Darriet F, Berge JB, et al. (1998) Molecular characterization of pyrethroid knockdown resistance (kdr) in the major malaria vector *Anopheles gambiae ss.* Insect molecular biology 7: 179-184.

16. Ranson H, Jensen B, Vulule J, Wang X, Hemingway J, et al. (2000) Identification of a point mutation in the voltage- gated sodium channel gene of Kenyan *Anopheles gambiae* associated with resistance to DDT and pyrethroids. Insect molecular biology 9: 491-497.

17. Berticat C, Boquien G, Raymond M, Chevillon C (2002) Insecticide resistance genes induce a mating competition cost in *Culex pipiens* mosquitoes. Genetics Research 79: 41-47.

18. Saingamsook J, Yanola J, Lumjuan N, Walton C, Somboon P (2019) Investigation of Relative Development and Reproductivity Fitness Cost in Three Insecticide-Resistant Strains of *Aedes aegypti* from Thailand. Insects 10: 265.

19. Rowland M (1991) Behaviour and fitness of γHCH/dieldrin resistant and susceptible female *Anopheles gambiae* and *An. stephensi mosquitoes* in the absence of insecticide. Medical and Veterinary Entomology 5: 193-206.

20. Kumar K, Sharma AK, Kumar S, Patel S, Sarkar M, et al. (2011) Multiple insecticide resistance/susceptibility status of *Culex quinquefasciatus,* principal vector of bancroftian filariasis from filaria endemic areas of northern India. Asian Pacific journal of tropical medicine 4: 426-429.

21. Martins AJ, Bellinato DF, Peixoto AA, Valle D, Lima JBP (2012) Effect of insecticide resistance on development, longevity and reproduction of field or laboratory selected *Aedes aegypti* populations. PloS one 7: e31889.

22. Tchouakui M, Riveron JM, Djonabaye D, Tchapga W, Irving H, et al. (2018) Fitness Costs of the Glutathione S-Transferase Epsilon 2 (*L119F-GSTe2*) Mediated Metabolic Resistance to Insecticides in the Major African Malaria Vector *Anopheles Funestus*. Genes 9: 645.

23. Tchouakui M, Fossog BT, Ngannang BV, Djonabaye D, Tchapga W, et al. (2019) Investigation of the influence of a glutathione S-transferase metabolic resistance to pyrethroids/DDT on mating competitiveness in males of the African malaria vector, *Anopheles funestus*. Wellcome open research 4.

24. Riveron JM, Yunta C, Ibrahim SS, Djouaka R, Irving H, et al. (2014) A single mutation in the *GSTe2* gene allows tracking of metabolically based insecticide resistance in a major malaria vector. Genome biology 15: R27.

25. Weedall GD, Mugenzi LM, Menze BD, Tchouakui M, Ibrahim SS, et al. (2019) A cytochrome P450 allele confers pyrethroid resistance on a major African malaria vector, reducing insecticide-treated bednet efficacy. Science translational medicine 11: eaat7386.

26. Menze BD, Riveron JM, Ibrahim SS, Irving H, Antonio-Nkondjio C, et al. (2016) Multiple insecticide resistance in the malaria vector *Anopheles funestus* from Northern Cameroon is mediated by metabolic resistance alongside potential target site insensitivity mutations. PloS one 11: e0163261.

27. Morgan JC, Irving H, Okedi LM, Steven A, Wondji CS (2010) Pyrethroid resistance in an *Anopheles funestus* population from Uganda. PLoS One 5: e11872.

28. Hunt R, Brooke B, Pillay C, Koekemoer L, Coetzee M (2005) Laboratory selection for and characteristics of pyrethroid resistance in the malaria vector *Anopheles funestus*. Medical and veterinary entomology 19: 271-275.

29. Riveron JM, Irving H, Ndula M, Barnes KG, Ibrahim SS, et al. (2013) Directionally selected cytochrome P450 alleles are driving the spread of pyrethroid resistance in the major malaria vector *Anopheles funestus.* Proceedings of the National Academy of Sciences 110: 252-257.

30. Wondji CS, Irving H, Morgan J, Lobo NF, Collins FH, et al. (2009) Two duplicated P450 genes are associated with pyrethroid resistance in *Anopheles funestus*, a major malaria vector. Genome research 19: 452-459.

31. Briegel H, Lea AO, Klowden MJ (1979) Hemoglobinometry as a method for measuring blood meal sizes of mosquitoes (Diptera: Culicidae). Journal of Medical Entomology 15: 235-238.

32. Livak KJ (1984) Organization and mapping of a sequence on the Drosophila melanogaster X and Y chromosomes that is transcribed during spermatogenesis. Genetics 107: 611-634.

33. Koekemoer L, Kamau L, Hunt R, Coetzee M (2002) A cocktail polymerase chain reaction assay to identify members of the *Anopheles funestus* (Diptera: Culicidae) group. The American journal of tropical medicine and hygiene 66: 804-811.

34. Klowden MJ, Lea AO (1978) Blood meal size as a factor affecting continued host-seeking by *Aedes aegypti* (L.). The American journal of tropical medicine and hygiene 27: 827-831.

35. Ramasamy M, Srikrishnaraj K, Hadjirin N, Perera S, Ramasamy R (2000) Physiological aspects of multiple blood feeding in the malaria vector *Anopheles tessellatus*. Journal of insect physiology 46: 1051-1059.

36. Takken W, van Loon JJ, Adam W (2001) Inhibition of host-seeking response and olfactory responsiveness in *Anopheles gambiae* following blood feeding. Journal of insect physiology 47: 303-310.

37. Jeanrenaud AC, Brooke BD, Oliver SV (2019) The effects of larval organic fertiliser exposure on the larval development, adult longevity and insecticide tolerance of zoophilic members of the *Anopheles gambiae* complex (Diptera: *Culicidae*). PloS one 14.

38. Owusu HF, Chitnis N, Müller P (2017) Insecticide susceptibility of *Anopheles* mosquitoes changes in response to variations in the larval environment. Scientific reports 7: 1-9.

39. Lyimo IN, Haydon DT, Mbina KF, Daraja AA, Mbehela EM, et al. (2012) The fitness of African malaria vectors in the presence and limitation of host behaviour. Malaria journal 11: 425.

40. Tchouakui M, Jacob Riveron M, Mugenzi LMJ, Djonabaye D, Wondji MJ, et al. (2020) Cytochrome P450 metabolic resistance (CYP6P9a) to pyrethroids imposes a fitness cost in the major African malaria vector *Anopheles funestus*. Genetics Society.

41. Okoye P, Brooke B, Hunt R, Coetzee M (2007) Relative developmental and reproductive fitness associated with pyrethroid resistance in the major southern African malaria vector, *Anopheles funestus*. Bulletin of Entomological Research 97: 599-605.

42. Rivero A, Vézilier J, Weill M, Read AF, Gandon S (2010) Insecticide Control of Vector-Borne Diseases: When Is Insecticide Resistance a Problem? PLoS Pathog 6.

43. Saeaue L, Phumala Morales N, Komalamisra N, Vargas RM (2011) Antioxidative systems defense against oxidative stress induced by blood meal in *Aedes aegypti.* Southeast Asian Journal of Tropical Medicineand Public Health 42: 542.

44. Graça-Souza AV, Maya-Monteiro C, Paiva-Silva GO, Braz GR, Paes MC, et al. (2006) Adaptations against heme toxicity in blood-feeding arthropods. Insect biochemistry and molecular biology 36: 322-335.

45. Telang A, Wells MA (2004) The effect of larval and adult nutrition on successful autogenous egg production by a mosquito. Journal of Insect Physiology 50: 677-685.

46. Edman JD, Scott TW (1987) Host defensive behaviour and the feeding success of mosquitoes. International Journal of Tropical Insect Science 8: 617-622.

47. Calvo E, Dao A, Pham VM, Ribeiro JMC (2007) An insight into the sialome of *Anopheles funestus* reveals an emerging pattern in anopheline salivary protein families. Insect Biochem Mol Biol 37: 164-175.

48. Calvo E, Mans BJ, Andersen JF, Ribeiro JM (2006) Function and evolution of a mosquito salivary protein family. J Biol Chem 281.

49. Elanga-Ndille E, Nouage L, Binyang A, Assatse T, Tene-Fossog B, et al. (2019) Overexpression of two members of D7 salivary genes family is associated with pyrethroid resistance in the malaria vector *Anopheles funestus ss* but not in *Anopheles gambiae* in Cameroon. Genes 10: 211.

50. Riveron JM, Ibrahim SS, Chanda E, Mzilahowa T, Cuamba N, et al. (2014) The highly polymorphic *CYP6M7* cytochrome P450 gene partners with the directionally selected CYP6P9a and *CYP6P9b* genes to expand the pyrethroid resistance front in the malaria vector *Anopheles funestus* in Africa. BMC genomics 15: 817.

51. Samb B, Konate L, Irving H, Riveron JM, Dia I, et al. (2016) Investigating molecular basis of lambda-cyhalothrin resistance in an *Anopheles funestus* population from Senegal. Parasites & vectors 9: 449.

52. Wilding C, Weetman D, Rippon E, Steen K, Mawejje H, et al. (2015) Parallel evolution or purifying selection, not introgression, explains similarity in the pyrethroid detoxification linked GSTE4 of *Anopheles gambiae* and *An. arabiensis*. Molecular genetics and genomics 290: 201-215.

53. Wilding CS, Weetman D, Rippon E, Steen K, Mawejje H, et al. (2015) Parallel evolution or purifying selection, not introgression, explains similarity in the pyrethroid detoxification linked *GSTE4* of *Anopheles gambiae* and *An. arabiensis.* Molecular genetics and genomics 290: 201-215.

Figures titles

Fig1. Effect of metabolic resistance on *An. funestus* mosquito weight. Distribution of genotypes of *L119F-GSTe2* (A) and *CYP6P9a*-R (B) markers according to the weight.

Fig2. Association between resistance markers and bloodfeeding. Distribution of *L119F-GSTe2* (A) and *CYP6P9a-R* (B) genotypes between blood-fed and unfed *An. funestus* mosquitoes.

Fig3. Influence of metabolic resistance on blood meal size of *An. funestus* mosquitoes. Effect of *L119F-GSTe2* (A) and *CYP6P9a*-R (B).

Fig4. Comparative expression of salivary genes between CYP6P9a genotypes. Expression level of AAPP and some members of D7 family genes in CYP6P9a-RR and CYP6P9a-RS mosquitoes in comparison with *CYP6P9a* susceptible mosquitoes. The normalized relative expression of each gene against two housekeeping genes (RSP7 and Actin) is represented on the vertical axis. Letters a, b, c, d, e indicates the absence of significant difference in the expression level of each gene between the three types of mosquitoes.

Figure 1

Click here to access/download;Figure;Fig_1.tif

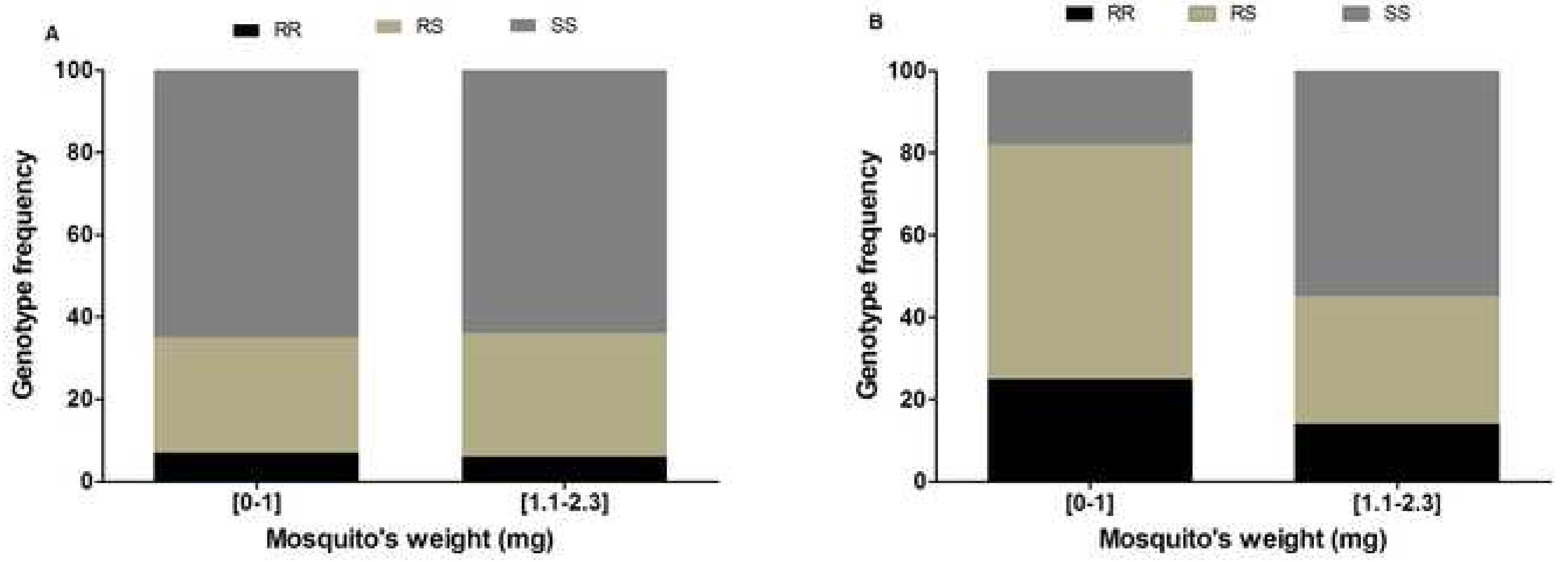

Figure 2

Click here to access/download;Figure;Fig_2.tif

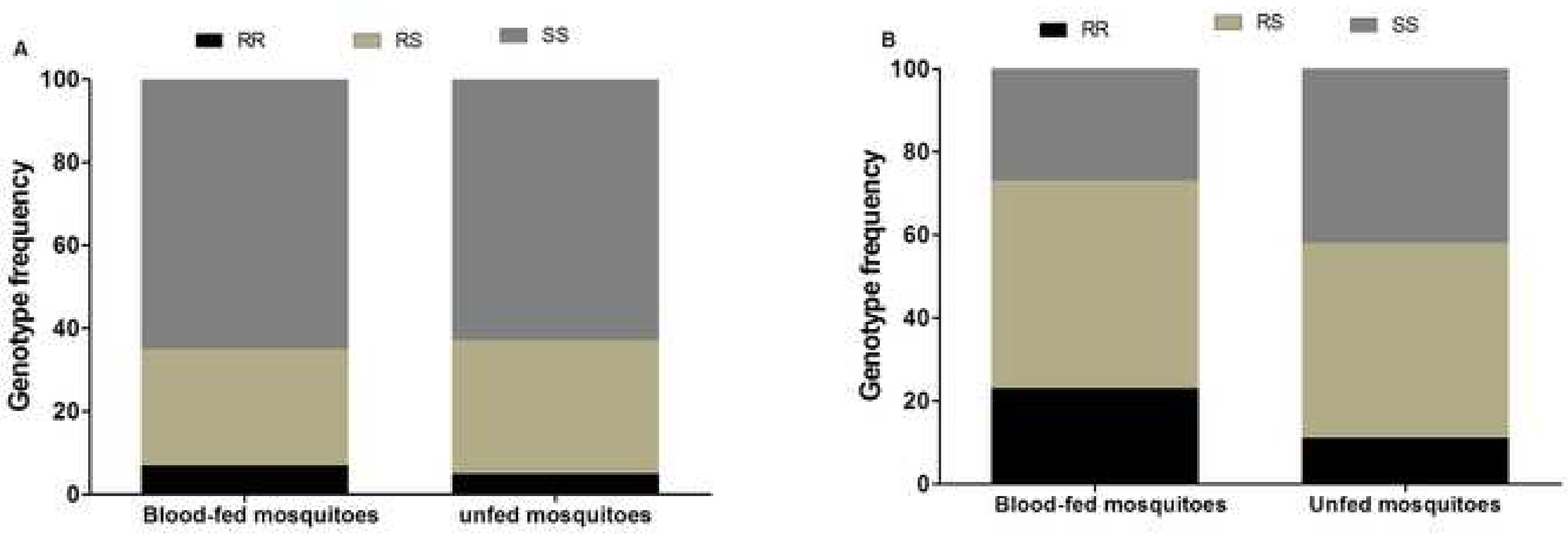

Figure 3

Click here to access/download;Figure;Fig_3.tif

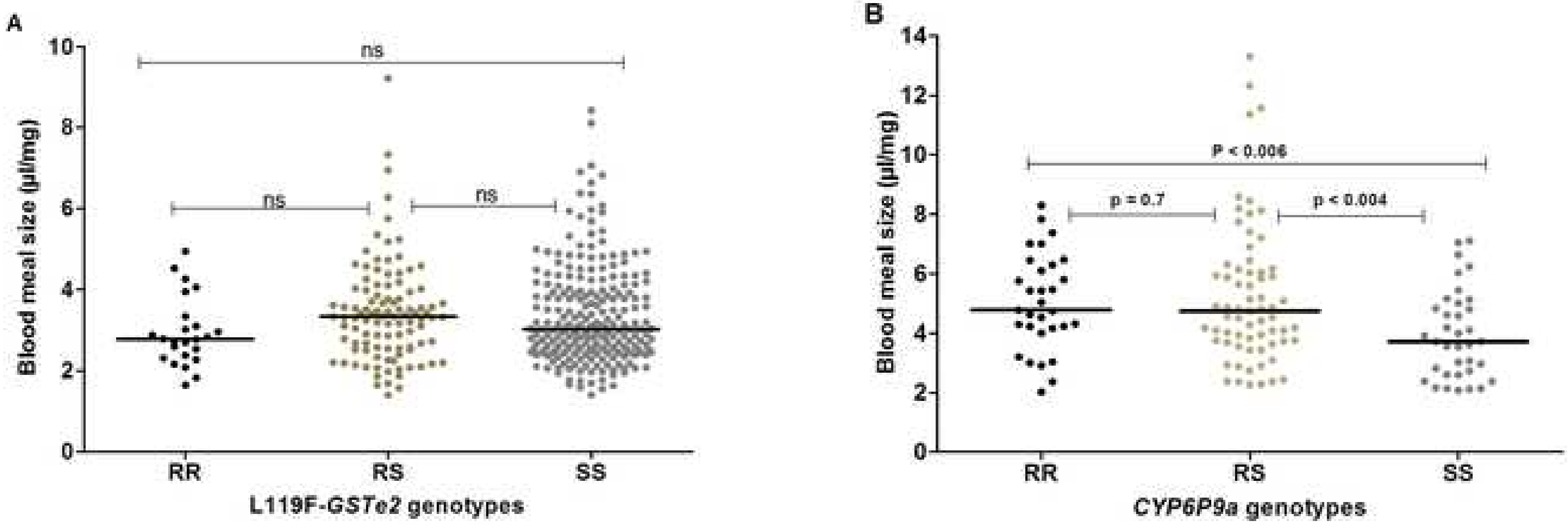

Click here to access/download;Figure;Fig_4.tif

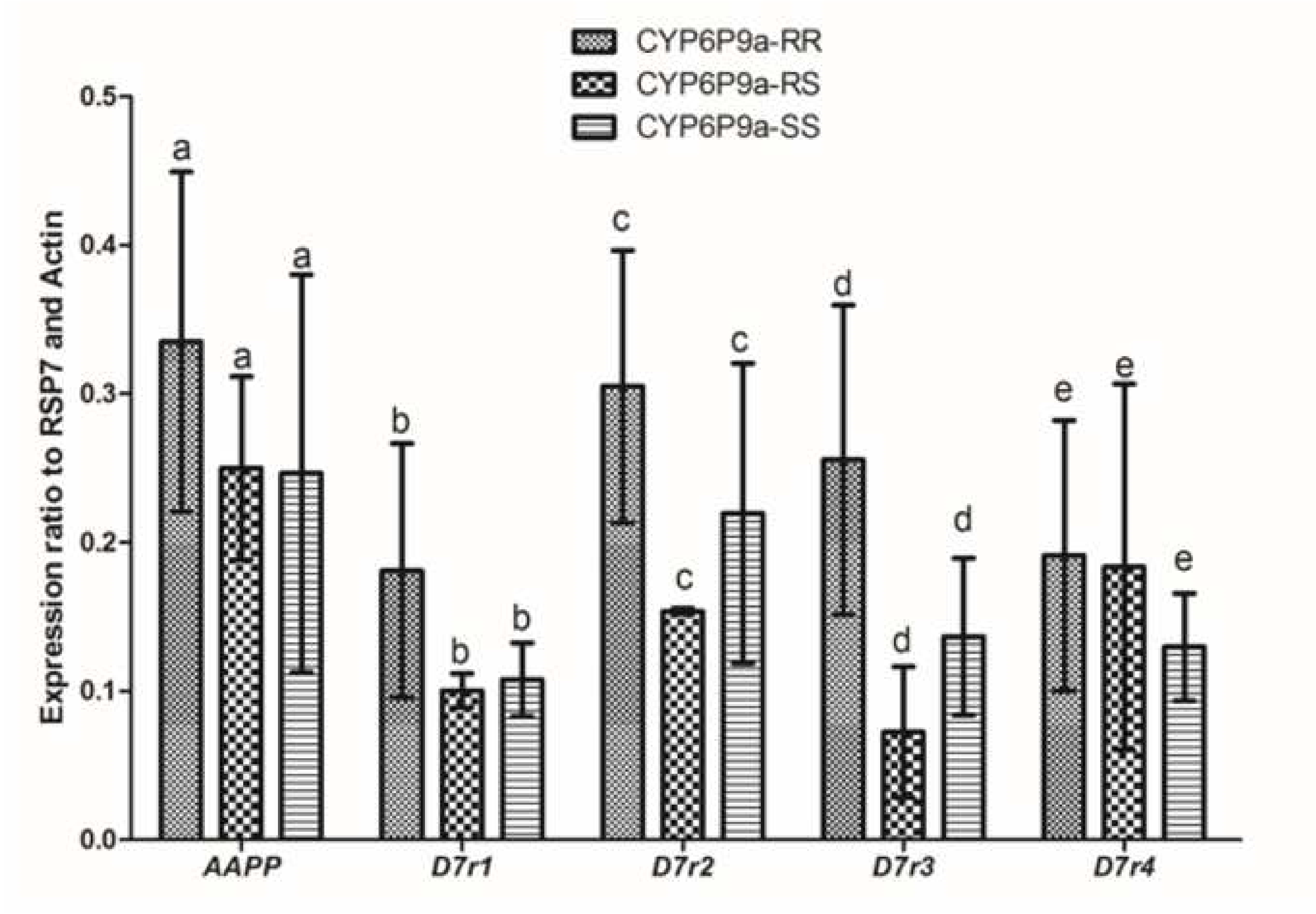

Influence of GST- and P450-based metabolic resistance to pyrethroids on blood feeding in the major African malaria vector *Anopheles funestus*

Formatted: Font: 18 pt

Formatted: Font: 14 pt

Lynda NOUAGE[1,2¥*], Emmanuel ELANGA-NDILLE[1¥*], Achille BINYANG[1,2], Magellan TCHOUAKUI[1,2], Tatiane ATSATSE[1,2], Cyrille NDO[3, 4,] , Sévilor KEKEUNOU[2], Charles S. WONDJI[1,5]

[1]Departement of Medical Entomology, Centre for Research in Infectious Diseases (CRID) P.O. BOX 13591, Yaoundé, Cameroon

[2]Department of Animal Biology and Physiology, Faculty of Science, University of Yaoundé 1, P.O. Box 812, Yaoundé, Cameroon

[3]Departement of Parasitology and Microbiology, Centre for Research in Infectious Diseases (CRID) P.O. BOX 13591, Yaoundé, Cameroon

[4]Department of Biological Sciences, Faculty of Medicine and Pharmaceutical Sciences, University of Douala, P.O. Box 24157, Douala, Cameroon.

[5]Department of Vector Biology, Liverpool School of Tropical Medicine, Pembroke Place, Liverpool L3 5QA, UK

¥: These authors contributed equally to this work

*: Corresponding authors: Emmanuel ELANGA-NDILLE (emmsdille@yahoo.fr; emmanuel.elanga@crid-cam.net) and Lynda NOUAGE (lnouage@gmail.com)

Field Code Changed

Field Code Changed

Field Code Changed

Abstract

Insecticide resistance genes are often associated with pleiotropic effects on various mosquito life-history traits. However, very little information is available on the impact of insecticide resistance on blood feeding process in mosquitoes. Here, using two recently detected DNA-based metabolic markers in the major malaria vector, *An. funestus*, we investigated how metabolic resistance genes could affect the blood meal intake.

After allowing both the field F1 and lab F8 *Anopheles funestus* strains to feed on the human arm for 30 minutes, we assessed the association between key parameters of blood meal process including, probing time, feeding duration, blood feeding success, blood meal size, and markers of glutathione S-transferase (*L119F-GSTe2*) and cytochrome P450 (*CYP6P9a_R*) - mediated metabolic resistance. None of the parameters of blood meal process was associated with *L119F-GSTe2* genotypes. ~~In~~ By contrast, for *CYP6P9a*_R, ~~homozygote~~ homozygous resistant mosquitoes were significantly more able to blood-feed than ~~homozygote~~ homozygous susceptible (OR = 3.3; CI 95%: 1.4-7.7; P =0.01) mosquitoes. Moreover, the volume of blood meal ingested by CYP6P9a-SS mosquitoes was lower than that of CYP6P9a-RS (P<0.004) and of CYP6P9a-RR (P<0.006). This suggests that *CYP6P9a* gene ~~affects~~is linked with the feeding success and blood meal size of *An. funestus*. However, no correlation was found in the expression of *CYP6P9a* and that of genes encoding for salivary proteins involved in blood meal process.

This study suggests that P450-based metabolic resistance may ~~increase~~ influence the blood feeding ~~ability~~ process of *Anopheles funestus* mosquito and ~~by~~ consequencetly its ability to transmit malaria ~~vectors and potentially impacting their vectorial capacity.~~ parasites.

Keywords: Malaria, Mosquito, *Anopheles funestus*, Insecticide resistance, Metabolic resistance, GST, P450, Blood meal intake

Formatted: Font: 18 pt

Formatted: Indent: First line: 0.49"

Formatted: Font: Italic

Introduction

Formatted: Font: 18 pt

Malaria remains a major public health scourge in sub-Sahara Africa despite significant progress made since the 2000s in reducing its burden [1]. This disease is caused by a *Plasmodium* parasite transmitted by *Anopheles* mosquito species while taking a blood meal ~~on~~ from humans. Blood feeding~~,~~ is essential for female mosquito's fecundity [2]~~,~~ as *Anopheles* species like all anautogenous female mosquitoes, require a blood meal to obtain amino acids needed to synthesize yolk proteins for eggs maturation [3,4].

Formatted: Indent: First line: 0.49"

Formatted: Font: Italic

~~Mosquito's blood~~The blood feeding success of mosquitoes ~~feeding success~~ is facilitated by the ~~pharmacological~~ biochemical proprieties of salivary gland proteins [5]. Indeed, some salivary proteins such as anopheline antiplatelet protein (AAPP), apyrase, gambiae S~~s~~alivary ~~g~~Gland protein ~~1 like~~ 6 (gSG6) ~~a~~and members of D7 family have been identified as vasodilators, anti-coagulants and inhibitors of platelet aggregation allowing mosquitoes to overcome host haemostatic mechanisms and to have a successful blood meal [5-8].

Mosquito~~'s~~ fecundity was shown to vary by source and size of the blood meal with a difference of these two parameters resulting in significant variations of the number of eggs laid by each female mosquito [4,8]. It has been shown that the number of eggs laid per female is positively associated to the amount of blood ingested as larger blood meals resulted in an increase of the number of females that developed eggs and the number of eggs per female [9,10]. The volume of blood taken by a mosquito could be affected by a range of intrinsic (host immunity) and extrinsic factors including ambient temperatures, mosquito age, parity status, gonotrophic cycle, blood feeding history and infection status [10]. More recently, it was reported that exposure to pyrethroids could also significantly influence the blood meal the process of taking

a blood meal process and the blood meal volume ingested by Kdr-resistant *Anopheles gambiae* mosquito females [11].

Pyrethroids (PY) are the insecticide class mostly used in the last two decades through ITNs and IRS strategies to control malaria transmission [12]. Unfortunately, the widespread use of these insecticides has favoured the development of resistance in malaria vector species [13,14]. Resistance to pyrethroids involves two main mechanisms: (i) metabolic resistance, due to the increase expression level of detoxifying enzymes, belonging to three families: the cytochrome P450 monooxygenases, the glutathione S-transferases and the carboxylesterases; and (ii) target-site resistance due to mutations in the voltage sodium channels known as knock-down (kdr) mutations [15,16]. Although resistance mechanisms help mosquitoes to survive under continuous insecticide pressure, these actions are costly and may negatively affect mosquito's fitness including body size, adult longevity, larval development time, fecundity, fertility, mating competitiveness and blood feeding capability [17-19]. For target-site resistance, a decreased longevity and an increased larval development time have been reported in *kdr*-pyrethroid-resistant mosquitoes [20,21]. Moreover, a recent study suggested that kdr-based resistance could impact blood feeding with heterozygote (*kdr*-RS) and susceptible (*kdr*-SS) mosquitoes taking higher blood volume than homozygote (*kdr*-RR) resistant individuals [11]. In some cases, resistant mosquitoes displayed a significant advantage compared to their susceptible counterparts as shown recently for female longevity [22] and vectorial capacity [23]. In contrastHowever, little is known on the impact of metabolic resistance as DNA-based markers were not previously available for this mechanism; thereby limiting the ability to investigate its physiological impact on the blood feeding process in mosquitoes. However, taking advantage of the identification of the first DNA-based metabolic marker in *An. funestus* mosquito, one study reported that a GST-based metabolic resistance caused by a leucine to phenylalanine amino acid change at codon 119 in the glutathione S-transferase epsilon 2

Formatted: English (United Kingdom)

(L119F-GSTe2) [24], has a detrimental impact on *An. funestus* fitness. The authors reported that ~~s as~~ field-resistant mosquitoes exhibited a reduced fecundity and slower larval development but an increased adult longevity [22]. On the other hand, a new DNA-based assay was recently designed for cytochrome P450-mediated resistance (the *CYP6P9a*-R) in *An. funestus*. This marker showed that mosquitoes carrying this P450-resistant allele survived and succeeded in blood feeding more often than did susceptible mosquitoes when exposed to insecticide-treated nets [25]. The design of assays for both GST- and P450-based resistance now offers a great opportunity to explore how the blood feeding process is influenced by metabolic resistance mechanism in malaria vectors and further assess how resistance may impact the vectorial capacity of mosquitoes to transmit malaria in the natural environment.

Here, we investigated the effect of metabolic resistance to pyrethroids on the blood feeding process in *An. funestus*, using the two DNA-based metabolic resistance markers: *L119F-GSTe2* and *CYP6P9a*-R [24,25]. Specifically, we assessed the association between the genotypes of these metabolic resistance markers and key parameters of blood feeding including mosquito probing time, feeding duration and the blood meal size.

Material and Methods

Formatted: Font: 18 pt

Mosquito collection and rearing

Formatted: Font: 16 pt

Experiments were carried out using both field and lab strains of *An. funestus*. Field mosquitoes (F_1) were generated from indoor resting female (F0) collected in Mibellon (6°46’N, 11^{0} 70’E), a village located in a rural area of the savanna-forest region in Cameroon, Central Africa where the *L119F-GSTe2* has been reported [26]. Blood-fed field collected females were kept in paper cups and transported to the insectary of the Centre for Research in Infectious

Formatted: Indent: First line: 0.49"

Diseases (CRID) in Yaoundé where they were kept for 4–5 days until they became fully gravid and were then induced to lay eggs using the forced eggs-laying method [27]. The eggs were placed in paper cups containing water to hatch, after which the larvae were transferred in to trays and reared to adults. To assess the effect of *CYP6P9a* marker, F_8 progenies were generated from ~~reciprocal~~ crosses established between the pyrethroid susceptible laboratory strain (FANG) and the resistant (FUMOZ-R) lab strain. These two *An. funestus* lab strains were colonized from mosquitoes collected in Southern Africa region. FUMOZ is a pyrethroid resistant strain established in the insectary from wild-caught *An. funestus* mosquito species from southern Mozambique [28]. The p~~-~~revious study reported that the over-expression of two duplicated P450 genes, *CYP6P9a* and *CYP6P9b*, constitute the main mechanism driving pyrethroid resistance in this strain [29,30] for which the *119F-GSTe2* allele is absent [24]. The FANG strain is completely susceptible to pyrethroids colonized from Calueque in southern Angola [28].

Blood feeding experiments and blood meal size quantification

Blood feeding process~~:~~

Since blood meal volume has previously been reported to correlate with mosquito size [2], individuals used for blood feeding experiments were firstly starved for 24h then grouped according to their size. Mosquito size was determined by weighing (using an analytical micro-scale, SARTORIUS, Goettingen, Germany~~-~~). ~~-~~E~~e~~ach starved individual (adult females aged 3-7 days) ~~starved for 24h~~ was~~and~~ immobilized by chilling ~~them~~ for 2 minutes at 5°C. Each mosquito was then placed in paper cups covered with black sheet for about an hour before given a blood meal. In order to evaluate the association between metabolic resistant markers on blood meal size, m~~M~~osquitoes were allowed to bite for 30 min on the bare forearm of a single human volunteer ~~after informed consent~~ and then genotyped for L119F-GSTe2 and CYP6P9a.~~.~~

Formatted: Font: 16 pt

Formatted: Font: 14 pt, Not Italic

Formatted: Indent: First line: 0.49"

Following to the protocol for which we obtained an ethical clearance was delivered to us by the Cameroonian national ethical committee, human volunteer was recruited among technicians working at the insectary of CRID and who by consequence are frequently exposed to mosquitoes. An informed consent was obtained after explanation of the purpose of the study.

The duration of probing and blood feeding was assessed using a batch of 120 F_1 female field-collected mosquitoes. For this purpose, mosquitoes were individually transferred in polystyrene plastic cups covered with netting. They were allowed to rest for 15 min before observations began. During the blood intake, each mosquito was filmed with a Digital HD Video Camera (Canon PC2154, Canon INC, Japan) placed beside the plastic cup. At the end of the time allowed for feeding, the film for each mosquito was analysed and the parameters such as probing time (defined as the time taken from initial insertion of the mouthparts in the skin until the initial engorgement of blood) [5] and total feeding duration, were recorded, using a digital timer. Due to the low density of female mosquitoes obtained at F_8 generation from ~~reciprocal~~ crosses of the lab strain mosquitoes, experiments to estimate the probing and the feeding duration of this strain were not investigated.

To determine the blood meal size for both strains, batches of 25 mosquitoes grouped according to their weight were allowed to bite on a human arm. In this case, neither the probing time nor the feeding duration was recorded. After the trial, the whole abdomen of successfully fed mosquitoes (evident by red-coloration engorgement of the abdomen) was extracted and stored in an individual 1.5 ml microtube at – 20°C to measure the blood meal size. The rest of the carcasses as well as unfed mosquitoes were kept individually in a microtube containing RNA-later and stored at -20°C.

Blood meal size quantification

To compare the volume of blood ingested between resistant and susceptible mosquitoes, t~~T~~he volume of blood ingested by each mosquito was determined by quantifying the haemoglobin amount, as previously described [31]. Briefly, abdomens of blood fed mosquitoes were homogenized in 0.5 ml of Drabkin's reagent (containing 1.0g of sodium bicarbonate, 0.1g potassium carbonate, 0.05g potassium cyanide, 0.2g potassium ferricyanide all diluted in 1L of distilled water). This reagents ~~which~~ converts the haemoglobin into haemoglobin cyanide (HiCN). After 20 minutes at room temperature and the addition of 0.5 ml of chloroform solution, samples were centrifuged at 5600 rpm (3512 rcf) for 5 min. The aqueous supernatant containing HiCN was transferred in a new 1.5 ml microtube. An aliquot of 200µl from each sample was transferred to a microplate and the optical density (OD) read at a wavelength of 620nm in a spectrophotometer (EZ Read 400, biochrom, Cambridge, UK). OD for each sample were read in duplicate and the average value between the two replicates was considered as OD value of the sample. In parallel, OD read on various amounts of human volunteer blood added to Drabkin's reagent in individual microtubes were used as control to generate calibration curves and the regression line used to assess the relationship between OD and blood volume. For each sample, the blood meal size was estimated according to the weight by dividing the blood volume estimated using the regression line by the average weigh of each batch of mosquitoes constituted after the weighing. The blood meal size was then expressed in µL of blood per mg of weight.

Formatted: Font: 16 pt

Formatted: Indent: First line: 0.49"

Molecular species identification

To determine the species composition of *An. funestus* group among the samples, g~~G~~enomic DNA (gDNA) was extracted from both blood-fed and unfed mosquitoes using the Livak protocol [32]. Instead of using the whole body as done for unfed mosquitoes, DNA was extracted from the carcasses of fed mosquitoes after removing the abdomen for blood volume quantification. The concentration and purity of the extracted gDNA were subsequently determined using a NanoDrop™ spectrophotometer (Thermo Scientific, Wilmington, USA) before storage at −20 °C. An aliquot of gDNA extracted from field-collected strain was used for molecular identification by a polymerase chain reaction [33]. ~~to determine species composition of *An. funestus* group among the samples.~~

Genotyping of *L119F-GSTe2* mutation in field-collected strain

The *L119F-GSTe2* mutation was genotyped using gDNA extracted from carcasses of field-collected strains following an allele specific PCR diagnostic assay previously described [22]. The primers sequences are given in table S1. PCR was performed in Gene Touch thermal cycler (Model TC-E-48DA, Hangzhou, 310053, China), in a reaction volume of 15 µl using 10 µM of each primer, 10X Kapa Taq buffer A, 0.2 mM dNTPs, 1.5 mM MgCl2, 1U Kapa Taq (Kapa Biosystems, Wilmington, MA, USA) and 1µl of genomic DNA as template. The cycle parameters were: 1 cycle at 95 °C for 2 min; 30 cycles of 94 °C for 30 s, 58 °C for 30 s, 72 °C for 1 min and then a final extension at 72 °C for 10 min. The PCR products were size separated on a 2 % agarose gel stained with Midori Green Advance DNA Stain (Nippon genetics Europe GmbH) and visualised using a gel imaging system~~PCR products were separated on 2% agarose gel by electrophoresis~~. The size of the diagnostic band was 523 bp for homozygous resistant

Formatted: Font: 16 pt

Formatted: Indent: First line: 0.49"

Formatted: Font: 16 pt

Formatted: Indent: First line: 0.49"

(RR) and 312 bp for homozygous susceptible (SS), while heterozygous (RS) showed the two bands.

Genotyping of *CYP6P9a*-R allele in lab strain mosquitoes

The *CYP6P9a* resistance marker was genotyped using the protocol recently designed by [25]. A PCR-RFLP were carried out using gDNA extracted from the carcasses of F_8 generation individuals obtained from the reciprocal crosses between FANG and FUMOZ strains used for blood feeding. Briefly, a partial *CYP6P9a* upstream region was amplified in a final volume of 15µl PCR mixture containing ~~1.5µl of~~ 10X Kapa Taq buffer A (Kapa Biosystems, Wilmington, MA, USA), ~~0.12µl of~~ 5 U/µl KAPA taq, ~~0.12µl of~~ 25µM dNTP, ~~0.75µl of~~ 25µM MgCl2, ~~0.51µl~~10 mM of each primer, 10.49µl of dH2O and 1µl of genomic DNA. The PCR cycle parameters were as follows~~ed~~: the initial denaturation step at 95°C for 5 minutes followed~~ed~~ by 35 cycles of 94°C for 30 seconds, 58°C for 30 seconds and 72°C for 45 seconds and a final extension step of 72°C for 10 minutes. The PCR products were size separated on a 1.5 % agarose gel stained with Midori Green Advance DNA Stain (Nippon genetics Europe GmbH) and visualised using a gel imaging system to confirm the product size (450bp). Then, the PCR product was incubated at 65°C for 2 hours. This was done in 0.2ml PCR strip tubes using 5µl of PCR product, 1µl of cutSmart buffer, 0.2µl of 2 units of Taq1 enzyme (New England Biolabs, catalog: ER0672) and 3.8µl of dH20. Size separation was done on a 2.0% agarose gel stained with Midori Green Advance DNA Stain at 100V for 30 minutes. The gel was visualised using the gel imaging system.

Formatted: Font: 16 pt

Formatted: Indent: First line: 0.49"

Gene expression profiling of major salivary genes encoding proteins involved in blood meal process.

The expression profiles of a set of salivary genes encoding for proteins involved in blood meal process was compared between CYP6P9a-RR, CYP6P9a-RS and CYP6P9a-SS *An. funestus* mosquitoes. For each gene, two pairs of exon-spanning primers was designed for each gene using Primer3 online software (v4.0.0; http://bioinfo.ut.ee/primer3/) and only primers with PCR efficiency between 90 and 110% determined using a cDNA dilution series obtained from a single sample, were used for qPCR analysis. Taking into account this criteria of efficiency, only the AAPP and four members of D7 family genes (D7r1, D7r2, D7r3, and D7r4) were used for this analysis. Primers are listed in Table S1. Total RNA was extracted from three batches of 10 whole females of 3–5 days old from CYP6P9a-RR, CYP6P9a-RS and CYP6P9a-SS mosquitoes. RNA was isolated using the RNAeasy Mini kit (Qiagen) according to the manufacturer's instructions. The RNA quantity was assessed using a NanoDrop ND1000 spectrophotometer (Thermo Fisher) and 1µg from each of the three biological replicates for each batch of mosquitoes was used as a template for cDNA synthesis using the SuperScript III (Invitrogen, Waltham, Massachusetts, USA) with oligo-dT20 and RNase H, following the manufacturer's instructions. The qPCR assays were carried out in a MX 3005 real-time PCR system (Agilent, Santa Clara, CA 95051, United States) using Brilliant III Ultra-Fast SYBR Green qPCR Master Mix (Agilent). A total of 10 ng of cDNA from each sample was used as template in a three-step program involving a denaturation at 95 °C for 3 min followed by 40 cycles of 10 s at 95 °C and 10 s at 60 °C and a last step of 1 min at 95 °C, 30 s at 55 °C, and 30 s at 95 °C. The relative expression and fold-change of each target gene in CYP6P9a-RR and CYP6P9a-RS relative to CYP6P9a-SS was calculated according to the $2^{-\Delta\Delta CT}$ method incorporating PCR efficiency after normalization with the housekeeping RSP7 ribosomal

Formatted: Font: 16 pt

Formatted: Indent: First line: 0.49"

Field Code Changed

protein S7 (VectorBase ID: AFUN007153;) and the actin 5C (vectorBase ID: AFUN006819) genes for *An. funestus*.

Statistical analysis

All analyses were conducted using GraphPad Prism version 7.00 software. We estimated a Fisher's exact probability test and the odds-ratio of *L119F-GSTe2* and *CYP6P9a* genotypes (homozygous resistant = RR, heterozygote resistant=RS and homozygous resistant =SS) and both susceptible (S) and resistant (R) alleles. This allowed ~~us to~~to the ~~-~~assessment of ~~the~~ association between: a) insecticide resistance and mosquito's weight by comparing the proportions of the genotypes of both genes in each group established after weighing; b) blood feeding success and insecticide resistance by comparing the proportion of each genotype in both fed and unfed mosquitoes. The feeding duration was grouped into four intervals with 1 minute (60s) amplitude for each interval.~~After arbitrary regrouping the time into four different intervals with same amplitude, t~~The duration of probing and feeding was analysed by comparing the proportion of mosquitoes (for *L119F-GSTe2* and *CYP6P9a*) with different genotypes in each defined intervals using~~with~~ chi-square test.~~ by comparing the proportion of *L119F-GSTe2* and *CYP6P9a* genotypes in each defined time interval~~. After estimating the median of weighted blood meal for each genotype, Kruskal-Wallis and Mann-Whitney tests were used to compare the differences between more than two groups and between two groups, respectively as Shapiro welch test showed non normal distribution.~~.~~

Results

Metabolic resistance genes and *An. funestus* mosquito's weight

Formatted: Font: 16 pt

Formatted: Indent: First line: 0.49"

Formatted: Font: 18 pt

Formatted: Font: 18 pt, English (United Kingdom)

Formatted: Font: 16 pt, Font color: Auto

Formatted: Font: 16 pt

Formatted: Font: 16 pt, Not Bold

A total of 1,200 and 273 female mosquitoes were weightted, respectively for field strain (F_1 generation) and lab strain (F_8 generation). The mean weight of a mosquito was 0.9 ± 0.010 mg (minimum = 0.2 mg; maximum = 2.3mg) and 0.89 ± 0.016 mg (minimum = 0.2 mg; maximum = 1.7mg) for field and lab strain respectively. No significant difference was found between the mean weights of two strains. For all the analyses, we arbitrarily grouped mosquitoes according to their weight values, into two different classes as followed: [0 - 1.0] mg and [1.1 - 2.3] mg. Analysis of the distribution of *L119F-GSTe2* mutation genotypes in each class of field strain mosquitoes showed no association between the mosquito's weight and *L119F-GSTe2* genotypes (χ^2 = 0.15; *p* = 0.9; OR =1.2, 95%CI: 0.3742 - 4.176, for RR *vs* RS; OR=1.1, 95%CI: 0.3659 - 3.606 for RR *vs* SS; OR = 0.9, 95%CI: 0.4943 - 1.709 for RS *vs* SS) (Figure 1 and Table 1). This absence of correlation between the *L119F-GSTe2* genotypes and the weight of mosquitoes was confirmed at the allele level (OR=1; 95%: CI: 0.5–2.0; *p* =0.5) showing that the L119F mutation may not impact associated with the weight of this *An. funestus* field population (Table 1). In contrast, a significant association was observed between *CYP6P9a* genotypes and the weight of mosquito (χ^2= 29.54, p<0. 0001). Indeed, proportions of RR and RS genotypes were higher than that of SS in the lowest weight class, whereas, for larger weight, mosquitoes with SS genotype were more abundant (67.2%). This association is further supported by odds ratio estimates showing that proportions of homozygote resistant (RR) (OR=5.4; CI 95%: 2.3-12.7; *p*<0.0001) and heterozygote (RS) (OR=5.6; CI 95%: 2.8-11.1; *p*<0.0001) mosquitoes are significantly higher in lowest weight class than the larger one when compared to homozygote susceptible mosquitoes (Table 1). Overall, mosquitoes harbouring the *CYP6P9a*-S susceptible allele displayed higher weight compared to those with the *CYP6P9a*-R resistant allele (OR=2.8; CI 95%: 1.5–5.0; p =0.0003 (Table 1) suggesting that over-expression of the *CYP6P9a* gene is reducing the weight of pyrethroid resistant *An. funestus* mosquitoes.

Table 1: level of association of *L119F-GSTe2* and *CYP6P9a*-R genotypes with mosquito weight by comparing low (0-1.0mg) and high (1-2.4mg) weight samples.

	L119F-GSTe2		***CYP6P9a-R***	
**Genotypes**	**Odds ratio**	***p*-value**	**Odds ratio**	***p*-value**
**RR vs SS**	1.1 (0.4-3.6)	0.5	5.4 (2.3-12.7)	< 0.0001
**RS vs SS**	0.9 (0.5-1.7)	0.4	5.6 (2.8-11.1)	< 0.0001
**RR vs RS**	1.2 (0.4-4.1)	0.5	1.0 (0.5-2.3)	0.5
**S vs R**	1 (0.5-2.0)	0.5	2.8 (1.5-5.0)	0.0003

SS: homozygote susceptible; RR: homozygote resistant; RS: heterozygote; * significant difference p < 0.05.

~~Impact~~ Influence of *L119F-GSTe2* and *CYP6P9a* mutations on *An. funestus* blood feeding success

***L119F-GSTe2*:** out of the 1,200 individuals from field strain mosquitoes that were allowed to take a blood meal, 457 (39.6%) successfully fed whereas 743 did not. Among blood-fed mosquitoes, a total of 360 were successfully genotyped and 7% (24/360) were ~~homozygote~~ homozygous resistant (RR), 28% (103/360) were ~~heterozygotes~~ heterozygous resistant (RS) and 65% (233/360) were ~~homozygotes~~ homozygous susceptible (SS) (Figure 2a). On the other hand, out of the 300 unfed mosquitoes randomly selected for genotyping, 5% (15/300), 32% (62/300) and 63% (189/300), were ~~homozygote~~ homozygous resistant, heterozygotes and homozygote susceptible, respectively (Figure 2a). However, the distribution of L119F genotypes was not statistically different between blood-fed and unfed mosquitoes (χ^2=0.63, *p*=0.7). Furthermore, estimation of odds ratio. (OR=1; CI 95%: 0.5–2.0; *p* = 0.6) showed overall that mosquitoes bearing the 119F-R resistant allele have the same chance to have a

Formatted: Font: Bold

Formatted Table

Formatted: Font: 16 pt

Formatted: Indent: First line: 0.49"

successful blood feeding than those with the 119F-S susceptible alleles (Table 2). This suggests that the ability to take blood is not ~~impacted~~ associated with~~by~~ the *L119F-GSTe2* mutation in *An. funestus.*

Table 2: Assessment of the association of *L119F-GSTe2* and *CYP6P9a-R* mutations with *An. funestus* mosquito blood feeding

	L119F-GSTe2		***CYP6P9a-R***	
**Genotypes**	**Odds ratio**	***p*-value**	**Odds ratio**	***p*-value**
**RR vs SS**	0.7 (0.2-2.4)	0.4	3.3 (1.4-7.7)	0.01
RS vs SS	1.1 (0.62-2.1)	0.4	1.7 (0.9-3.1)	0.1
RR vs RS	0.6 (0.2-2.3)	0.3	1.9 (0.9-4.4)	0.1
R vs S	1 (0.5-2.0)	0.6	1.8 (1.1-3.2)	0.04

SS: homozygote susceptible; RR: homozygote resistant; RS: heterozygote; * significant difference p < 0.05.

CYP6P9a-R: Among a total of 273 mosquitoes that were offered a blood meal 140 successfully fed (51.3%) whereas, 133 did not. Out of the 140 mosquitoes that blood-fed, 134 were successfully genotyped for *CYP6P9a*-R allele revealing that 23% (31/134), 50% (67/134) and 27% (36/134) were homozygote resistant CYP6P9a-RR, heterozygotes CYP6P9a-RS and homozygote susceptible CYP6P9a-SS, respectively (Figure 2b). Among the unfed mosquitoes, 11.3% (15/133) were homozygote resistant CYP6P9a-RR, 47.4% (63/133) heterozygotes and 41.3% (55/133) were homozygote susceptible CYP6P9a-SS. The estimation of odds ratio showed that homozygote resistant CYP6P9a-RR mosquitoes are significantly more able to blood feed than homozygote susceptible (OR = 3.33; CI 95%: 1.4 -7.7; *p* =0.01). No difference was observed between heterozygote and homozygote resistant CYP6P9a-RR (OR= 1.9, 95%CI: 0.9-4.4; p=0.1) neither with homozygote susceptible CYP6P9a-SS (OR= 1.7, 95%CI: 0.9-3.1;

Formatted: Font: Bold

Formatted Table

Formatted: Indent: First line: 0.49"

p=0.1) mosquitoes (Table 2). Moreover, it was overall observed that mosquitoes with the *CYP6P9a*-R resistant allele have a greater chance to blood feed than those bearing the susceptible allele (OR = 1.9; CI 95%: 1.03-3.2; *p* =0.04) (Table 2).

~~Impact~~ Association between ~~of~~ the *L119F-GSTe2* mutation ~~on~~ and probing ~~and~~/ blood feeding duration

Formatted: Font: 16 pt

Out of the 120 mosquitoes that were individually filmed to assess the influence of insecticide resistance genes on the probing and feeding duration, 7 (6.14%), 40 (35.08%) and 67 (58.77%) were genotyped as homozygous resistant 119F/F-RR, ~~heterozygotes~~ heterozygous L119F-RS and ~~homozygotes~~ homozygous susceptible L/L119, respectively. Overall, regardless of the genotype, the median value of mosquito's probing duration was 49.5 seconds (minimum = 4s and maximum = 290s). No difference was observed in the probing time of resistant mosquitoes 119F/F-RR (Median = 53 seconds) and heterozygotes L119F-RS (Median = 52s) compared to the homozygote susceptible L/L119 (Median = 52s).

Formatted: Indent: First line: 0.49"

Regarding the blood feeding duration, it was observed that the median and mean time for a mosquito to have a full blood meal was 249.5 seconds and 303 ± 181 seconds respectively, with a minimum = 68 seconds and a maximum =772 seconds. The feeding duration was longer (median=269s) in L/L119 mosquitoes compared to L119F-RS (229.5s) and 119F/F-RR (214s) but the difference was not statistically significant (*p*=0.19, Kruskal-Wallis test).

~~Impact~~ Effect of *L119F-GSTe2* and *CYP6P9a*-R mutations on the blood meal size of *An. funestus*

Formatted: Font: 16 pt

L119F-GSTe2 : From 457 individuals that took a full blood meal it was observed that the average weighted blood meal of a mosquito regardless of the *L119F-GSTe2* genotype was 3.4± 1.3 µl/mg (minimum = 1.2 µl/mg; maximum = 9.2 µl/mg). However, the weighted blood

Formatted: Indent: First line: 0.49"

meal was not significantly different (P=0.17; Kruskal-Wallis test; Figure 3a) in homozygote susceptible L119-SS (3.0µl/mg) compared to homozygote resistant L119-RR (2.8µl/mg) and heterozygote L119F-RS (3.3µl/mg) mosquitoes. This result suggests that the *L119F-GSTe2* mutation ~~may~~ is not ~~affect~~ associated with the volume of blood meal ingested by *An. funestus*.

CYP6P9a-R: The influence of the *CYP6P9a*-R mutation on the volume of blood meal taken by *An. funestus*, was assessed using the 134 blood fed mosquitoes that were successfully genotyped for *CYP6P9a*-R allele. Overall, irrespective of the genotype, the mean weighted blood volume ingested by a mosquito was 4.8 ± 2 µl/mg (minimum = 2 µl/mg; maximum = 13.3µl/mg). However, the weighted blood meal volume of CYP6P9a-SS mosquitoes (Median = 3.71µl/mg) was lower than that of CYP6P9a-RS (Median = 4.73 µl/mg) and of CYP6P9a-RR (Median = 4.78 µl/mg) (Figure 3; p<0.004 for RS vs SS and p<0.006 for RR vs SS, Mann-Whitney test). No difference in the volume of the blood meal was observed between CYP6P9a-RR and CYP6P9a-RS mosquitoes (P=0.7; Mann-Whitney test). This result suggests that the over-expression of *CYP6P9a* gene is associated with an increase of the volume of the blood meal ingested by *An. funestus*.

Expression profile of *AAPP* and *D7* family salivary genes according to *CYP6P9a*-R genotypes

Due to the association observed between the *CYP6P9a*-R genotypes and blood feeding, an attempt was made to assess whether the genotypes of this gene could ~~impact~~ be possible associated with the expression profile of key salivary genes. ~~Analysis of the~~ The expression level of AAPP and 4 members of the D7 family salivary genes (D7r1, D7r2, D7r3 and D7r4) was analysed and compared between homozygous resistant (CYP6P9a-RR), heterozygous (CYP6P9a-RS) and homozygous susceptible genotype (CYP6P9a-SS) mosquitoes. No

Formatted: Font: 16 pt

Formatted: Indent: First line: 0.49"

significant difference in the expression level of these genes was observed between the three types of mosquitoes although D7 familly genes appeared slightly over-expressed in CYP6P9a-RR and CYP6P9a-RS when compared to CYP6P9a-SS did not show a significant difference in expression in homozygote homozygous resistant (CYP6P9a-RR) and heterozygote heterozygous (CYP6P9a-RS) mosquitoes when compared to homozygote homozygous susceptible genotype (CYP6P9a-SS) (Figure 4, Table 2) with average fold-change for all these genes lower than 1.5. This result suggests that *CYP6P9a*-R genotypes do not influence the expression profile of both AAPP and D7 family genes in the salivary glands of *An. funestus* mosquitoes.

Discussion.

Formatted: Font: 18 pt

Due to the absence of markers, the impact of metabolic resistance on life traits of *Anopheles* mosquitoes has been poorly elucidated. Recently, mutations in the *GST* epsilon 2 and in the promoter region of the cytochrome P450 *CYP6P9a*, were described as robust molecular markers for tracking metabolic resistance in pyrethroids resistant populations of *An. funestus* [24,25]. Using these two key markers, this study assess the impact influencepossible association of GST- and P450-based metabolic resistance to pyrethroids on the feeding process and blood meal volume of *An. funestus*.

Formatted: Indent: First line: 0.49"

~~*Impact*~~ Association of metabolic resistance on blood feeding success

The present study revealed that *CYP6P9a* but not the *L119F-GSTe2* mutation could impact the blood feeding success of *An. funestus* mosquito as possessing the *CYP6P9a* resistant allele increased the likelihood of being successful in blood-feeding. Such selective advantage of *CYP6P9a* resistance allele was also previously reported in a semi-field study in experimental hut trial which observed that homozygous CYP6P9a-RR mosquitoes were significantly more likely to blood feed than susceptible SS [25]. This result suggests that CYP6P9a -mediated metabolic resistance might influence the ability of *An. funestus* mosquito to blood feed. In contrast, the absence of association observed here for the *L119F-GSTe2* mutation needed to be confirmed by further studies as the low sample of L119F-RR homozygous~~te~~ resistance mosquitoes might have biased our analysis. This low number of L119F-RR mosquitoes could itself be linked to unsuccessfully genotyping of this marker in approximately 20% of samples analysed in this study. This important point highlights the need for further studies to improve the optimization of the protocol used in this study for the genotyping of the L119F-GSTe2 mutation. ~~Nevertheless~~On~~In~~ the other hands, the mechanism whereby *CYP6P9a*-R resistant allele could ~~impact~~ influence mosquito feeding is unknown and was not investigated in the present study. One hypothesis to explain this association could be related to the motivation of mosquito to blood feed. In fact, it has been reported that some mosquito individuals that emerged with insufficient teneral reserves require an initial blood meal to compensate for insufficient teneral reserves rather than for egg development during their first gonotrophic cycle [34-36]. This phenomenon is mostly observed in smaller female mosquitoes that emerge with insufficient reserve [2]. Thus, we can presume that *CYP6P9a* resistant mosquitoes which were found significantly smaller than susceptible in the present study were more motivated to blood feed as they were probably the ones requiring more to compensate for their insufficient teneral reserves. However, it’s important to note that it was surprising and unusual to observed

Formatted: Font: 16 pt, Not Italic

Formatted: Indent: First line: 0.49"

CYP6Pa-SS mosquitoes bigger that CYP6Pa-RR ones since previous studies often reported larger mosquitoes tend to be more tolerant of insecticides, or that resistant phenotypes are associated with larger body sizes[37,38] (Osuwu et al: Sci Rep. 2017 Jun 16;7(1):3667, Jeanrenaud et al, PLoS One. 2019 Apr 18;14(4):e0215552). This unusual observation could be explained by the fact that, instead of using dead dried and unfed mosquito as usually done, in the present study the weight was estimated using alive fresh mosquitoes which were fed with sugar solution until 24 hours before being weighted. With this approach, mosquito's body weight may have been influenced by water and/or elements of sugar digestion that are eliminated when the mosquito is dried. This point highlight the need to perform further studies working for instance with dried mosquitoes before confirm our hypothesis about the association of teneral reserve and CYP6P9a mutation. One other approach could be to carry out calorimetric assays comparing teneral reserve between CYP6Pa-SS and CYP6Pa-RR mosquitoes. These fFurther studies would certainly be more informative investigating the impact ofon the influence of insecticide metabolic resistance on the motivation of *An. funestus* mosquito to blood feed would probably help in confirming this hypothesis.

Formatted: Pattern: Clear

Impact Influence of metabolic resistance on probing time and feeding duration

Formatted: Font: 16 pt, Not Italic

Formatted: Font: Not Italic

The influence of metabolic resistance on probing time and feeding duration was assessed in the present study only for *L119F-GSTe2* mutation. Results revealed no significant impact association of this metabolic resistance gene on the time spent by a mosquito to probe. The

Formatted: Indent: First line: 0.49"

absence of ~~impact~~association of insecticide resistance on mosquito probing time was also reported for the *knock-down* (*kdr*) resistance gene in *Anopheles gambiae* with no difference in the probing time noticed between genotypes (RR, RS and SS) after exposure to untreated and insecticide-treated net [11]. This seems to indicate that insecticide resistance might not impact the probing duration of *Anopheles* mosquito during blood feeding. However, this hypothesis must be taken with caution as, to our knowledge, and the exception of the present study as well as the one of Diop et *al*, very little information is available on the impact of insecticide resistance on the probing time during mosquito blood-feeding. In the other hand, even if the difference was not statistically significant, mosquitoes possessing an *119F-GSTe2* resistant allele (both homozygous and heterozygous) spent less time taking their blood meal than susceptible. This corroborate with observation previously made for *kdr* mutation in *An. gambiae* with lower feeding duration for homozygous resistant mosquitoes than heterozygote and homozygous susceptible [11]. The non-significant result observed may be due to the low number of resistant mosquitoes in the present study. However, from the results, it could be hypothesized that *L119F-GSTe2* mutation might confer an advantage to homozygous resistant mosquitoes as it was previously reported that rapid feeding reduces the risk to be killed by the host defensive behaviour [11,39].

~~Impact~~ Effect of metabolic resistance on blood meal volume

Formatted: Font: 16 pt, Not Italic

In ~~the present~~this study, we observed that the volume of blood ingested by a mosquito during a single blood feeding was associated with the genotype of the P450 *CYP6P9a* but not with the *L119F-GSTe2*-based metabolic resistance. This suggests that mechanisms involved in metabolic resistance to pyrethroids in *An. funestus* might influence mosquito life-traits

Formatted: Indent: First line: 0.49"

differently. However, as already discussed above, we cannot exclude that the absence of the influence observed for *L119F-GSTe2* gene might also be related to the low number of L119F-RR mosquitoes used in the present study. This latter hypothesis seems moreover reinforced by the results of previous studies showing *L119F-GSTe2* mutation [22] and *CYP6P9a*-R resistance gene [40] affecting influencing *An. funestus* fecundity in the same way. The positive association between *CYP6P9a*-R resistant allele and the volume of blood meal is in line with the work is of Okoye and collaborators reporting that pyrethroid resistance mechanism in southern African *An. funestus* cause no reduction in fitness of this mosquito ([41]Okoye et al 2007). Thus, our finding suggests that the over-expression of CYP6P9a gene might probably not compromise the volume of blood ingested of individual mosquitoes carrying the CYP6P9a-R resistant allele. Given a bit surprising knowing that activity of P450 monoxygenases as well as blood meal digestion, have been reported to generate an excess production of reactive oxygen species (ROS) increasing oxidative stress which could induce several damages in the mosquito's system that can result to death [42,43], it could have been expected to see CYP6P9a-RR mosquitoes taking lower blood to reduce negative effects of oxidative stress. This observation could certainly be explained by the ability of *Anopheles* mosquitoes to cope with oxidative damage after blood feeding by increasing the activity antioxidant activity enzymes including, Cu Zn and Mn superoxide dismutase (SOD), catalase, glutathione peroxides and thioredoxin reductase [44](Graca-Souza, A. V. et al. Adaptations against heme toxicity in blood-feeding arthropods. Insect Biochem Mol Biol 36, 322–335, https://doi.org/10.1016/j.ibmb.2006.01.009 (2006)). In fact, because the *CYP6P9a*-R resistant allele was recently reported to be negatively associated with the fecundity of *An. funestus* [40], we were expecting to see *CYP6P9a* resistant mosquitoes taking lower blood meal than susceptible to reduce negative effects of oxidative stress. This suggests that association between the *CYP6P9a*-R resistant allele and mosquito's blood meal size could be an indirect consequence of some other physiological activities. For instance,

Formatted: Font: Italic

Formatted: Font: Not Italic

Formatted: Font: Italic

Field Code Changed

because *CYP6P9a* resistant mosquitoes were significantly smaller than their susceptible counterparts, and noting that it has been demonstrated that the amount of teneral reserves is proportional to the body size of mosquito [2], we can presume that the high blood meal volume ingested by CYP6P9a-RR mosquitoes might be as a result of a need to compensate for the limited teneral reserves post emergence. In this case, the association observed here could be an indirect consequence of the negative ~~impact~~ association of *CYP6P9a*-R resistant allele recently observed on the larval development of *An. funestus* [40] resulting to a small body size, and by consequence to insufficient teneral reserves for resistant mosquitoes. Indeed, it was demonstrated that encountering a nutritional environment by *Anopheles* larvae strongly influences adult fitness-related traits such as body size and teneral metabolic reserves [2,31,45]. However, our finding did not corroborate with the positive association previously reported between the volume of ingested blood meal and mosquito body size [2]. Further studies will help elucidate the underlying reason of this correlation between *CYP6P9a* genotypes and blood meal size.

~~Influence~~ Possible association *of CYP6P9a-R* resistant allele on salivary gland genes expression

To obtain a successful blood meal, a female mosquito must balance the risk of death caused by host defensive behavior against the benefits to feed on a host species that maximize fertility [46]. Salivary components permit mosquitoes to reduce their engorgement time and increase their likelihood of survival [5]. In the present study, we assessed the level of expression of genes encoding for some salivary proteins known to be involved on blood intake process of mosquitoes such as, AAPP and members of D7 family proteins [6,47,48]. The comparative analysis of the expression level of these genes between *CYP6P9a* genotypes showed no significant difference between mosquitoes bearing the resistant allele and those with the

Formatted: Font: 16 pt, Not Italic
Formatted: Font: 16 pt
Formatted: Font: 16 pt
Formatted: Font: 16 pt, Not Italic
Formatted: Indent: First line: 0.49"

susceptible one. This result suggests that the expression of AAPP and D7 family salivary genes are not associated to the *CYP6P9a* mutation. This observation is intriguing as some salivary genes such as D7 family genes were previously reported to be over-expressed in resistant *An. funestus* mosquito compared to susceptible strain [24,49-52]. The lack of significance observed with the differential expression of genes in the present study could be explained by the fact that, our analyses in this study were performed on mosquitoes obtained after ~~reciprocal~~ crosses between two different strains and therefore sharing the same background, while other studies compared insecticide resistant field /laboratory mosquitoes and susceptible laboratory strains with different genetic background [24,49,53]. The absence of influence of the *CYP6P9a* gene on the expression level of salivary gland genes involved in the blood feeding process observed in the present study appears to indicate that the association found between this gene and the size of blood meal taken by *An. funestus* mosquito might not be related to the expression of these salivary genes encoding proteins which mediate the blood meal process.

This study revealed that GSTe2-mediated resistance ~~does~~ is not ~~affect~~ associated with the blood meal intake of *An. funestus* mosquitoes, whereas *CYP6P9a*-based resistance to pyrethroids is associated with a feeding success and a higher blood meal size. However, this influence on *Anopheles funestus* blood meal intake is not associated with differential expression of major salivary gland proteins involved in blood-feeding. Given the rapid growth of insecticide resistance, it would be interesting to study how this association could ~~impact~~ affect the fecundity and the vectorial capacity of *An. funestus* mosquitoes.

Author Contributions: E.E.N and C.S.W conceived the study; EEN, L.N, C.N SK and C.S.W designed the study; E.E.N, .L.N, A.B, T.A and M.T carried out the sample collection; L.N, A.B, and T.A reared and maintained the strain in the insectary; E.E.N, L.N A.B, and T.A performed blood feeding experiments. L.N, T.A and M.T performed the molecular analyses; E.E.N, L.N, A.B, M.T and C.N analyzed the data; E.E.N, L.N and

C.S.W wrote the manuscript. M.T C.N and S.K reviewed the manuscript. All authors approved the manuscript.

Ethical approval and consent to participate

Ethical clearance was obtained from the National Ethics Committee of Cameroon's Ministry of Public Health (N°2018/04/1000/CE/CNERSH/SP) in conformity to the WMA Declaration of Helsinki. Informed verbal consent was obtained from household owners for using their houses for mosquito collection.

Acknowledgments: We thank Dr Micheal KUSIMO and Mr Francis NKEMNGO; native speakers of English language for accepting to review and edit this paper.

~~**Funding**: This study was funded by a Wellcome Trust Training fellowship (109930/Z/15/Z) awarded to ELANGA N'DILLE Emmanuel.~~

Conflicts of Interest: The authors declare no conflicts of interest.

References

1. WHO (2019) World Malaria Report 2019, Geneva: World Health Organization; 2019. Licence: CC BY-NC-SA 3.0 IGO.

2. Briegel H (1990) Fecundity, metabolism, and body size in *Anopheles* (Diptera: Culicidae), vectors of malaria. Journal of medical entomology 27: 839-850.

Formatted: Font: 18 pt

Formatted: Font: (Default) Times New Roman, 12 pt

Formatted: Line spacing: 1.5 lines

Field Code Changed

Formatted: Font: (Default) Times New Roman, 12 pt, Italic

Formatted: Font: (Default) Times New Roman, 12 pt

3. Hurd H (2003) Manipulation of medically important insect vectors by their parasites. Annual review of entomology 48: 141-161.

4. Roitberg BD, Gordon I (2005) Does the *Anopheles* blood meal-fecundity curve, curve? Journal of Vector Ecology 30: 83.

5. Ribeiro J (2000) Blood- feeding in mosquitoes: probing time and salivary gland anti-haemostatic activities in representatives of three genera (*Aedes, Anopheles, Culex*). Medical and veterinary entomology 14: 142-148.

6. Islam A, Emran TB, Yamamoto DS, Iyori M, Amelia F, et al. (2019) Anopheline antiplatelet protein from mosquito saliva regulates blood feeding behavior. Scientific reports 9: 3129.

7. Thiévent K, Hauser G, Elaian O, Koella JC (2019) The interaction between permethrin exposure and malaria infection affects the host-seeking behaviour of mosquitoes. Malaria journal 18: 79.

8. Taylor P, Hurd H (2001) The influence of host haematocrit on the blood feeding success of *Anopheles stephensi*: implications for enhanced malaria transmission. Parasitology 122: 491-496.

9. Edman JD, Lynn HC (1975) Relationship between blood meal volume and ovarian development in *Culex nigripalpus* (Diptera: *Culicidae*). Entomologia experimentalis et applicata 18: 492-496.

10. Lehane MJ (2005) The biology of blood-sucking in insects: Cambridge University Press.

11. Diop MM, Chandre F, Rossignol M, Porciani A, Chateau M, et al. (2019) Sub-lethal insecticide exposure affects host biting efficiency of Kdr-resistant *Anopheles gambiae*. bioRxiv: 653980.

12. Bhatt S, Weiss D, Cameron E, Bisanzio D, Mappin B, et al. (2015) The effect of malaria control on *Plasmodium falciparum* in Africa between 2000 and 2015. Nature 526: 207.

13. Ranson H, N'guessan R, Lines J, Moiroux N, Nkuni Z, et al. (2011) Pyrethroid resistance in African anopheline mosquitoes: what are the implications for malaria control? Trends in parasitology 27: 91-98.

14. Strode C, Donegan S, Garner P, Enayati AA, Hemingway J (2014) The impact of pyrethroid resistance on the efficacy of insecticide-treated bed nets against African anopheline mosquitoes: systematic review and meta-analysis. PLoS medicine 11.

15. Martinez- Torres D, Chandre F, Williamson M, Darriet F, Berge JB, et al. (1998) Molecular characterization of pyrethroid knockdown resistance (kdr) in the major malaria vector *Anopheles gambiae ss*. Insect molecular biology 7: 179-184.

Formatted: Font: (Default) Times New Roman, 12 pt, Italic

Formatted: Font: (Default) Times New Roman, 12 pt

Formatted: Font: (Default) Times New Roman, 12 pt, Italic

Formatted: Font: (Default) Times New Roman, 12 pt

Formatted: Font: (Default) Times New Roman, 12 pt, Italic

Formatted: Font: (Default) Times New Roman, 12 pt

Formatted: Font: (Default) Times New Roman, 12 pt, Italic

Formatted: Font: (Default) Times New Roman, 12 pt

Formatted: Font: (Default) Times New Roman, 12 pt, Italic

Formatted: Font: (Default) Times New Roman, 12 pt

Formatted: Font: (Default) Times New Roman, 12 pt, French (France)

Formatted: Font: (Default) Times New Roman, 12 pt

Formatted: Font: (Default) Times New Roman, 12 pt, Italic

Formatted: Font: (Default) Times New Roman, 12 pt

Formatted: Font: (Default) Times New Roman, 12 pt, Italic

Formatted: Font: (Default) Times New Roman, 12 pt

Formatted: Font: (Default) Times New Roman, 12 pt, French (France)

Formatted: Font: (Default) Times New Roman, 12 pt

Formatted: Font: (Default) Times New Roman, 12 pt, Italic

Formatted: Font: (Default) Times New Roman, 12 pt

16. Ranson H, Jensen B, Vulule J, Wang X, Hemingway J, et al. (2000) Identification of a point mutation in the voltage- gated sodium channel gene of Kenyan *Anopheles gambiae* associated with resistance to DDT and pyrethroids. Insect molecular biology 9: 491-497.

17. Berticat C, Boquien G, Raymond M, Chevillon C (2002) Insecticide resistance genes induce a mating competition cost in *Culex pipiens mosquitoes*. Genetics Research 79: 41-47.

18. Saingamsook J, Yanola J, Lumjuan N, Walton C, Somboon P (2019) Investigation of Relative Development and Reproductivity Fitness Cost in Three Insecticide-Resistant Strains of *Aedes aegypti* from Thailand. Insects 10: 265.

19. Rowland M (1991) Behaviour and fitness of γHCH/dieldrin resistant and susceptible female *Anopheles gambiae* and *An. stephensi mosquitoes* in the absence of insecticide. Medical and Veterinary Entomology 5: 193-206.

20. Kumar K, Sharma AK, Kumar S, Patel S, Sarkar M, et al. (2011) Multiple insecticide resistance/susceptibility status of *Culex quinquefasciatus*, principal vector of bancroftian filariasis from filaria endemic areas of northern India. Asian Pacific journal of tropical medicine 4: 426-429.

21. Martins AJ, Bellinato DF, Peixoto AA, Valle D, Lima JBP (2012) Effect of insecticide resistance on development, longevity and reproduction of field or laboratory selected *Aedes aegypti* populations. PloS one 7: e31889.

22. Tchouakui M, Riveron JM, Djonabaye D, Tchapga W, Irving H, et al. (2018) Fitness Costs of the Glutathione S-Transferase Epsilon 2 (L119F-GSTe2) Mediated Metabolic Resistance to Insecticides in the Major African Malaria Vector *Anopheles Funestus*. Genes 9: 645.

23. Tchouakui M, Fossog BT, Ngannang BV, Djonabaye D, Tchapga W, et al. (2019) Investigation of the influence of a glutathione S-transferase metabolic resistance to pyrethroids/DDT on mating competitiveness in males of the African malaria vector, *Anopheles funestus*. Wellcome open research 4.

24. Riveron JM, Yunta C, Ibrahim SS, Djouaka R, Irving H, et al. (2014) A single mutation in the GSTe2 gene allows tracking of metabolically based insecticide resistance in a major malaria vector. Genome biology 15: R27.

25. Weedall GD, Mugenzi LM, Menze BD, Tchouakui M, Ibrahim SS, et al. (2019) A cytochrome P450 allele confers pyrethroid resistance on a major African malaria vector, reducing insecticide-treated bednet efficacy. Science translational medicine 11: eaat7386.

26. Menze BD, Riveron JM, Ibrahim SS, Irving H, Antonio-Nkondjio C, et al. (2016) Multiple insecticide resistance in the malaria vector *Anopheles funestus* from Northern Cameroon is mediated by metabolic resistance alongside potential target site insensitivity mutations. PloS one 11: e0163261.

27. Morgan JC, Irving H, Okedi LM, Steven A, Wondji CS (2010) Pyrethroid resistance in an *Anopheles funestus* population from Uganda. PLoS One 5: e11872.

28. Hunt R, Brooke B, Pillay C, Koekemoer L, Coetzee M (2005) Laboratory selection for and characteristics of pyrethroid resistance in the malaria vector *Anopheles funestus*. Medical and veterinary entomology 19: 271-275.

29. Riveron JM, Irving H, Ndula M, Barnes KG, Ibrahim SS, et al. (2013) Directionally selected cytochrome P450 alleles are driving the spread of pyrethroid resistance in the major malaria vector *Anopheles funestus*. Proceedings of the National Academy of Sciences 110: 252-257.

30. Wondji CS, Irving H, Morgan J, Lobo NF, Collins FH, et al. (2009) Two duplicated P450 genes are associated with pyrethroid resistance in *Anopheles funestus*, a major malaria vector. Genome research 19: 452-459.

31. Briegel H, Lea AO, Klowden MJ (1979) Hemoglobinometry as a method for measuring blood meal sizes of mosquitoes (Diptera: Culicidae). Journal of Medical Entomology 15: 235-238.

32. Livak KJ (1984) Organization and mapping of a sequence on the Drosophila melanogaster X and Y chromosomes that is transcribed during spermatogenesis. Genetics 107: 611-634.

33. Koekemoer L, Kamau L, Hunt R, Coetzee M (2002) A cocktail polymerase chain reaction assay to identify members of the *Anopheles funestus* (Diptera: *Culicidae*) group. The American journal of tropical medicine and hygiene 66: 804-811.

34. Klowden MJ, Lea AO (1978) Blood meal size as a factor affecting continued host-seeking by *Aedes aegypti* (L.). The American journal of tropical medicine and hygiene 27: 827-831.

35. Ramasamy M, Srikrishnaraj K, Hadjirin N, Perera S, Ramasamy R (2000) Physiological aspects of multiple blood feeding in the malaria vector *Anopheles* tessellatus. Journal of insect physiology 46: 1051-1059.

36. Takken W, van Loon JJ, Adam W (2001) Inhibition of host-seeking response and olfactory responsiveness in *Anopheles gambiae* following blood feeding. Journal of insect physiology 47: 303-310.

37. Jeanrenaud AC, Brooke BD, Oliver SV (2019) The effects of larval organic fertiliser exposure on the larval development, adult longevity and insecticide tolerance of zoophilic members of the *Anopheles gambiae* complex (Diptera: *Culicidae*). PloS one 14.

38. Owusu HF, Chitnis N, Müller P (2017) Insecticide susceptibility of *Anopheles* mosquitoes changes in response to variations in the larval environment. Scientific reports 7: 1-9.

39. Lyimo IN, Haydon DT, Mbina KF, Daraja AA, Mbehela EM, et al. (2012) The fitness of African malaria vectors in the presence and limitation of host behaviour. Malaria journal 11: 425.

40. Tchouakui M, Jacob Riveron M, Mugenzi LMJ, Djonabaye D, Wondji MJ, et al. (2020) Cytochrome P450 metabolic resistance (CYP6P9a) to pyrethroids imposes a fitness cost in the major African malaria vector *Anopheles funestus*. Genetics Society.

41. Okoye P, Brooke B, Hunt R, Coetzee M (2007) Relative developmental and reproductive fitness associated with pyrethroid resistance in the major southern African malaria vector, *Anopheles funestus*. Bulletin of Entomological Research 97: 599-605.

42. Rivero A, Vézilier J, Weill M, Read AF, Gandon S (2010) Insecticide Control of Vector-Borne Diseases: When Is Insecticide Resistance a Problem? PLoS Pathog 6.

43. Saeaue L, Phumala Morales N, Komalamisra N, Vargas RM (2011) Antioxidative systems defense against oxidative stress induced by blood meal in *Aedes aegypti*. Southeast Asian Journal of Tropical Medicineand Public Health 42: 542.

44. Graça-Souza AV, Maya-Monteiro C, Paiva-Silva GO, Braz GR, Paes MC, et al. (2006) Adaptations against heme toxicity in blood-feeding arthropods. Insect biochemistry and molecular biology 36: 322-335.

45. Telang A, Wells MA (2004) The effect of larval and adult nutrition on successful autogenous egg production by a mosquito. Journal of Insect Physiology 50: 677-685.

46. Edman JD, Scott TW (1987) Host defensive behaviour and the feeding success of mosquitoes. International Journal of Tropical Insect Science 8: 617-622.

47. Calvo E, Dao A, Pham VM, Ribeiro JMC (2007) An insight into the sialome of *Anopheles funestus* reveals an emerging pattern in anopheline salivary protein families. Insect Biochem Mol Biol 37: 164-175.

48. Calvo E, Mans BJ, Andersen JF, Ribeiro JM (2006) Function and evolution of a mosquito salivary protein family. J Biol Chem 281.

49. Elanga-Ndille E, Nouage L, Binyang A, Assatse T, Tene-Fossog B, et al. (2019) Overexpression of two members of D7 salivary genes family is associated with

Formatted: Font: (Default) Times New Roman, 12 pt, Italic

Formatted: Font: (Default) Times New Roman, 12 pt

Formatted: Font: (Default) Times New Roman, 12 pt, Italic

Formatted: Font: (Default) Times New Roman, 12 pt

Formatted: Font: (Default) Times New Roman, 12 pt, Italic

Formatted: Font: (Default) Times New Roman, 12 pt

Formatted: Font: (Default) Times New Roman, 12 pt, Italic

Formatted: Font: (Default) Times New Roman, 12 pt

Formatted: Font: (Default) Times New Roman, 12 pt, Italic

Formatted: Font: (Default) Times New Roman, 12 pt

Formatted: Font: (Default) Times New Roman, 12 pt, Italic

Formatted: Font: (Default) Times New Roman, 12 pt

Formatted: Font: (Default) Times New Roman, 12 pt, Italic

Formatted: Font: (Default) Times New Roman, 12 pt

Formatted: Font: (Default) Times New Roman, 12 pt, Italic

Formatted: Font: (Default) Times New Roman, 12 pt

Formatted: Font: (Default) Times New Roman, 12 pt, French (France)

Formatted: Font: (Default) Times New Roman, 12 pt

pyrethroid resistance in the malaria vector *Anopheles funestus ss* but not in *Anopheles gambiae* in Cameroon. Genes 10: 211.

50. Riveron JM, Ibrahim SS, Chanda E, Mzilahowa T, Cuamba N, et al. (2014) The highly polymorphic CYP6M7 cytochrome P450 gene partners with the directionally selected CYP6P9a and CYP6P9b genes to expand the pyrethroid resistance front in the malaria vector *Anopheles funestus* in Africa. BMC genomics 15: 817.

51. Samb B, Konate L, Irving H, Riveron JM, Dia I, et al. (2016) Investigating molecular basis of lambda-cyhalothrin resistance in an *Anopheles funestus* population from Senegal. Parasites & vectors 9: 449.

52. Wilding C, Weetman D, Rippon E, Steen K, Mawejje H, et al. (2015) Parallel evolution or purifying selection, not introgression, explains similarity in the pyrethroid detoxification linked GSTE4 of *Anopheles gambiae* and *An. arabiensis*. Molecular genetics and genomics 290: 201-215.

53. Wilding CS, Weetman D, Rippon E, Steen K, Mawejje H, et al. (2015) Parallel evolution or purifying selection, not introgression, explains similarity in the pyrethroid detoxification linked GSTE4 of *Anopheles gambiae* and *An. arabiensis*. Molecular genetics and genomics 290: 201-215.

Figures ~~legend~~titles

Fig~~ure~~ 1~~.~~: Effect of metabolic resistance on *An. funestus* mosquito weight. Distribution of genotypes of *L119F-GSTe2* (A) and *CYP6P9a*-R (B) markers according to the weight. ~~*An. funestus* mosquito weight~~

Fig~~ure 2: ~~2. Association between reistance markers and bloodfeeding. Distribution of *L119F-GSTe2* (A) and *CYP6P9a-R* (B) genotypes between blood-fed and unfed *An. funestus* mosquitoes.

Fig~~ure 3:~~, 3. Influence of metabolic resistance on blood meal size of *An. funestus* mosquitoes. ~~Blood meal size~~Effect of *L119F-GSTe2*(A) and *CYP6P9a*-R (B).~~*An. funestus* mosquitoes according to their *L119F-GSTe2* (A) and *CYP6P9a*-R (B) genotypes~~.

Fig~~ure 4:~~4. Comparative expression of salivary genes between CYP6P9a genotypes. Expression level of AAPP and some members of D7 family genes in CYP6P9a-RR and CYP6P9a-RS mosquitoes in comparison with *CYP6P9a* susceptible mosquitoes. The normalized relative expression of each gene against two housekeeping genes (RSP7 and Actin) is represented on the vertical axis. Letters a, b, c, d, e indicates the absence of significant difference in the expression level of each gene between the three types of mosquitoes. ~~The dotted line indicates genes expression level in *CYP6P9a* susceptible mosquitoes used as standard.~~

Formatted: Font: Bold

Formatted: Font: Bold

Formatted: Font: Bold
