# Supplementary material for: Influence of GST- and P450-based metabolic resistance to pyrethroids on blood feeding in the major African malaria vector Anopheles funestus
Source: PLoS One. 2020 Sep 18;15(9):e0230984. doi: 10.1371/journal.pone.0230984 (PMC7500606; doi:10.1371/journal.pone.0230984)
Supplement: Attachment — Submitted filename: Response to Reviewers.docx [file pone.0230984.s005.docx]

**Responses to Reviewers**

1. *I am still not convinced by the explanation given regarding the size of the mosquitoes. Stating that the excessive size of the mosquitoes is due to fresh mosquitoes being used rather than dried mosquito is not an acceptable explanation. One of the defining features of An. funestus is that these are relatively small mosquitoes, with this being used as a diagnostic feature for morphological identification, with mosquitoes typically being characterized as having a smaller wingspan than 3.5mm. Therefore, having mosquitoes 1mg per mosquito and larger is quite incongruent, as this is the size of a large An. gambiae complex mosquito. The argument is not helped by the authors quoting that their findings are in line with a study that assessed the size of An. gambiae. I understand that there is not much that can be done about this, but this anomaly must be acknowledged.*

**Answer**: We thank the reviewer for this remark he highlights once again. We agree with him that the value of the weight of our mosquitoes shows some potential anomalies that might have an important influence on the results of our study. Because there is not much that can be done about in the present study, we have however acknowledged this anomaly of the mosquito’s weight in the revised manuscript (see lines 430-442). If this type of study is repeated in our lab, we will hardly take into account the recommendation of the reviewer.

2. *The next issue that has not been satisfactorily answered by the authors is the effect of larval rearing. The authors state that “potential bias due to larval rearing conditions could not impact findings”. This is not true, as larval rearing conditions would affect the adult blood-feeding behaviour, regardless of how you segregate the mosquitoes. Very shortly afterwards, you state that “mosquitoes at all stages were pooled and reared in the same conditions”. This is what needs to be clarified. Did these same conditions include being fed a controlled amount of food? If yes, this must be described, if not it must be acknowledged.*

**Answer**: Even if our mosquitoes were reared and kept together under the same conditions and regardless their genotypes, we can recognize that since we did not strictly control the amount of food given to mosquitoes at larval ad adult stages, some biases could have been generated during the rearing. These potential biases might have to influence the weight of the mosquitoes and subsequently, some results obtained. This limitation of our study is now acknowledged in the revised manuscript (see lines 430-442). For further studies, it will be important to work under controlled condition to manage and reduce biases related to the weight of the mosquito.

3. *Although the authors have satisfactorily explained their motivation for crossing Fumoz and Fang, it is worth mentioning that this particular experimental set up may be flawed. It must be noted that An. funestus ss has two mitochondrial clades, and these have biological differences. Therefore, future studies need to check the clades of the resultant hybrids, and it is worthwhile checking the clades of the wild material. Although this cannot be attended to for this experiment, it is worth noting that when creating these hybrids, that mitochondrial clade hybrids can also be created, and if the authors continue to plan these type of experiments, this is something that must be taken into consideration.*

**Answer:** This suggestion of the reviewer is fully pertinent. This is something we did not pay attention to in our study. As the reviewer suggested for further studies crossing FUMOZ and FANG, we should assess the clades of the hybrids we will work with. Furthermore, this observation of the reviewer will allow us checking these clades of the resultant hybrids from crosses we are currently performed in our lab to select both full susceptible and resistant strains from local *An. funestus* ss population.
