# Supplementary material for: Influence of GST- and P450-based metabolic resistance to pyrethroids on blood feeding in the major African malaria vector Anopheles funestus
Source: PLoS One. 2020 Sep 18;15(9):e0230984. doi: 10.1371/journal.pone.0230984 (PMC7500606; doi:10.1371/journal.pone.0230984)
Supplement: Attachment — Submitted filename: PLoS one reviewer comments-PONE-D-20-07208.docx [file pone.0230984.s003.docx]

**Reviewer comments**

Although the authors have done work that is reasonably methodologically sound, I am not fully convinced by the conclusions, rather than having a problem with the findings. The key problem is that the argument of the association of the genotype with behaviours is not strong enough. I will outline some of the key issues that need to be addressed to strengthen the argument enough to be published. The experiments are largely sound, but there needs to be a large scale restructuring of the discussion and the conclusions drawn, as the arguments are not strongly convincing.

**Major comments:**

There is a substantial body of work about the effects of metabolic resistance and life history. The novel factor of your work is that your work involved wild specimens, where previous studies on metabolic resistance and its effects were on laboratory strains where the resistance mechanisms were characterised. Although the metabolic screening tools are useful, this does not mean that studies on metabolic resistance could not happen before it. The success rate of genotyping of the wild specimens (78% success rate) is concerning. The substantial amount of unsuccessfully genotyped individuals could have altered the findings. This must be acknowledged.

The authors need to guide the readers a little more. There are times in the methods section where it is confusing as to why the methods were being employed, but this was clarified in the results section. It would make the MS easier to read if the reason for the methodology was introduced prior to the description of the method.

During the PCR methods section, it seems that the different sections were written by different people. In some parts it is satisfactory, and in others, it seems that the methods come from a dissertation. PCR methods in a manuscript include concentrations and not volumes, and these sections are more suited for a standard operating procedure than a manuscript. Centrifugation data must be reported in rcf (g) not rpm. Please ensure that the language of the molecular methods is appropriate.

The weight of the *An. funestus* mosquitoes you describe is very high. In my experience mosquitoes weighing close to a milligram normally have wingspans of between 3.5 to 4mm, and these are very large *An. gambiae*. This result is therefore quite startling. It also leads to questions about the conclusions about genotype and size. Furthermore, it is also concerning that you talk about size, and the relationship between genotype and size, without describing how you controlled for the larval diet. If this was not controlled for, then genotype may be related to larval feeding propensity that resulted in larger adults, which is a slightly different situation. It may also simply be that there was feeding variation between cohorts. Therefore, if you cannot describe how larval feeding was standardised, then conclusions about size cannot be made.

Something that also jumps out is that CYP6P9-SS adults are bigger. This is quite unusual, as larger mosquitoes tend to be more tolerant of insecticides, or that resistant phenotypes are associated with larger body sizes (eg: Osuwu et al: Sci Rep. 2017 Jun 16;7(1):3667, Jeanrenaud et al, PLoS One. 2019 Apr 18;14(4):e0215552). Such an unusual result must be discussed.

Line 375-396: Impact of metabolic resistance on blood feeding success

This particular argument does not hold water. Firstly, you have just given the definition of an anautogenous mosquito. Secondly, you hypothesize that CYP6P9 could affect feeding because of teneral reserves. You have not examined this, and crucially, as have described above, you do not describe how feeding was controlled for and this is crucial for comments on size. Also, as I have described above, the mosquitoes you have described are extremely large for *An. funestus*, so I don’t think poor teneral reserves can account for the findings you have described. Related to this, later there are discussions about resistance and blood feeding, hypothesising that the smaller resistant mosquito (which is unusual in itself) needs to take more blood because they have lower teneral reserves. This line of argument is not well supported as there was no evidence that food quantity was controlled, and therefore there cannot be arguments about teneral reserves. Teneral reserves are not only represented by size, and if there is an argument to made about this, there are simple calorimetric assays described in the MR4 manual to measure this. Finally, the fact that these mosquitoes seem to be very large for their species, it seems problematic to ascribe this behaviour to poor teneral reserves.

Line 398-417	Impact of metabolic resistance on probing time and feeding duration

Besides the comment that I have made on the document that it is not worth discussing results that are not statistically significant, there is another methodical flaw that is revealed in this discussion. By the authors’ admission, there were not a large amount of resistant heterozygotes. Although crosses were performed to generate heterozygotes, if Fumoz was available for crossing, surely uncrossed Fumoz would have been a useful source of RR mosquitoes? Is there a reason why RR and SS individuals were not obtained for study from the original strains?

Line 418-450: Impact of metabolic resistance on blood meal volume

There are some issues in this section.

Line 427-431. The authors describe that “the positive association CYP6P9a-R resistant allele and the volume of blood meal is a bit surprising knowing that activity of P450 monoxygenases as well as blood meal digestion, have been reported to generate an excess production of reactive oxygen species (ROS) increasing oxidative stress which could induce several damages in the mosquito’s system that can result to death [38,39]. This is not really that surprising if you consider that the resistant *An. funestus* strains Fumoz and in particular Fumoz R has a marked capacity to cope with oxidative stress, both phenotypically and due to increased gluthatione peroxidase and catalase activity (Oliver and Brooke PLoS One. 2016 Mar 10;11(3):e0151049): Your reference cites information about oxidative stress in *Aedes*. There are better references, as there is work on oxidative stress in Anopheles, not only the previously mentioned which shows that oxidative stress in *An. funestus* specifically is quite different to *Aedes*. Champion and Xu (Sci Rep 8, 13054 (2018)) show the complexity of the interaction between oxidative stress, insecticide resistance and fecundity in *An. gambiae*. Crucially, what they show a relationship between oxidative stress and resistance, with resistant *An. gambiae* also has a greater oxidative stress capacity. Over and above this, epsilon class GSTs are associated with oxidative stress defence. In the selections, did these genotypes segregate separately (was this checked), because it will be difficult to discuss oxidative stress in relation to CYP6P9 (where P450s do increase oxidative stress) if the effect of the GSTe2 in the same individual has not been considered.

Another major issue that I have with this argument has to do with the argument about fitness costs in *An. funestus*. Although your reference for this is a manuscript where fitness costs are noted, this manuscript examined it in crosses, as you have done in this experiment. However, that study and the present study ignore the work of Okoye et al (Bull Entomol Res. 2007 Dec;97(6):599-605), which did not find a fitness cost to resistance in Fumoz. Their conclusion is supported by the fact that Fumoz-R maintains high deltamethrin resistance intensity without selection. Therefore, a fitness cost cannot be unambiguously assigned to the resistant funestus.

The studies on salivary gene expression are problematic. I assumed that there would not be a different as the error bars (if they are error bars and not standard deviation) are huge. This is concerning and this is not a good quality result for publication. Is there an explanation for this?

**Minor comments**

Throughout the document: change homo or heterozygote to heterozygous where it is used as an adjective rather than a noun.

There are numerous small comments annotated on the manuscript that must be addressed.
